# Biomimetic material degradation for synergistic enhanced therapy by regulating endogenous energy metabolism imaging under hypothermia

**DOI:** 10.1038/s41467-022-32349-2

**Published:** 2022-08-05

**Authors:** Kai Cheng, Bo Liu, Xiao-Shuai Zhang, Ruo-Yun Zhang, Fang Zhang, Ghazal Ashraf, Guo-Qing Fan, Ming-Yu Tian, Xing Sun, Jing Yuan, Yuan-Di Zhao

**Affiliations:** 1 Britton Chance Center for Biomedical Photonics at Wuhan National Laboratory for Optoelectronics-Hubei Bioinformatics & Molecular Imaging Key Laboratory, Department of Biomedical Engineering, College of Life Science and Technology, Huazhong University of Science and Technology, Wuhan, 430074 Hubei P. R. China; 2 Key Laboratory of Biomedical Photonics (HUST), Ministry of Education, Huazhong University of Science and Technology, Wuhan, 430074 Hubei P. R. China

**Keywords:** Nanotechnology in cancer, Imaging techniques and agents, Imaging techniques

## Abstract

Inefficient tumour treatment approaches often cause fatal tumour metastases. Here, we report a biomimetic multifunctional nanoplatform explicitly engineered with a Co-based metal organic framework polydopamine heterostructure (MOF-PDA), anethole trithione (ADT), and a macrophage membrane. Co-MOF degradation in the tumour microenvironment releases Co^2+^, which results in the downregulation of HSP90 expression and the inhibition of cellular heat resistance, thereby improving the photothermal therapy effect of PDA. H_2_S secretion after the enzymatic hydrolysis of ADT leads to high-concentration gas therapy. Moreover, ADT changes the balance between nicotinamide adenine dinucleotide/flavin adenine dinucleotide (NADH/FAD) during tumour glycolysis. ATP synthesis is limited by NADH consumption, which triggers a certain degree of tumour growth inhibition and results in starvation therapy. Potentiated 2D/3D autofluorescence imaging of NADH/FAD is also achieved in liquid nitrogen and employed to efficiently monitor tumour therapy. The developed biomimetic nanoplatform provides an approach to treat orthotopic tumours and inhibit metastasis.

## Abstract (web summary)

Metal organic frameworks (MOF) coated with mammalian cell membranes have good biocompatibility. Here, the authors develop a cobalt based hydrogen sulphide producing MOF cloaked with a macrophage membrane and show that the subsequent system can reduce tumour growth in mice.

## Introduction

The occurrence and development of malignant tumours are rapid processes, making suppressing metastasis and treating tumours particularly critical issues in addition to early diagnosis and treatment^1–4^. Currently, breast cancer has become one of the top three cancers with the highest morbidity worldwide and is universally known as the “pink killer”^5^. It has been well documented that breast cancer is prone to distant metastasis, which, in turn, leads to multiple diseases and organ failure^6^. In addition, the mortality rate after metastasis reaches as high as 80%^7^, thus seriously threatening the lifespan of patients, especially women. Breast cancer treatments mainly include surgical resection^8,9^ radiotherapy^10,11^, and chemotherapy^12–14^. Although chemotherapy has made some important progress^15–17^, the resulting poor effects from low drug specificity are still a major challenge for the treatment of breast cancer.

Recently, treatment with nanomedicines has attracted much research attention^18–20^ owing to the multifunctional nanoplatforms that play a pivotal role in various clinical drugs, such as adriamycin liposomes^21^, albumin paclitaxel^22^, and fluorouracil implants^23^. A common feature of these drugs is that they can markedly improve the circulation time of the functionalized nanodrugs in the body, thereby achieving efficient enrichment in the lesions. Therefore, developing effective nanoplatforms can be a promising method for breast cancer treatment.

Heat shock protein 90 (HSP90) is a critical molecular chaperone protein that widely exists in the human body and is vital to maintain homeostasis^24^. The level of HSP90 in cancer tissues (liver, colorectal, breast, and other organs)^25–27^ is high, and since it can be secreted from tumour cells, tumour invasion and metastasis can be accelerated through blood circulation^28^. Therefore, HSP90 has also been employed as a promising tumour biomarker^29^. Photothermal therapy (PTT), as a primary tumour treatment approach, possesses apparent advantages such as being minimally invasive^30^, rapid^31^, and hypotoxic^32^. However, as a stressor, PTT can also rapidly induce HSP generation^33–35^, which leads to its higher concentration at the tumour site^36^. This will stimulate an enhancement in the body’s ability to handle stress, especially heat tolerance, ultimately resulting in lower PTT efficiency. Therefore, reducing heat resistance is an effective way to improve the effects of PTT. Gases, such as NO, have emerged as a green tumour treatment modality with great application prospects. As one of the three high-profile endogenous gas media^37–39^, H_2_S participates in many physiological processes. Decades of studies have shown that H_2_S is equipped with two distinct physiological effects: pro-apoptosis at high concentrations and growth promotion at low concentrations^40^. The pro-apoptotic effect mainly results from uncontrollable internal cellular acidification, which induces cell cycle arrest to promote apoptosis and achieves an antitumour effect^40^. It is worth noting that H_2_S has a short blood half-life, poor stability, and no specific ability to target tumour tissues^40^. Therefore, it is particularly important to exploit effective nanoplatforms that can transport H_2_S to the lesion site for its accurate release.

Metal-organic frameworks (MOFs) are a type of coordination polymer that has undergone rapid development in recent years and possess the characteristics of high porosity, low density, large specific surface area, regular pores, adjustable pore size and diversity^41–43^. Hence, in the field of nanomedicine, MOFs are one of the best drug carriers. Moreover, biomimetic functional carriers for drug delivery, represented by tumour cells, red blood cells, white blood cells and stem cells^44–47^, have gradually attracted the attention of researchers. By modification with the abovementioned cell membranes, these functional platforms are endowed with excellent biocompatibility and corresponding cell stimulation, which are capable of achieving homology or evasion effects. As one of the three central immune cells in the human body, macrophages are abundantly enriched at tumour sites^48^, which is conducive to the promotion of tumour metastasis and the formation of metastatic foci^49^. Targeted recognition can be achieved through interactions between the abundant α4 and β1 integrin proteins on the macrophage surface with the highly expressed vascular cell adhesion molecule VCAM-1 on tumour cells^50^.

Herein, a biomimetic functional nanoplatform with a macrophage membrane is designed. In this nanoplatform, polydopamine (PDA) is employed as the core owing to the strong chelation of its catechol groups to metal ions, and Co-MOF is grown as the shell, making a heterogeneous structure. Moreover, the H_2_S-releasing precursor, anethole trithione (ADT), is loaded into the pores of the MOF and gaps of the heterostructure via a one-pot method. Finally, modification with a macrophage membrane improves the specificity and stability of the nanocarrier. The biomimetic nanocarrier is designed to show the following characteristics. (1) As an organic polymer, PDA possesses prominent photothermal conversion efficiency; however, the thermal effect will cause a dramatic increase in HSP90 content so that it is expressed at a relatively high level in the tumour site, which ultimately reduces the PTT effect. Fortunately, this problem is properly resolved by the degradation of Co-MOF in an acidic environment. It is found that the released free Co^2+^ can downregulate the expression of HSP90, resulting in reduced tumour cell thermal tolerance, thereby enhancing the effects of PTT. (2) After the disintegration of Co-MOF, the loaded ADT will be released in large quantities. As a precursor of H_2_S, ADT can be catalysed by enzymes highly expressed in breast cancer cells to produce high concentrations of H_2_S, which in turn inhibits tumour growth and metastasis. (3) It is also demonstrated that, at the tumour site, ADT can chemically react with nicotinamide adenine dinucleotide (NADH) to cause a dynamic imbalance in the nicotinamide adenine dinucleotide/flavin adenine dinucleotide (NADH/FAD) ratio. In addition, NADH plays a substantial role in energy metabolism. As a carrier and electron donor of biological hydrogen, NADH transfers energy to synthesize ATP through oxidative phosphorylation on the inner mitochondrial membrane^51^. This means that the synthesis of ATP is affected, the energy supply for cells is reduced, and NADH is consumed in large quantities, which results in a certain degree of tumour growth inhibition, therefore achieving starvation therapy. During this process, enhanced autofluorescence imaging of NADH and FAD under deep hypothermia can be used to determine the enrichment of the probe at the tumour site over time to guide tumour treatment. (4) Considering the abnormally rich vasculature of the tumour, this biomimetic nanoplatform can become enriched in the tumour microenvironment by targeting through the integrin on the macrophage membrane wrapping. The integrin interacts precisely with highly expressed vascular cell adhesion molecules of tumour cells. This kind of targeting allows the probe to enter tumour cells more quickly. Additionally, the macrophage membrane can also act as camouflage, which is verified to improve the biocompatibility of the probe and reduce immune cell phagocytosis of the nanoplatform. This biodegradable biomimetic nanoplatform has the specific capabilities of targeted drug delivery and biosafety, which lead to enhanced photothermal synergistic gas-starvation therapy to treat orthotopic breast cancer with a highly effective inhibitory effect on metastatic tumours. This nanoplatform will provide research ideas for future cancer treatments and modes of tumour metastasis inhibition.

## Results

### Synthesis and characterization of PCoA@M

**Fig. 1:**
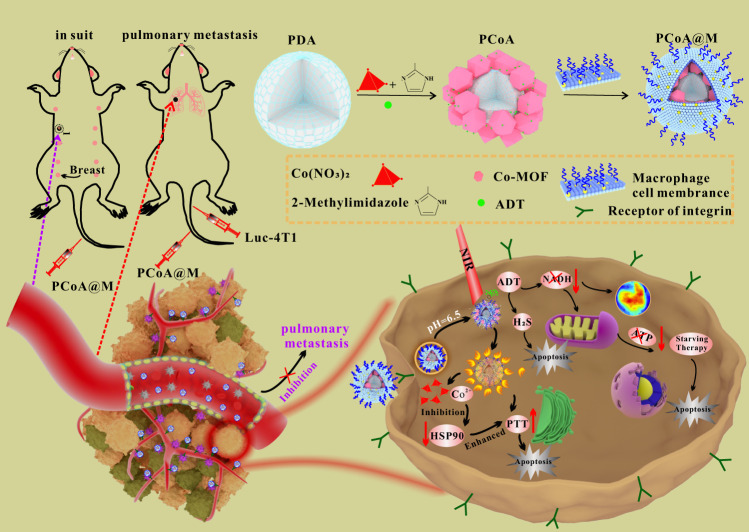
Synthesis and antitumor mechanism of PCoA@M. 1 PDA, Co-MOF, and ADT were synthesized by a one-pot method and modified with macrophage membranes to form PCoA@M. 2 PCoA@M became enriched in the tumour microenvironment through integrin targeting. 3 PCoA@M enhanced PTT by inhibiting HSPs, degrading under acidic conditions to release drugs, reducing NADH generation, and achieving PTT-gas synergistic starvation therapy. 4 PCoA@M inhibited tumour growth and metastasis.

**Fig. 2:**
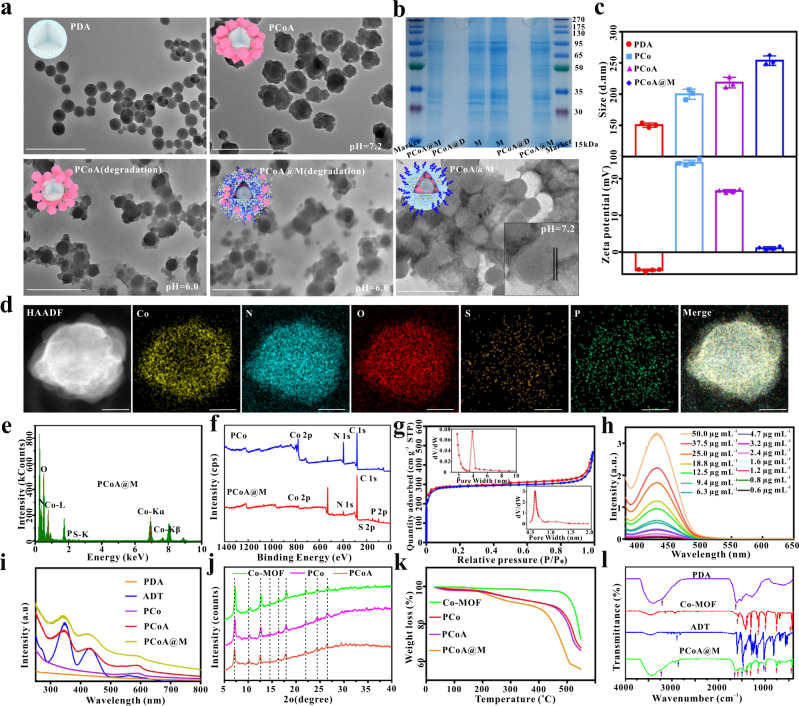
Characterization of the physicochemical properties of the probes. **a** Transmission electron microscopy (TEM) images of PDA, PCoA, and PCoA@M at pH = 7.2 and PCoA and PCoA@M at pH = 6.0. Scale bar: 500 nm. **b** SDS–PAGE protein analysis of the macrophage membrane, PCoA@M, and PCoA@D. **c** Hydrated particle sizes and zeta potentials of PDA, PCo, PCoA, and PCoA@M. Data are presented as the means ± s.d. (size: *n* = 3 independent samples, zeta potential measurements: *n* = 4 independent samples). **d**, **e** Elemental mapping and energy dispersive spectra of PCoA@M. **f** XPS of PCo and PCoA@M. Scale bar: 50 nm. **g** Nitrogen adsorption-desorption isothermal curve of PCo. Inset: pore size distribution. **h** Absorption spectra of different concentrations of ADT. **i** Absorption spectra of PDA, PCo, PCoA, and PCoA@M. **j** X-ray powder diffraction analysis of Co-MOF, PCo, and PCoA. **k** Thermogravimetric analysis of Co-MOF, PCo, PCoA, and PCoA@M. **l** Infrared spectra of PDA, Co-MOF, ADT, and PCoA@M.

Here, PDA with a controllable size was fabricated. Due to the strong ability of PDA to chelate with metal ions, a porous frame structure formed on the surface of PDA after binding to Co^2+^ as the metal unit. Next, the gas prodrug was loaded into the frame holes and heterostructure, which was coated and modified by a macrophage membrane to form a stable nanoplatform. This nanoplatform was delivered to the tumour site and degraded in the acidic microenvironment to release Co^2+^, thus downregulating HSP90 expression to enhance the PTT effect. Concurrently, the gas prodrug was induced to generate a high concentration of H_2_S, which promoted tumour cell apoptosis (Fig. 1). The particle size of PDA was approximately 90 nm (Fig. 2a), which then increased to ~160 nm with a certain amount of drug-loaded Co-MOF (Fig. 2a). After modification with the macrophage membrane, PCoA@M was negatively stained with 2% ammonium molybdate (pH 7.4). The results highlighted the cell membrane (Fig. 2a), which presented as a white circle, and the size of the nanomaterial slightly increased further (~180 nm). Next, the process of Co-MOF coating on the surface of PDA was investigated. The results showed that when the dosage of PDA was 0.5, 0.6, 0.7, 0.8, and 1 mg, without changing the other parameters, the amount of free hexagonal Co-MOF continuously decreased with increasing PDA dosage (Supplementary Fig. 1). When the dosage reached 1 mg, there was almost no free Co-MOF, indicating that the MOF had mostly chelated on the surface of PDA, which indicated that the heterostructure synthesis was successful. After multiple centrifugation and purification steps, the characteristic absorption peak of ADT disappeared in the supernatant (Supplementary Fig. 2) but not the precipitate, indicating that ADT was successfully loaded into the nanoplatform. Additionally, the degradation properties of the nanoparticles (stored under acidic conditions for 2 h) were also analysed. Structural collapse of the MOF in PCoA and PCoA@M distinctly occurred, while PDA remained comparatively intact (Fig. 2a). This result showed the excellent acid sensitivity of the probe. Gel electrophoresis protein experiments (Fig. 2b) did not show the appearance of a band from the PCoA@D group after treatment with lysate, but the protein profile of PCoA@M was consistent with the macrophage cell membrane, indicating that the fused membrane protein was well preserved. This finding also meant that the integrin protein on the cell membrane could be expected to guarantee the targeting ability of the probe. Dynamic light scattering showed a slightly larger size of the PDA (~150 nm) (Fig. 2c) due to the hydrated particle size. Characteristic elemental mapping analysis of PCo and PCoA@M was subsequently performed. PCo exhibited a distinct and well-proportioned distribution of the elements N and Co (Supplementary Figs. 3 and 4), and relatively obvious N, Co, S, and P signals were observed in the PCoA@M nanoparticle analysis (Fig. 2d, e). These data fully demonstrated the formation of the Co-MOF on the surface of PDA and the functional modification with the cell membrane. X-Ray photoelectron spectroscopy (XPS) analysis was performed on the PDA, PCo (PDA@Co-MOF), PCoA (PDA@Co-MOF/ADT), and PCoA@M (PDA@Co-MOF/ADT@membrane) samples to detect the composition and chemical state of the surface elements. Notably, the characteristic element N was present in PDA, Co was found in Co-MOF, S was detected in the prodrug ADT, and P was observed in the cell membrane. The respective characteristic signal peaks appeared as follows: PDA (N 1 s: 400.4 eV, Supplementary Fig. 5), PCo (N 1 s: 399.03 eV, Co 2p_3/2_: 780.73 and 786.68 eV, Co 2p_1/2_: 796.73 and 802.63 eV, Fig. 2f and Supplementary Fig. 6), PCoA (N 1 s: 398.933 eV, Co 2p_3/2_: 780.983 and 786.833 eV, Co 2p_1/2_: 796.583 and 802.283 eV, S 2p: 165.0 eV, Supplementary Fig. 7), and PCoA@M (N 1 s: 399.35 eV, Co 2p_3/2_: 781.05 and 787.35 eV, Co 2p_1/2_: 797.25 and 802.70 eV, S 2p: 165.5 eV, P 2p: 133.45 eV, Fig. 2f and Supplementary Fig. 8). There were relatively weak signals from Co and S in PCoA@M because the maximum depth of detection by XPS is approximately 10 nm and the thickness of the cell membrane in PCOA@M is approximately 8 nm, leading to poor detection sensitivity of the Co-MOF chelated on the surface of PDA with the loaded prodrug ADT. Next, the mesoporous and microporous structures of PCo were characterized by Brunner–Emmett–Teller (BET) measurements. The nitrogen adsorption-desorption isotherm of PCo (Fig. 2g) showed a typical type IV isotherm with a hysteresis loop. There was a clear capillary condensation phenomenon in the lower relative partial pressure range (P/P_0_ = 0.14–0.45) resulting from uniform mesopores. The total specific surface area and pore volume were 1133.7655 m^2^ g^−1^ and 0.720007 cm^3^ g^−1^, respectively, and the average pore diameter was 2.54 nm. The absorption spectra of the different modified probes showed that, compared with PDA and PCo, the ADT-loaded heterostructure exhibited two additional absorption peaks (345 and 430 nm) corresponding to ADT (Fig. 2h), which also appeared in PCoA@M. According to the absorption curves (Fig. 2i and Supplementary Figs. 9–11), the drug loading rate of ADT was calculated to be 3.4%.

Furthermore, the membrane protein content in PCoA@M was determined by bicinchoninic acid (BCA) assay. The results showed that 0.9 mg of PDA contained 0.16 mg of membrane protein (Supplementary Fig. 12). The zeta potentials of PDA, PCo, PCoA, and PCoA@M were approximately −4.9, 24.4, 16.6, and 1.1 mV, respectively (Fig. 2c). These values were obtained because PDA possesses a large number of negatively charged hydroxyl groups, MOF presents a mass of positively charged Co skeletons, and the macrophage cell membrane contains many negatively charged phosphate groups. All of the above results demonstrated the successful loading of ADT and modification with the macrophage cell membrane. The X-ray diffraction (XRD) analyses exhibited similar diffraction peaks in the PCoA, PCo, and Co-MOF samples, indicating the successful deposition of Co-MOF on the PDA surface, and the crystal structure of Co-MOF was not affected by ADT loading (Fig. 2j). Thermogravimetric analysis of various fabricated heterostructures (Fig. 2k) demonstrated that the degradation rate increased significantly (PCoA@M > PCoA>PCo>Co-MOF) with increasing temperature (30 to 550 °C), possibly because the thermal degradation temperature of the macrophage wrapping on PCoA@M was lowered to approximately 200 °C, while that of ADT was elevated to approximately 450 °C. Moreover, the FT-IR spectra of the different modified nanomaterials were also acquired (Fig. 2l). For PDA, a C=C skeleton peak at 1600 cm^−1^ and a stretching vibration peak from aromatic hydrogens in aromatic hydrocarbons at 3000–3200 cm^−1^ were observed; for Co-MOF, the Co-N stretching vibration peaks and plane bending and stretching vibration peaks at 420, 500~1350, and 1350~1500 cm^−1^ arose; and for ADT, a carbon-sulphur double bond peak emerged at 1200 cm^−1^. Our findings showed that PCoA@M possessed all of the aforementioned characteristic peaks, which further confirmed the successful fabrication of PCoA@M.

### Stability, cytotoxicity, phagocytosis and colocalization of the probes

**Fig. 3:**
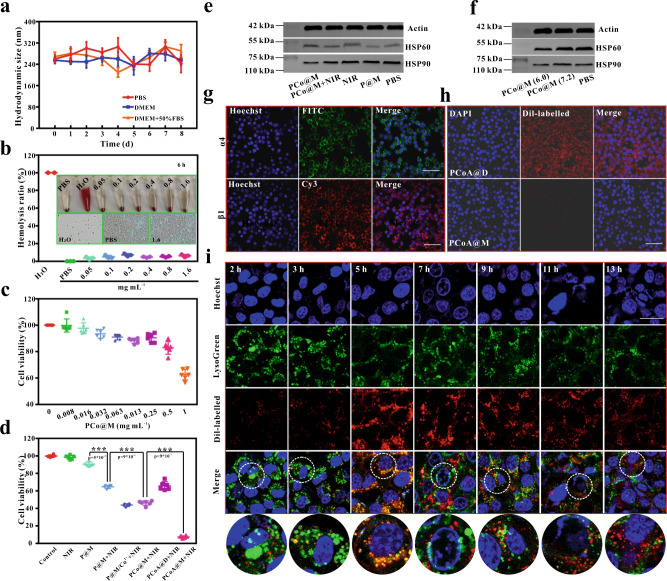
Biocompatibility and targeting of the probes at the cellular level. **a** Hydrodynamic particle size of PCoA@M over time in different media. Data are presented as the means ± s.d. (*n* = 3 independent samples). **b** Haemolysis rate of erythrocytes after incubation with different concentrations of probe. Inset: bright field image and micrograph of the cell smear made with the precipitate after incubation. **c** CCK-8 cytotoxicity of 4T1 cells incubated with different concentrations of PCo@M. Data are presented as the means ± s.d. (*n* = 6 independent samples). **d** Survival rate of 4T1 cells incubated and treated with different probes. Data are presented as the means ± s.d. (*n* = 6 independent samples). Statistical differences were calculated using two-tailed Student’s *t* test, *: *p* < 0.05; **: *p* < 0.01; ***: *p* < 0.001. **e**, **f** Western blot analysis of HSP a**f**ter treating 4T1 cells with different probes and at different pH values. **g** Immunofluorescence imaging for the detection of α4 and β1 antigens on the macrophage membrane. Images are representative of three biologically independent samples. Scale bar: 50 μm. **h** Confocal imaging of Dil-labelled PCoA@M and PCoA@D incubated with RAW 264.7 cells for 5 h. Scale bar: 50 μm. **i** Colocalized images of Dil-labelled PCoA@M and 4T1 cells incubated for 2, 3, 5, 7, 9, 11, and 13 h. Scale bar: 50 μm.

Stability is a prerequisite for probe application. Therefore, an in vitro stability simulation experiment was conducted with PCoA@M. PCoA@M was incubated with H_2_O, PBS, NaCl, Dulbecco’s modified Eagle’s medium (DMEM), 1640, 10% foetal bovine serum (FBS), 50% FBS, and 100% FBS without notable precipitation in the solution (Supplementary Fig. 13). At room temperature, PCoA@M dispersed well in PBS, DMEM, and DMEM + 50% FBS and displayed high polydispersity index (PDI) stability with time (Supplementary Fig. 14), the zeta potential had a negligible change (Supplementary Fig. 15), and the particle size exhibited no significant difference (Fig. 3a), indicating that PCoA@M was stable; these results laid the foundation for the application of the probe. Since the probe enters the body through blood circulation, blood compatibility was also investigated with haemolysis experiments with red blood cells (RBCs). The results revealed 100% haemolysis of RBCs in ultrapure water and 0% in PBS (Fig. 3b and Supplementary Fig. 16), which was due to the difference in osmotic pressure of these two solvents. Almost all of the RBCs sank when the systems contained the probe, and the supernatant exhibited a negligible change before and after probe addition. Moreover, when 1.6 mg mL^−1^ probe was added for 6 h of incubation, the haemolysis rate remained low. The microscopic images of the cell smears revealed that the cell morphology was regular (Fig. 3b, inset), which is in accordance with PBS incubation, while in pure water, the cells were in a fragmented state. All of these results indicated that PCoA@M had good biocompatibility, stability and a slight impact on RBCs, which suggested the safety of this probe for subsequent experiments in vivo.

Next, to ensure the biosafety of PCoA@M, cell counting kit-8 (CCK-8) experiments were used to evaluate the cytotoxic effects of PCo@M on 4T1 and C26 cells. A high probe concentration (500 μg mL^−1^) exhibited low toxicity to these cells (Fig. 3c and Supplementary Fig. 17). Furthermore, the targeted photothermal and gas starvation synergistic therapy effects of the probe in 4T1 cells was analysed. The results showed that the survival rate of the cells was approximately 98.45% after NIR treatment alone (Fig. 3d and Supplementary Figs. 18–19). When the concentration of PDA was 64 µg mL^−1^, the survival rate reached 90.78%, and the cells treated with P@M + NIR exhibited a decline in survival (64.19%), which was significantly different from the P@M group (*p* < 0.001), signifying a better killing effect resulting from PTT. When cells were treated with P@M/Co^2+^+NIR, the cell survival rate decreased to 43.1%, which was different from that in the P@M + NIR group, indicating that Co^2+^ played an important role to enhance PTT treatment. Cells treated with PCo@M + NIR revealed a large decline in the cell survival rate (45.25%), which was also significantly better than that of the P@M + NIR group (*p* < 0.001). This result might be due to the degradation of Co-MOF, which released Co^2+^ to further improve the effects of PTT. The survival rate of 4T1 cells incubated with PCoA@M was only 8.25%, and there was a significant difference (*p* < 0.001) compared with the single treatment group. Notably, the survival rate of the cells in the PCoA@D + NIR group was 65.25%, demonstrating that the wrapped macrophage membrane played an important role in the therapeutic effect. As depicted above, the acid-degraded Co^2+^ boosted the sensitivity of the tumour cells to PTT and further improved the PTT effects. To verify the mechanism of Co-MOF in the enhanced PTT, a study on the expression of HSP90 in 4T1 tumour cells was conducted under various conditions. The results revealed that among the P@M, NIR, and PBS groups, there was no marked difference in the cell expression of HSP90 (Fig. 3e and Supplementary Fig. 20). The expression of HSP90 in the PCo@M + NIR group decreased noticeably (*p* < 0.01) compared to that in the NIR group, which might be due to its downregulation by PCo@M. Compared with that in the P@M group, the expression level of HSP90 in the PCo@M group was low (*p* < 0.01), so we hypothesized that Co-MOF played a role in HSP expression. The pH of DMEM high-glucose medium was previously detected to be approximately 6.0. To further verify our hypothesis, we incubated Co-MOF with 4T1 cells for 6 h at pH 7.2 and 6.0. The western blot results showed that the expression of HSP in the Co-MOF group incubated at pH 6.0 decreased significantly (Fig. 3f and Supplementary Fig. 21), while the expression of HSP in the Co-MOF group incubated at pH 7.2 was not significantly different from that in the PBS group. Therefore, we speculated that Co^2+^ reduced the expression of HSP. Because the targeting function was fulfilled by the macrophage membrane, the integrin proteins α4 and β1 on the surface of RAW 264.7 cells were detected by immunofluorescence and flow cytometry. The strong green fluorescence generated from the secondary antibody against α4 integrin appeared (Fig. 3g), and the red fluorescence from the β1 integrin secondary antibody was also observed, which was consistent with the flow cytometry analysis (Supplementary Figs. 22 and 23). These data revealed that on the surface of the macrophage membrane, many α4 and β1 integrin proteins were expressed. Therefore, by combining the vascular cell adhesion molecule VCAM-1 on tumour cells with the integrin protein, the tumour-targeting recognition effect of the macrophage membrane could be achieved. These findings illustrated that the macrophage membrane-functionalized probe also possessed an effect to target blood vessels. Dil-labelled PCoA@M and PCoA@D were then incubated with RAW 264.7 cells for 5 h. The observed red fluorescence in the PCoA@D group was stronger than that in the PCoA@M group (Fig. 3h), proving that the probe modified with the macrophage membrane could produce a good camouflage effect, thereby reducing phagocytosis by macrophages. To verify the lysosomal escape of PCoA@M, lysosomal colocalization experiments were conducted by incubating Dil-labelled PCoA@M with 4T1 cells for different lengths of time. At 2 h, the red fluorescence was weak (Fig. 3i), which might be due to reduced phagocytosis of the Dil-labelled PCoA@M by the cells. At 5 h, the red fluorescence of the probe largely overlapped with the green fluorescence of the lysosomes. The overlapping areas gradually decreased with time and were clearly separated at 13 h, further indicating the lysosomal escape ability of Dil-labelled PCoA@M was achieved with the extension of incubation time. All of the above results were confirmed by quantitative fluorescence colocalization analysis (Supplementary Fig. 24), where the intensity first approached and gradually become inconsistent with distance.

**Fig. 4:**
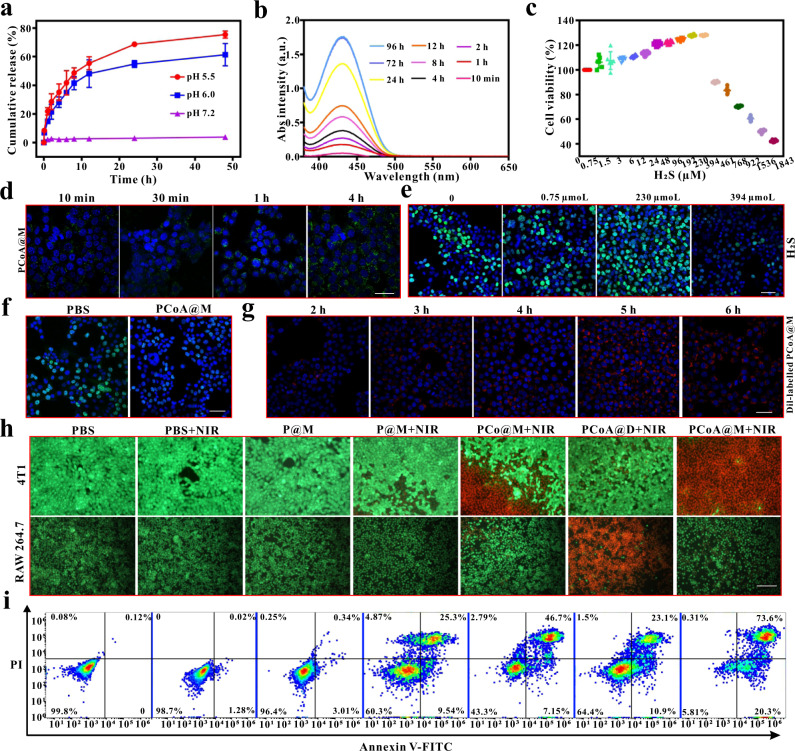
Therapeutic effects of the probes at the cellular level. **a** Cumulative release of Co^2+^ at different times and pH values. Data are presented as the means ± s.d. (*n* = 3 independent samples). **b** Absorption (Abs) spectra of ADT release at different times. **c** CCK-8 cytotoxicity assessment of 4T1 cells incubated with different concentrations of H_2_S. Data are presented as the means ± s.d. (*n* = 6 independent samples). **d** Confocal images of 4T1 cells after incubation with PCoA@M + WSP-1 for different lengths of time. Scale bar: 50 μm. **e**, **f** EdU proliferation of 4T1 cells coincubated with different concentrations of H_2_S, PBS and PCoA@M. Scale bar: 50 μm. **g** Confocal images of 4T1 cells after incubation with Dil-labelled PCoA@M for different lengths of time. Scale bar: 50 μm. **h** Fluorescence images of calcein/PI-stained 4T1 and RAW 264.7 cells incubated with different probes under different conditions. Scale bar: 100 μm. **i** Flow cytometry analysis of 4T1 cell apoptosis under the different conditions mentioned above.

The function of PCoA@M lies in the degradation of Co-MOF. Therefore, the release of Co^2+^ was studied by detecting Co by atomic absorption spectrophotometry. The results showed that when the pH was 5.5, the release of Co^2+^ increased with time (Fig. 4a and Supplementary Fig. 25) and reached 55.42% drug loading at 12 h; when the pH was 6.0, the release of Co^2+^ was basically the same as that at pH 5.5, but the amount released (48.15%) was slightly smaller; and when the pH was 7.2, the amount of released Co^2+^ changed little with time and was only 2.63% at 12 h, which was much lower than the amounts released at both pH 5.5 and 6.0. This result fully demonstrated that Co-MOF is responsive to low pH, and the lower the pH was, the faster the degradation rate of Co-MOF. The release of ADT depends on MOF degradation, so an ADT release experiment at pH=6.0 was performed based on the above results of MOF degradation. At pH 6.0, the release of ADT started at 10 min (Fig. 4b and Supplementary Fig. 26), gradually increased with time, and finally stabilized after 72 h.

To verify this view that a low concentration of H_2_S promotes cell growth and a high concentration inhibits cell growth, CCK-8 cytotoxicity experiments were performed. The results showed that in 4T1 cells, when the concentration of H_2_S was less than 230 μM, cell viability increased with H_2_S concentration (Fig. 4c). However, when the concentration of H_2_S was higher than 230 μM, the cell viability decreased. In C26 cells, when the concentration of H_2_S was less than 394 μM, cell viability increased with H_2_S concentration, while when the H_2_S concentration was higher than 394 μM, cell viability decreased (Supplementary Fig. 27). Furthermore, to analyse the effect of H_2_S release from PCoA@M, a qualitative experiment investigating the induced release of tris(2-carboxyethyl)phosphine (TCEP) was performed. TCEP can induce the cleavage of the disulfide bonds in ADT and release H_2_S, which then chemically reacts with lead acetate to form a black lead sulfide precipitate. After 3 h of reaction at room temperature, the lead acetate-impregnated filter paper in the PCoA and PCoA@M groups appeared black in colour (Supplementary Fig. 28) compared to the PDA and PCo groups. This result indicated that the released H_2_S from PCoA and PCoA@M chemically reacted with lead acetate to generate black PbS, which proved that PCoA@M could release H_2_S in a very responsive manner and provided a basis for H_2_S therapy. Washington State Probe-1 (WSP-1) can react with H_2_S selectively and rapidly to generate benzodithiolone and another fluorescent compound (E_x_ = 465 nm, E_m_ = 515 nm). Subsequently, fluorescence detection of H_2_S was performed based on a fluorescence on/off reaction strategy. When H_2_S solution was coincubated with WSP-1, the fluorescence signal of the fluorescent compound increased with incubation time within 2 h (Supplementary Fig. 29) and the intensity remained steady at 4 h, so the optimal time for the reaction was 2~4 h. Next, PCoA@M and WSP-1 were coincubated with 4T1 cells for different lengths of time. With increasing incubation time, the area with green fluorescence expanded continuously (Fig. 4d) with an accompanying increase in intensity that reached a maximum at 4 h due to the reaction of WSP-1. These results were basically consistent with the in vitro fluorescence detection data. Thus, after coincubation of PCoA@M and 4T1 cells, the ADT prodrug released H_2_S and the amount released continued to increase with time, which led to a large amount of the fluorescent product from the reaction between H_2_S and WSP-1. To investigate whether the H_2_S released from the PCoA@M accumulated in the cells to reach a high concentration, the concentration of H_2_S was calculated. When PCoA@M was incubated at pH = 6.0, the amount of released ADT reached 68.5% at 8 h (Fig. 4b and Supplementary Fig. 26); that is, the concentration of released H_2_S was approximately 953.9 μM. There is endogenous H_2_S in 4T1 cells, so after PCoA@M was incubated with the cells, the final concentration of H_2_S was greater than 953.9 μM, which is higher than the lower limit of the H_2_S concentration that inhibits cell growth. To study the growth promotion or inhibition of cells resulting from the effects of H_2_S, 5-ethynyl-2-deoxyuridine (EdU) cell proliferation experiments were conducted. When the concentration of H_2_S was 0, the proliferating cells (green fluorescence) accounted for 41.98% of the total cells (Fig. 4e), while they accounted for 51.89% at 0.75 μM H_2_S, 71.5% at 230 μM H_2_S, and 29.24% at 394 μM H_2_S. The above results further illustrated that low concentrations of H_2_S promoted cell growth and that high concentrations of H_2_S inhibited cell growth. Next, the inhibitory effect of PCoA@M was further verified. After the cells were incubated with PBS and PCoA@M, the proportion of proliferating cells in the PCoA@M group was significantly lower than that in the PBS group (Fig. 4f), which further proved that the H_2_S released by PCoA@M could inhibit the proliferation and growth of 4T1 cells. When cells were cocultured with Dil-labelled PCoA@M for 2 h, the area of red fluorescence in the cytoplasm appeared smaller with weak intensity (Fig. 4g and Supplementary Fig. 30). From 5~6 h, the both the fluorescence area and intensity value reached their maximum levels, indicating that PCoA@M better targeted 4T1 cells.

To further confirm the synergistic therapeutic effect of PCoA@M with different treatments, confocal imaging experiments with calcein-AM and PI cell staining were performed. The results showed that compared with the P@M + NIR group (Fig. 4h), a large amount of red fluorescence appeared in the PCo@M + NIR group, possibly because the degradation of Co-MOF led to the release of a large amount of Co^2+^ and downregulated the expression of HSP, which in turn enhanced the PTT effect and prompted substantial cell apoptosis. In the PCoA@D group, 4T1 cells irradiated with NIR showed only irregular red fluorescence, which further reflected the importance of the macrophage membrane packaging for targeted therapy. Compared to the PCoA@D + NIR and PCo@M + NIR groups, the red fluorescence appeared dense and green fluorescence was weak in the PCoA@M + NIR group, indicating that most of the cells had died. These data further confirmed the synergistic therapeutic effect of targeted photothermal and gas therapy. The good photothermal performance of PCoA@M was then evaluated in vitro (Supplementary Fig. 31). In the RAW 264.7 group, there was no significant apoptosis after treatment with the macrophage membrane-wrapped probe and most of the cells remained bright green, perhaps because the camouflage effect of the macrophage membrane resulted in less phagocytosis of the probe by the cells. However, only a dense red fluorescence signal arose in the PCoA@D + NIR group, indicating that cells in this group had phagocytized a large number of probes, which further confirmed that the probe modified with the macrophage membrane would provide a good escape effect due to camouflage. To quantitatively study the cell apoptosis induced by PCoA@M, FITC-PI flow cytometric analysis of 4T1 cells was performed (Fig. 4i and Supplementary Fig. 32). Only a small amount of early cell apoptosis was observed (1.28%) after treatment with the laser alone. However, the cell necrosis rate in the P@M + NIR group reached 25.3%, that in the PCo@M + NIR group reached 46.7%, and that in the PCoA@M + NIR group reached 73.6%, showing that after the dual treatment of laser combined with gas, most of the cells had undergone necrosis. These results were consistent with those of the confocal imaging experiments, indicating that the improved photothermal and gas starvation synergistic therapy exhibited a powerful effect on inhibiting tumour cell growth.

### In vivo imaging of PCo@M

**Fig. 5:**
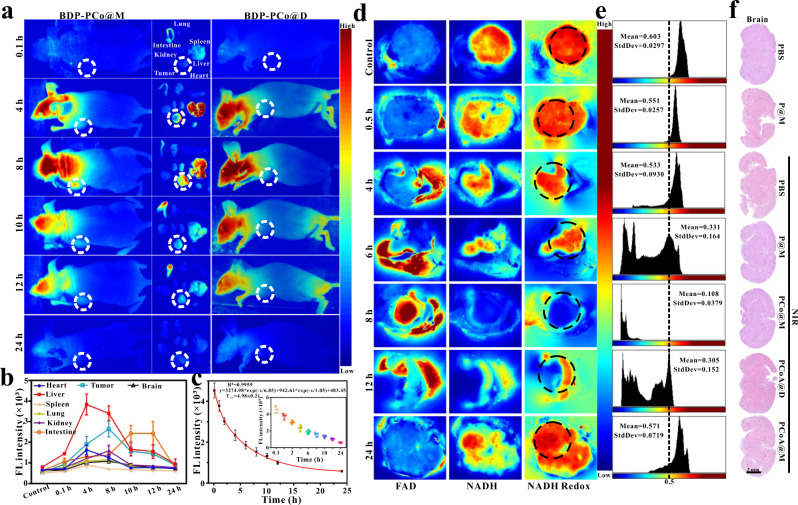
Images of the targeting probes in vivo. **a–c** Fluorescence images of whole mice, main organs, and tumours at different times after injection of different probes into the tail vein of mice with 4T1 tumours in situ. Metabolic analysis of the probes in mouse organs and blood. Colour scale: greyscale images were converted to pseudocolour images, and blue to red represents the fluorescence intensity values of BDP from low to high, FL Fluorescence. Data are presented as the means ± s.d. (*n* = 5 mice per group). **d**, **e** 2D deep-hypothermia fluorescence imaging of NADH and FAD, the redox ratio of NADH/(NADH + FAD), and the corresponding area histogram of 4T1 tumours at different times after injection of PCoA@M into mice. Colour scale: blue to red represents relative fluorescence intensity values of NADH and FAD from low to high. **f** HE staining of brain tissue from 4T1 tumour-bearing mice after different treatments.

Based on the in vitro experimental results, the in vivo application of the probes was carried out. With the good biocompatibility and long-term biosafety (Supplementary Fig. 33a–m) of the probes, their ability to become enriched at the target site was investigated. PCo@M labelled with the fluorescent dye BDP was injected via the tail vein into BALB/c mice inoculated with 4T1 tumours in situ, and fluorescence imaging of the mice was carried out continuously. The results (Fig. 5a) showed that there was a significant fluorescence signal in the liver at 4~8 h and in the small intestine at 10~12 h, indicating the uniform distribution of the probe in mice via blood circulation. Four hours after injection, a fluorescence signal appeared at the tumour site, increasing in intensity with time up to its maximum enrichment at 8 h, followed by a gradual decrease in its signal. The above results were also demonstrated by fluorescence analysis (Fig. 5a, b) of the organs removed at different times. The fluorescence signal of the small intestine reached a maximum at 10~12 h, exhibiting a significant difference from the other time points. The main reason for the increase in fluorescence was undergoes metabolism in and excretion through the small intestine. The fluorescence signal at the tumour site at 8 h was strong compared with that at other times, showing consistency with the overall fluorescence imaging results in mice. The blood half-life of PCo@M was 4.98 ± 0.21 h (Fig. 5c), which laid a foundation for the enrichment of the probe at the tumour site. As a control, the fluorescence signal of BDP-labelled PCo@D changed slightly and was always weaker than that in the PCo@M group at the same time point (Supplementary Fig. 34). All of the fluorescence signals in the organs in these two groups disappeared after 24 h. The aforementioned experiments demonstrated that PCo@M could accurately target the tumour site with abundant blood vessels via blood circulation.

Because the tumour site is hypoxic, tumour cells mainly rely on glycolysis for energy. A large amount of NADH is needed for glycolysis, and ADT can change the relative content of NADH at the tumour site, which in turn affects the NADH/FAD redox balance. Therefore, under deep hypothermia induced by liquid nitrogen, the enhanced autofluorescence intensity of NADH/FAD in tumours was regarded as an evaluation index to determine the concentration of the probe, which was complementary to exogenous fluorescent dye-labelled probe imaging. The 2D/3D results showed that before injection of PCoA@M, the NADH at the tumour site was mostly in its reduced state (Fig. 5d and Supplementary Fig. 35), and its fluorescence intensity remained the same. After injection of PCoA@M, the fluorescence intensity of NADH significantly weakened while the distribution of FAD showed the opposite trend. However, the distribution of the redox ratio was consistent with the amount of NADH, which was also demonstrated by the regional intensity analysis of NADH redox over time (Fig. 5e). At 8 h, NADH and FAD displayed the weakest and strongest fluorescence signals, respectively, and the NADH/(NADH + FAD) value was the lowest recorded. Therefore, based on the results of NADH and FAD imaging at the tumour site, the content of PCoA@M was reconfirmed to have reached a maximum at 8 h. Fluorescence imaging showed clear signals in the brains of mice (Fig. 5a). Therefore, in the different probe treatment groups, HE staining of brain slices was performed. The results demonstrated that the brain tissues of the mice were normal (Fig. 5f), and there were no significant differences compared with the control group, indicating no notable damage to the brain.

### In vivo antitumor and antimetastatic effects of the probe against breast tumours

**Fig. 6:**
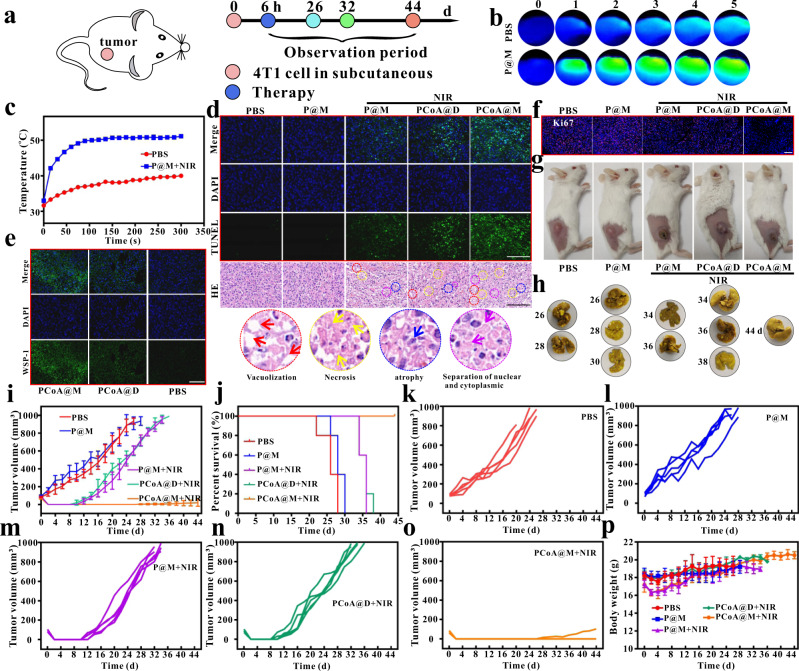
Therapy of subcutaneous tumours. **a** Treatment process. **b** Thermal imaging of tumours after injecting in situ 4T1 tumour-bearing mice with PBS and P@M and treatment with NIR. **c** Temperature change curves over time. Data are presented as the means ± s.d. (*n* = 3 mice per group). **d–f** TUNEL, HE, WSP-1, and Ki-67 immunofluorescence staining images of the tumour site. Images are representative of three biologically independent mice. Scale bar in d: 100 μm; scale bar in **e**: 400 μm; scale bar in **f**: 800 μm. **g**, **h** Bright field images of subcutaneous 4T1 tumour-bearing mice after different treatments and of lung tissue after their sacrifice. **i** Mean tumour volume changes. Data are presented as the means ± s.d. (*n* = 5 mice per group). **j** Survival curves after different treatments. **k**, **l**, **m**, **n**, and **o** Changes in tumour volume in different parallel treatment groups. **p** Weight changes over time. Data are presented as the means ± s.d. (*n* = 5 mice per group).

A tumour model and lung metastasis model were established by subcutaneous (Fig. 6a), orthotopic, and tail vein injection. The subcutaneous injection method formed a solid tumour area by injecting a certain concentration of tumour cells into the backs of mice. When the tumour volume reached approximately 100 mm^3^, different probes were injected. The results showed that P@M possessed excellent thermal conversion efficiency (Fig. 6b, c). TUNEL, HE (Fig. 6d), WSP-1 (Fig. 6e), and Ki-67 (Fig. 6f and Supplementary Fig. 36) staining and bright field images (Fig. 6g) of the mice after immediate treatment demonstrated the targeted therapeutic effect of PCoA@M. The time of death of each mouse was defined when the tumour volume reached around 1000 mm^3^ or the mouse died naturally. By dissecting the mouse organs at the time of sacrifice, few metastatic nodules were found in the lungs of the PBS and P@M mouse groups (Fig. 6h), as these treatments could not lead to the formation of effective lung metastases; additionally, no obvious metastatic nodules appeared in other organs such as the heart, liver, spleen, or kidney (Supplementary Figs. 37–41). The subcutaneous injection method was convenient and easy to perform and the formation rate of tumours in situ was low, so there were many restrictions on modelling. Nevertheless, PCoA@M still possessed a good therapeutic effect on subcutaneous tumours, which was also demonstrated by the tumour volume results (Fig. 6i, k-o) and survival curves (Fig. 6j). Moreover, the probes had little effect on the body weights of mice (Fig. 6p), which further illustrated the biosafety of the probe in vivo.

**Fig. 7:**
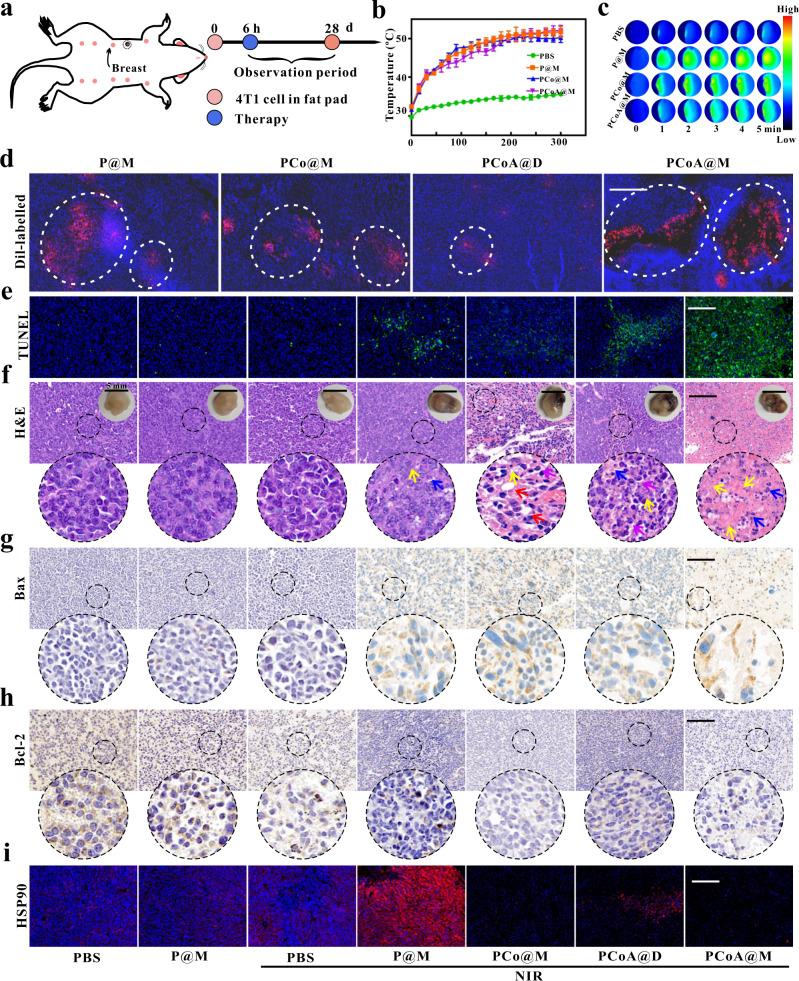
Short-term therapy of in situ tumours. **a** Treatment process. **b**, **c** Temperature change curves over time and thermal images of tumours after injecting of in situ 4T1 tumour-bearing mice with PBS, P@M, PCo@M, and PCoA@M and treated with NIR. Colour scale: black to red represents the temperature of the tumour from low to high. Data are presented as the means ± s.d. (*n* = 3 mice per group). **d** Fluorescence imaging of 4T1 tumour sections after different Dil-labelled probes were injected into mice. Scale bar: 400 μm. **e–i** TUNEL, HE, Bax, Bcl-2, and HSP90 immunofluorescence staining at the tumour site. Red arrow in **f**: vacuolization in the cell; blue arrow: cell necrosis; yellow arrow: cell atrophy; purple arrow: separation of the nucleus and cytoplasm. Images are representative of three biologically independent mice. Scale bars in **e**, **g**, **h** and **i**: 100 μm; scale bar of HE images in **f**: 100 μm; scale bar of tumour images in **f**: 5 mm.

Next, a tumour model and lung metastasis model were established by orthotopic injection, and the immediate effect of orthotopic breast cancer treatment was evaluated (Fig. 7a). The statistical results of tumour site temperature (Fig. 7b, c and Supplementary Fig. 42) revealed that in the PBS + NIR group, the temperature reached only ~34 °C, which is not high enough to kill tumour cells. Treatment with low-intensity NIR (5 min) 8 h after injection caused the temperature to rise ~51.2 °C in the P@M, PCo@M, and PCoA@M groups, indicating that the probes actively targeted the tumour site and produced a significant photothermal effect; this also means that the probe possessed the ability to induce tumour cell apoptosis. Based on the excellent overall fluorescence imaging results mentioned above (Fig. 5a, d), the effect of the probe to target the orthotopic 4T1 tumour site was further verified. Through the tail vein, Dil-labelled P@M, PCo@M, PCoA@D, and PCoA@M were injected into 4T1 tumour-bearing mice via the tail vein. After 6 h, the mice were sacrificed, and then the tumours were removed, embedded in cryoembedding media (OCT), and subsequently stained with DAPI. Mice treated with P@M, PCo@M, and PCoA@M displayed an intense red fluorescence signal at the tumour site (Fig. 7d and Supplementary Fig. 43), while the mice in the PCo@D group showed relatively weak fluorescence. This result indicated that after modification with the macrophage membrane, the probes had a good ability to target 4T1 tumours, thus laying the foundation for evaluating the in vivo therapeutic applications of PCoA@M.

**Fig. 8:**
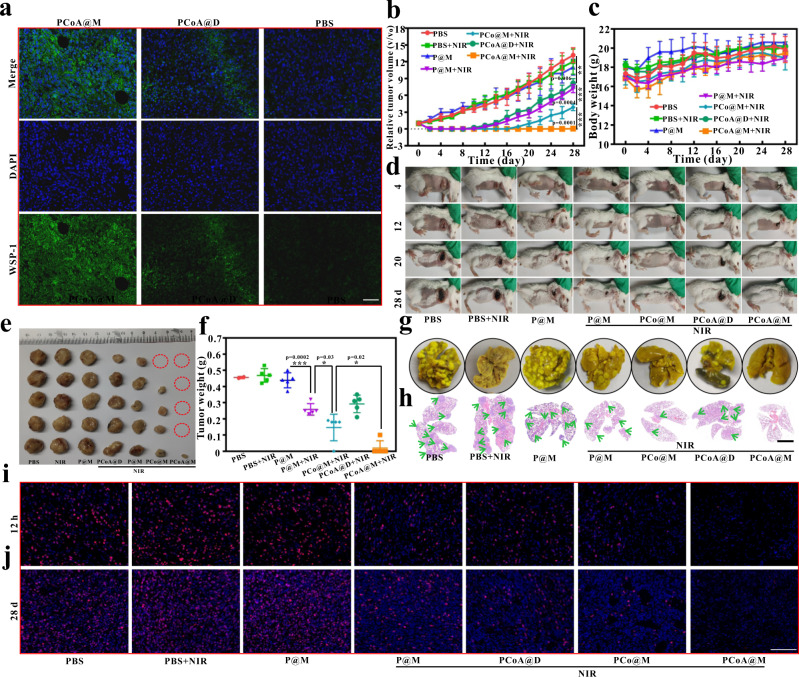
Long-term therapy of in situ tumours. **a** WSP-1 immunofluorescence staining images of the tumour site after different treatments. Images are representative of three biologically independent mice. Scale bar: 200 μm. **b** Relative tumour volume changes. Data are presented as the means ± s.d. (*n* = 5 mice per group). Statistical differences were calculated using two-tailed Student’s *t* test, *: *p* < 0.05; **: *p* < 0.01; ***: *p* < 0.001. **c–f** Mouse weight changes, bright field images of the mice and tumours and tumour quality analysis. Data are presented as the means ± s.d. (*n* = 5 mice per group). Statistical differences were calculated using two-tailed Student’s *t* test, *: *p* < 0.05; **: *p* < 0.01; ***: *p* < 0.001. **g**, **h** Bouin’s trichrome fixed lung tissue of mice after sacrifice and the corresponding HE staining images. Scale bar: 4 mm. **i**, **j** Ki-67 immunofluorescence staining images of the tumour site at different times after different treatments. Images are representative of three biologically independent mice. Scale bar: 100 μm.

From the TUNEL and HE staining images of the tumour sections, the mouse tumour cells in the PBS, PBS + NIR, and P@M groups grew normally (Fig. 7e, f and Supplementary Fig. 44), and no notable cell necrosis appeared. In comparison, the cells in the P@M + NIR and PCo@M + NIR groups displayed different degrees of vacuolization, cell necrosis, atrophy, and separation of the nucleus and cytoplasm, which verified that both single and dual PTT had a certain effect during the initial stage of therapy. A certain degree of apoptosis was observed in the PCoA@D group because a small amount of PCoA@D became passively enriched at the tumour site, resulting in enhanced photothermal and gas-starvation therapy. The staining results also revealed obvious cell necrosis in the PCoA@M + NIR group, which further illustrated the powerful effect of the enhanced targeted photothermal synergistic gas-starvation therapy. To clarify whether cell killing would change the expression of Bax and Bcl-2, immunofluorescence staining of tumours was performed. Compared with the other groups, a stronger brown signal from Bax staining was observed in the PCoA@M + NIR group (Fig. 7g) whereas a weaker brown signal was observed for Bcl-2 staining (Fig. 7h). These data showed that PCoA@M tended to increase the expression of the apoptotic protein Bax and decrease that of the anti-apoptotic protein Bcl-2, thereby promoting apoptosis and further proving the effects of enhanced photothermal synergistic gas-starvation therapy at the tissue level. Compared with the PBS, PBS + NIR, and P@M groups, HSP expression in the P@M + NIR group was significantly increased due to NIR treatment (Fig. 7i), while it was markedly reduced in the PCoA@D + NIR, PCo@M + NIR, and PCoA@M + NIR groups, perhaps because the degradation of Co-MOF led to the inhibition of HSP90 expression from free Co^2+^. Additionally, the H_2_S release from PCoA@M was verified by WSP-1 experiments (Fig. 8a), which further laid the foundation for investigating targeted orthotopic breast cancer therapy.

Based on the above immediate treatment effects, a long-term in vivo study was carried out. From the fluorescence imaging results (Fig. 5a, b), it was clear that at the tumour site, the probe was maximally enriched 4–8 h after injection; therefore, the 6th hour after the probe injection was chosen for the synergistic treatment study. 28 d later, the mice were sacrificed, and the tumour volume in each group was measured as PBS (1050.00 mm^3^, 1102.99 mm^3^, 1086.90 mm^3^, 1135.81 mm^3^, and 1069.67 mm^3^), PBS + NIR (1127.79 mm^3^, 1027.76 mm^3^, 1180.90 mm^3^, 998.65 mm^3^, and 1015.69 mm^3^), P@M (1140.25 mm^3^, 1017.33 mm^3^, 915.26 mm^3^, 888.08 mm^3^, and 1126.50 mm^3^), P@M + NIR (654.39 mm^3^, 709.27 mm^3^, 619.27 mm^3^, 714.10 mm^3^, and 680.63 mm^3^), PCo@M + NIR (345.94 mm^3^, 350.40 mm^3^, 0, 345.07 mm^3^, and 340.40 mm^3^), PCoA@D + NIR (651.08 mm^3^, 611.16 mm^3^, 632.92 mm^3^, 612.91 mm^3^, and 715.08 mm^3^), PCoA@M + NIR (0, 18 mm^3^, 0, 0, and 0). The therapeutic effect could be roughly divided into the following four categories according to the size of the tumour (Fig. 8b). (I) PBS, P@M, and PBS + NIR groups: the tumour changes in these three groups were similar, and the tumour volume continued to increase with time, indicating that P@M treatment or laser irradiation alone had almost no therapeutic effect on the tumours. (II) P@M + NIR and PCoA@D + NIR groups: compared with category I, the tumour growth in these two groups was significantly inhibited for 10–12 d, but afterwards, the tumours continued to grow and showed a similar change in volume, which was significantly different from category I (*p* < 0.01). The difference between P@M + NIR and PCoA@D + NIR lies in targeted PTT versus passive enhanced photothermal synergistic gas-starvation therapy. These data illustrated that targeting could compensate for the limitations of a single treatment modality, and dual-modal therapy could offset the lack of targeting to a certain extent. (III) PCo@M + NIR group: after treatment with NIR, tumour growth was significantly inhibited for 16 d but then continued to grow. The growth rate was significantly slower than that in category II (*p* < 0.001), indicating that at the initial stage, the targeted probe was enriched at the tumour site and had a marked inhibitory effect on the tumour after enhanced PTT. Nevertheless, the tumour could still not be thoroughly cured. (IV) PCoA@M + NIR group: after enrichment of the targeted probe at the tumour site, the enhanced photothermal and gas-starvation therapy induced by NIR was very obvious, and all of the tumours disappeared. No signs of tumour recurrence appeared until 28 d later, and the tumour sizes differed significantly from those in category III (*p* < 0.001). These data show that PCoA@M fully exerted the improved photothermal coupled gas-starvation therapy effect. Body weight measurements of the mice after 2 d of treatment revealed a decrease in each group (Fig. 8c). A possible reason for this decrease was that the anaesthetics caused the mice to have a poor appetite. After 4 d of treatment, the body weights of the mice in each group recovered, indicating that the probe was nontoxic to the mice. After 28 d of observation, the bright field images of the mice (Fig. 8d) and ex vivo tumours (Fig. 8e) illustrated the excellent long-term treatment effects of the probe. Tumour quality analysis in the different experimental groups demonstrated an excellent synergetic treatment effect (Fig. 8f). Hence, the therapeutic effect of gas starvation in combination with enhanced PTT displayed a significant therapeutic improvement compared with that of simple or enhanced PTT. Under the premise of the low in vivo toxicity, a good enhanced photothermal gas starvation combined therapy effect was achieved by PCoA@M. Here, the characteristics of low toxicity, mild laser irradiation, and high efficacy provides ideas for the design of a tumour treatment strategy. To investigate whether the probe had an effect against lung metastasis, the lung tissues from the mice in the different groups were removed, and the metastatic nodules were counted after fixation with Bouin’s trichrome. Because lung nodules reflect tumour metastasis, the severity of tumour metastasis can be directly obtained from the number of tumour lung nodules. The results showed that in the PBS, P@M, PBS + NIR, P@M + NIR, PCoA@D + NIR, PCo@M + NIR, and PCoA@M + NIR groups, the numbers of tumour nodules were 32.4, 34.2, 30, 18.6, 17.8, 10.4, and 2, respectively (Fig. 8g and Supplementary Fig. 45). HE staining data revealed that the tumour metastatic areas were in the following order: PBS ≈ P@M ≈ PBS + NIR > P@M + NIR ≈ PCoA@D + NIR > PCo@M + NIR > PCoA@M + NIR (Fig. 8h), which was similar to the nodule count results. Additionally, the immediate and long-term therapeutic effects (Fig. 8i–j and Supplementary Figs. 46–47) of the different treatments were verified by Ki-67 immunofluorescence staining of the orthotopic tumour sites. These findings demonstrated the specific antimetastatic effect of PCoA@M and its practicability for advanced tumour treatment.

**Fig. 9:**
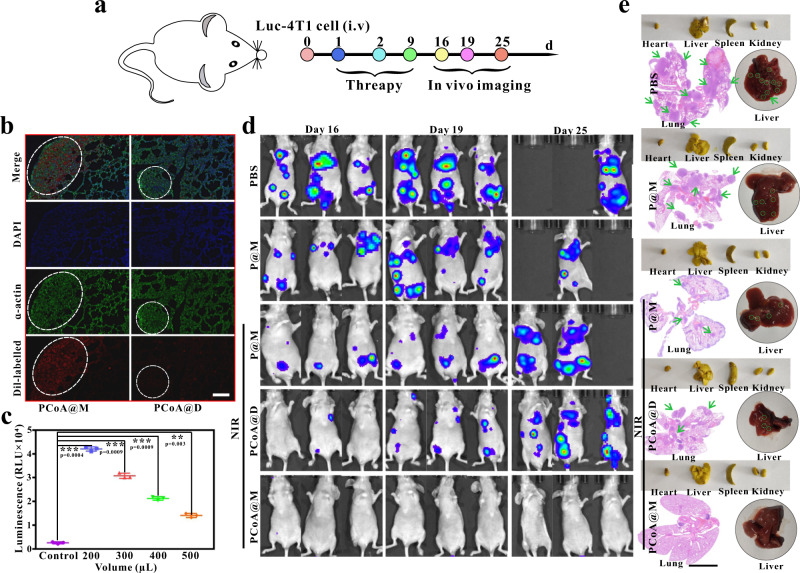
Metastatic tumour therapy. **a** Metastatic tumour model treatment process via injection of luc-4T1 cells into the tail vein. **b** α-Actin immunofluorescence imaging of lung tissues in mice with metastatic tumours after treatment with different Dil-labelled probes. Images are representative of three biologically independent mice. Scale bar: 200 μm. **c** Luminescence intensity of the luciferin reporter gene after luc-4T1 cells were incubated with different volumes of lysate. Data are presented as the means ± s.d. (*n* = 3 mice per group). Statistical differences were calculated using two-tailed Student’s *t* test, *: *p* < 0.05; **: *p* < 0.01; ***: *p* < 0.001. **d** Bioluminescence imaging at different times after the injection of different probes into mice bearing 4T1 lung metastases. **e** Bright field images of main organs fixed with Bouin’s trichrome (yellow) and original liver and HE staining of the corresponding lung tissues. Scale bar: 4 mm.

To further verify the treatment effect of the probe against breast cancer lung metastasis, a lung metastasis model was established by tail vein injection (Fig. 9a) and an in vivo lung targeting study was performed. Based on the fibrosis of lung tissue, α-actin immunofluorescence staining of lung tissue was carried out. Through localization of its cytoskeleton, the metastatic tumour area in the lung tissue could be more clearly displayed. The results showed that in the metastatic area, the cytoskeleton was denser (Fig. 9b) while the normal tissues showed fibrosis. After tail vein injection of Dil-labelled PCoA@M and PCoA@D, the metastatic tumour area in the lungs of the mice in the PCoA@M group had a significant red fluorescence signal, but this signal was hardly present in the PCoA@D group, which further demonstrated the targeting of our probe for lung metastatic tumours. In 4T1 cells transfected with luciferin, the luciferase reporter gene was detected. At the cellular level, bioluminescence can quantitatively detected. The relative light unit (RLU) values showed significant differences from the control group with the addition of 200 (50%), 300 (33%), 400 (25%), and 500 μL (20%) of cell lysate, indicating that luc-4T1 cells presented better expression of the luciferin reporter gene (Fig. 9c). After constructing a lung tissue metastasis model with 4T1 cells transfected with the luciferase reporter gene, the bioluminescence of the lung tissue was recorded to analyse the antimetastatic effects of different probes. The results showed an increase in the lung bioluminescence signals in the PBS, P@M, P@M + NIR, and PCoA@D + NIR groups over time (Fig. 9d). Additionally, the P@M + NIR and PCoA@D + NIR groups had relatively weaker signals than the PBS and P@M groups. On the 25th day, there were 2 dead mice in each the PBS and P@M groups, possibly because the metastasis of the tumour cells was too serious and caused restricted growth. In the PCoA@M + NIR group, almost no bioluminescence signal was detected in the lungs. On the 25th day, the growth status of the mice was determined to be good, indicating the excellent antimetastatic effect of PCoA@M. After the experiment, the lung tissues of the surviving mice were removed to count the number of metastatic tumour nodules. In the PBS, P@M, P@M + NIR, PCoA@D + NIR, and PCoA@M + NIR groups, the numbers of nodules were approximately 33.2, 31.6, 16.4, 17.3, and 4.5, respectively. The lung tissue of one mouse in each group was also fixed with Bouin’s trichrome, embedded, sliced, and stained with HE. In the PBS, P@M, P@M + NIR, and PCoA@D + NIR groups, there were many 4T1 tumour metastasis areas (Fig. 9e), while the PCoA@M group showed fewer areas. All of the aforementioned results further proved that PCoA@M had an excellent therapeutic effect against metastatic tumours. Compared with orthotopic injection, tail vein injection has a faster success rate of creating lung metastases, and tumour metastasis in other organs can also be achieved (Fig. 9e). This may be because direct injection of tumour cells into the tail vein causes the tumour cells to fully form implants in the organs after a long time in blood circulation. Therefore, this method is helpful for the exploration of metastatic tumour models, such as lung, liver, and other organ metastases, and it is also a means to study drug treatment of metastasis.

## Discussion

In this study, a biomimetic nanoplatform based on the macrophage membrane was developed. A core-shell PDA@Co-MOF heterostructure loaded with a H_2_S prodrug and wrapped with a macrophage membrane was fabricated by a facile one-pot synthesis. The nanoplatform had a vascular targeting ability and could become enriched in the tumour microenvironment. Currently, most HSP inhibitors in the clinic are small molecules, and nanomaterial-based inhibitors have rarely been reported. However, some metal ions have been found to be able to bind to HSP. Here, it was found that after the degradation of Co-MOF in the acidic tumour microenvironment, the free Co^2+^ downregulated the expression of HSP90, thereby inhibiting the heat resistance of HSP and enhancing the effects of PTT. Moreover, a high concentration of H_2_S was released from the prodrug to kill tumours. ADT also significantly changed the NADH content at the tumour site and affected the NADH/FAD balance. Furthermore, after a large amount of NADH was consumed, ATP synthesis was restricted in the system. Therefore, the energy supply to the cell slowed down, which in turn caused a certain degree of tumour growth inhibition by starvation therapy. Deep hypothermia was exploited to enhance the autofluorescence imaging of NADH/FAD in vivo, which is also considered an important indicator for judging the enrichment of the probe at the tumour site. The results showed that for in situ 4T1 tumours, this nanoplatform could achieve the targeted enhancement of photothermal synergistic gas-starvation therapy and inhibit lung metastasis. For breast cancer, the commonly used first-line treatment is a combination of CDK4/6 inhibitors, such as palbociclib, with endocrine drugs. Clinical studies have shown that the progression-free survival time of patients undergoing palbociclib combination therapy was 27.6 months. For second- and later-line treatments, patients who progress after first-line endocrine therapy but have not received CDK4/6 inhibitor therapy can choose CDK4/6 inhibitors, such as palbociclib, combined with fulvestrant or an aromatase inhibitor. Here, the progression-free survival time of patients treated with palbociclib and fulvestrant was 46.7 months. According to the equivalent ages of humans and rats (a 12-month-old rat is equal to a thirty-year-old human), first-line combination therapy can prolong the survival time of rats by 27.6 d, and second-line and later-line treatments can prolong the rat survival by 46.7 d. Based on the rate of tumour recurrence (Fig. 6i, o), we calculated that our probe (PCoA@M) would require at least 26 d for a recurrent mouse tumour to reach a volume of 1000 mm^3^. Thus, the survival time of mice treated with PCoA@M + NIR could be extended by at least 44 d, which is superior to the first-line combination drug treatment for clinical breast cancer in the clinic. Therefore, it is expected that our developed nanoplatform could provide an opportunity for tumour treatment and tumour metastasis inhibition.

## Methods

### Materials

Trichloromethane, triethylamine, and methanol were purchased from Sinopharm Chemical Reagent Co., Ltd. Cobalt nitrate hexahydrate (Co(NO_3_)_2_ · 6H_2_O), dopamine, and 2-methylimidazole were purchased from Aladdin. Anethole trithione was obtained from TargetMol. A membrane and cytosol protein extraction kit, Dil, LysoTracker Green, and a firefly luciferase reporter gene detection kit were obtained from Shanghai Beyotime Biotechnology Co., Ltd. D-Luciferin and potassium salt were purchased from Shanghai Yisheng Biotechnology Co., Ltd. A cell counting kit-8 (CCK-8) was purchased from Nanjing Novazan Biotechnology Co., Ltd. Annexin V and an FITC apoptosis detection kit were obtained from Dojindo Laboratories. Anti-integrin alpha 4 rabbit pAb, anti-integrin beta 1 rabbit pAb, Cy3-labelled goat anti-rabbit IgG, FITC-labelled goat anti-rabbit IgG, and luc-4T1 cells were obtained from Wuhan Service Biotechnology Co., Ltd. A Pierce BCA protein assay kit was purchased from Thermo Fisher Scientific. Bouin’s fixative was purchased from Jinclone Biotechnology Co., Ltd. (Beijing). Distearoylphosphatidylethanolamine-polyethylene glycol 2000 (DSPE-mPEG_2000_) was obtained from Avanti. All reagents were analytically pure and used directly after purchase without further purification.

### Cell lines and animals

The 4T1 and C26 cell lines were obtained from the China Centre for Type Culture Collection (Wuhan, China), the luc-4T1 cell line was obtained from Wuhan Service Biotechnology Co., Ltd., and the RAW 264.7 cell line was kindly provided by Dr. Deqiang Deng (Britton Chance Centre for Biomedical Photonics at Wuhan National Laboratory for Optoelectronics). Each cell line was morphologically confirmed according to the information provided by the cell-source centre, and the main 4T1 and RAW 264.7 cell line were authenticated with short tandem repeat (STR) analysis (Supplementary Table 1 and 2). All cells were cultured in DMEM containing 10% FBS, 100 U mL^−1^ penicillin and 100 U mL^−1^ streptomycin at 37 °C in 5% CO_2_ humidified air. Four-week-old female BALB/c nude mice (SPF grade) and 4-week-old female BALB/c white mice (SPF grade) were purchased from Beijing Weitong Lihua Biotechnology Co., Ltd. Mice were housed in an animal facility under constant environmental conditions (room temperature, 22 ± 1 °C; relative humidity, 40-70%; and a 12 h light-dark cycle), and all mice had access to food and water ad libitum. All animal experiments were approved by the Animal Experiment Ethics Committee of Huazhong University of Science and Technology (IACUC Number: S904). The tumour volume was calculated as 0.5×(length×width×width). To minimise animal discomfort, according to the Guideline of Assessment for Humane Endpoints in Animal Experiment (Certification and Accreditation Administration of the P. R. China, RB/T 173-2018), in general experiments, the tumour burden should not exceed 5% of the animal’s normal body weight; in therapeutic experiments, it should not exceed 10% of the animal’s body weight (10% indicated that the diameter of the subcutaneous tumour on the back of a 25 g mouse reached 17 mm). At the end point of the mouse experiment, mice were euthanized to comply with animal welfare standards (euthanasia of all the animals was performed with isoflurane in small animal anaesthetics).

### One-step synthesis of PDA@Co-MOF/ADT (PCoA)

One millilitre of Co(NO_3_)_2_·6H_2_O (0.08 mM) was mixed with 19 mL of methanol containing PDA (2 mg), and after full stirring, 5 mL of dimethylimidazole (0.7 mM), 5 μL of triethylamine, and 1 mL of ADT (8 mM) were quickly added. After stirring evenly at room temperature for 3.5 h and centrifuging at 15286 g for 15 min, the precipitate was collected after washing with methanol several times.

### Extraction of RAW 264.7 macrophage membranes and determination of membrane protein concentration

After approximately 1.2 × 10^8^ RAW 264.7 macrophages were washed with PBS, the cells were digested with EDTA and centrifuged (200× *g*, 6 min) at 4 °C. After removing the supernatant, 3 mL of membrane protein extraction reagent A consisting of PMSF (30 μL) was added to the cell pellet. After fully mixing, the cells were placed in an ice bath for 15 min, repeatedly frozen in liquid nitrogen (3 min) and thawed at 37 °C (6 min) 3 times. The nuclei and unbroken cells were removed by centrifugation (700 × *g*, 10 min) at 4 °C, and the pellet was collected and dissolved in PBS. Finally, cell membrane debris was collected by centrifugation (14000 × *g*, 30 min) at 4 °C and freeze-dried into a powder for later use.

The albumin standard solution was diluted to 0, 0.31, 0.625, 0.125, 0.25, 0.5, 1, and 2 mg mL^−1^. Twenty microlitres of each sample (four parallel groups) was inoculated in a 96-well plate, and 200 μL of BCA working solution was subsequently added to each well. After incubation at 37 °C for 30 min, the absorbance value of each well was measured at 560 nm with a microplate reader, and the protein concentration of each sample was calculated according to the standard curve.

### Synthesis of PDA@Co-MOF/ADT@membrane (PCoA@M)

One millilitre of PCoA (0.5 mg mL^−1^) and 1 mL of RAW 264.7 cell membrane fragment (1.6 mg mL^−1^) were mixed and stirred in an ice bath for 1 h and then extruded repeatedly through an Avanti micro extruder (400 nm polycarbonate porous membrane). After centrifuging to remove the excess cell membrane fragments, RAW 264.7 cell membrane-encapsulated PCoA@M was obtained. Using the aforementioned method, the fluorescent dye BDP-labelled PDA@Co-MOF-BDP@M (PCoB@M), PDA@Co-MOF-ADT@Lipidsome (PCoA@D) wrapped with phospholipids instead of the cell membrane and cell membrane-wrapped PDA (P@M) and PCo (PCo@M) were synthesized as controls.

### Cell-targeting, colocalization and phagocytosis of the probes

Approximately 5 × 10^5^ 4T1 cells were inoculated in confocal culture dishes and cultured in a 5% CO_2_ incubator at 37 °C for 12 h. After removing the medium, 10 μL of 1,1’-dioctadecyl-3,3,3’,3’-tetramethylindocarbocyanine perchlorate (Dil)-labelled PCo@M (Dil: 1 μg mL^−1^), LysoTracker Green, and Hoechst 33342 were added. After thorough rinsing with PBS, the culture dishes were observed under a confocal microscope.

Under the same culture conditions, 4T1 and RAW 264.7 cells were seeded in confocal culture dishes. For the 4T1 cell group, 1 mL of serum-free medium containing Dil-labelled PCo@M (Dil: 1 μg mL^−1^) was added at 4, 5, and 6 h. For the RAW 264.7 cell group, the same concentrations of Dil-labelled PCo@M and PCo@H were added at 4 h. After washing thoroughly with PBS and fixing with 1 mL of 4% paraformaldehyde at room temperature for 20 min, 100 μL of DAPI was added to stain the cells for 10 min. After complete rinsing with PBS, the cells were observed under a confocal microscope.

### SDS–PAGE of the probe and western blot analysis of HSP90 after cells underwent different treatments

A 10% separation gel and 5% concentrated gel solution were prepared in a glass plate at room temperature until the gel solidified. Membrane lysis solution (PMSF) was used to fully lyse the appropriate amounts of PCoA@M and PCoA@D. Then, 5 μL of Coomassie brilliant blue solution was mixed with 20 μL of PCoA@M, PCoA@D, and cell membrane debris solution (1 mg mL^−1^). These mixtures were kept in boiling water for 15 min and loaded into wells containing 20 μL of protein marker and the cell membrane debris solution. Next, electrophoresis, staining, and decolorization were conducted consecutively.

Approximately 5 × 10^5^ 4T1 cells were inoculated in a 6-well plate for culture in a 5% CO_2_ incubator at 37 °C for 12 h. After discarding the medium, P@M and PCo@M were added and treated with or without the corresponding laser irradiation. Subsequently, the protein extracts were added separately, and the cells were lysed and collected. Proteins were separated by 10% sodium dodecyl sulfonate polyacrylic gel electrophoresis (SDS–PAGE) and then electrotransferred to PVDF membranes. To prevent the nonspecific binding of antibodies, the membranes were blocked in 5% skimmed milk (prepared with TBST) at 37 °C for 30 min. Cells were incubated with the primary antibody (Service Bio, anti-HSP90 rabbit pAb, P07900, P82995, and P07901 diluted to 1:1000; Service Bio, anti-HSP60 rabbit pAb, P10809, P63039, and P63038 diluted to 1:500), washed 3 times with TBST overnight at 4 °C, and kept at room temperature for another 30 min. Actin was used as a control. The film was scanned and archived, and the optical density value of the target zone was analysed by the Alpha EaseFC 4.0 software processing system.

### Fluorescence detection of α4 and β1 integrins on the surface of RAW 264.7 cells and detection of the fluorescein reporter gene in luc-4T1 cells

Approximately 5 × 10^5^ 4T1 cells were inoculated in confocal dishes for culture in a 5% CO_2_ incubator (37 °C, 12 h). After discarding the medium, the cells were treated with a specific primary antibody (anti-integrin α4 rabbit pAb: Service Bio, p13612, diluted to 1:1000; anti-integrin β1 rabbit pAb: Service Bio, P09055, P49134, diluted to 1:1000) at 37 °C for 25 min, rinsed with PBS at room temperature (three times, 5 min each time), and incubated with FITC- and Cy3-labelled secondary antibodies (Service Bio, diluted to 1:200) (37 °C, 25 min). Following another rinse with PBS, Hoechst 33342 staining solution (37 °C, 10 min) was added for confocal imaging.

An appropriate number of luc-4T1 cells was inoculated in a six-well plate, which was cultured in a 5% CO_2_ incubator (37 °C, 12 h), and the medium was then aspirated. Next, 200, 300, 400, and 500 μL of reporter gene cell lysate was added. After fully lysing and centrifugation (13000 g, 5 min), 100 μL of the supernatant from each sample was mixed with 100 μL of luciferase detection reagent. After evenly pipetting, the luminescence intensity at 560 nm was measured with a microplate reader.

### Cell therapy with the probe

4T1 cells in the logarithmic growth phase were seeded in a 96-well plate for overnight culture in a 5% CO_2_ incubator (37 °C). After the culture medium was removed, 200 μL of serum-free medium containing different concentrations of PCoA@M, PCoA@D, PCo@M, and P@M was added. Six parallel wells were set up for each group. After 5 h and the addition of fresh medium, the cells were administered the corresponding treatment and incubated for 24 h. Then, 20 μL of CCK-8 was added to each well with fresh serum-free medium. After another 4 h of culture, the absorption at 450 nm was measured with a microplate reader.

Under the same conditions, 4T1 cells were inoculated in confocal dishes, and 1 mL of serum-free medium containing different concentrations of PCoA@M, PCoA@D, PCo@M, and P@M was added. After 5 h of incubation and washing 3 times with PBS, the cells were irradiated with a laser under the same conditions (1.0 W cm^−2^), successively stained with calcein and PI for 5 min each, and then observed under a fluorescence microscope after washing 3 times with PBS. Cells treated under the same conditions were sequentially added to Annexin V-FITC/PI apoptosis staining solution. After 15 min, the staining solution was removed, and the cells were washed with PBS 3 times. Apoptosis analysis was performed by flow cytometry.

### In vivo fluorescence and endogenous deep-hypothermia fluorescence in the tumour site

Five-week-old female BALB/c nude mice (SPF grade) were inoculated with approximately 1 × 10^6^ 4T1 cells under the second pair of left breast pads. After 14 d, 4 mice were injected with 200 μL of PCoB@M and PCoB@D (2 mg mL^−1^) through the tail vein and then imaged and observed with a fluorescence imaging system at different time points (0.1, 4, 8, 10, 12, and 24 h). A total of 21 tumour-bearing nude mice were randomly divided into 7 groups, and 200 μL of PCoA@M (2 mg mL^−1^) was injected into their bodies through the tail vein. The mice were euthanized at 0, 0.5, 4, 6, 8, 12, and 24 h. The tumours were quickly removed and frozen in liquid nitrogen. Next, imaging was performed with an NADH/FAD deep-hypothermia fluorescence detection system^52,53^ built in the laboratory.

### In vivo treatment of in situ breast cancer and inhibition of lung metastasis

In the second pair of left breast pads of 5-week-old female BALB/c mice, approximately 1 × 10^6^ 4T1 cells were inoculated in situ. When the tumour volume reached approximately 100 mm^3^, the mice were randomly divided into 7 groups and intravenously treated with (I) PBS, (II) PBS + NIR, (III) P@M, (IV) P@M + NIR, (V) PCo@M + NIR, (VI) PCoA@D + NIR, or (VII) PCoA@M + NIR. The administered volume of all of the aforementioned probes was 200 μL. After treatment, the mice were kept at a constant temperature for 1 h and then sacrificed. The tumours and brains were washed several times, immersed in 4% paraformaldehyde, fixed for 48 h and embedded to make sections. TUNEL, HE, Bax and Bcl-2 staining were performed on the tumour tissues. The brain tissues were stained with HE. Finally, the results were observed under a microscope.

A total of 35 female BALB/c mice (5 weeks old) inoculated with 4T1 cells in situ were divided into 7 groups, and after injection and treatment as described above, the weights of each mouse and their tumour volumes were recorded with a weight scale and a digital vernier calliper every day. Twenty-eight days later, the mice were sacrificed, and the lung tissues were fixed with Bouin’s trichrome and photographed. In each group, the number of metastatic nodules was visually counted, and HE staining was performed after embedding the slices. The results were observed under a microscope. In addition, the tumours were weighed, and the tumour index was determined for every mouse.

A total of 12 mice bearing 4T1 orthotopic tumours (BALB/c, female, 5 weeks old) were randomly divided into 4 groups, and Dil-labelled (I) P@M, (II) PCo@M, (III) PCoA@D, and (IV) PCoA@M were injected through the tail vein. Six hours later, the mice were sacrificed, and the corresponding tumours and lung tissues were removed, fixed with 4% paraformaldehyde, embedded in the sections, and observed under a microscope.

To build a breast cancer lung metastasis model, a total of 15 female BALB/c nude mice were divided into 5 groups and intravenously injected with 2 × 10^6^ luc-4T1 cells. On Days 1, 2, and 9 after injection, the mice were intravenously treated with (I) PBS, (II) P@M, (III) P@M + NIR, (IV) PCoA@D + NIR, or (V) PCoA@M + NIR; afterwards, changes in body weight were regularly monitored and recorded. Furthermore, on Days 16, 19, and 25, fluorescein sodium (15 mg kg^−1^) was intraperitoneally injected into the 4T1 tumour-bearing nude mice. After 15 min, the bioluminescence signal was measured with an IVIS Lumina LT, and the bioluminescence images of the lung tumours were analysed. Next, the mice were sacrificed, and the corresponding lung tissues were fixed with Bouin’s trichrome for HE staining.

### Statistical analysis

Experiments were performed with at least three replicates. All values are presented as the means ± s.d. Statistical analysis was performed with Origin Pro (ver. 8.00.000) and GraphPad Prism (ver. 7.00). Comparisons between two groups were performed using two-tailed Student’s *t* test. Values with *P* < 0.05 were considered significant. ImageJ (ver. 1.4.3.67) and MATLAB R2017a (ver. 1.0.0.1) were used to analyse the fluorescence greyscale. Alpha EaseFC was used to analyse the WB results. Flow cytometry data were analysed with CytExpert (ver. 2.3.0.84). 2D/3D autofluorescence imaging of NADH/FAD was analysed with Imaris x64 (ver. 9.0.1). Confocal images were collected with a FluoView 31 S (ver. 2.3.1.163).

### Reporting summary

Further information on research design is available in the Nature Research Reporting Summary linked to this article.

## Supplementary information

Peer review file

Supplementary Information

Reporting Summary

## Data Availability

The authors declare that all data supporting the findings of this study are available within the article and its Supplementary Information or Source Data file. Source data are provided with this paper.

## Source data

Source Data - updated
