# Supplementary material for: Biomimetic material degradation for synergistic enhanced therapy by regulating endogenous energy metabolism imaging under hypothermia
Source: Nat Commun. 2022 Aug 5;13:4567. doi: 10.1038/s41467-022-32349-2 (PMC9355994; doi:10.1038/s41467-022-32349-2)
Supplement: Supplementary file 2 — Supplementary Information [file 41467_2022_32349_MOESM2_ESM.pdf]

Biomimetic Material Degradation for Synergistic Enhanced Therapy by Regulating Endogenous Energy Metabolism Imaging under Hypothermia

Kai Cheng [1,3], Bo Liu [1,3], Xiao-Shuai Zhang [1,3], Ruo-Yun Zhang [1,3], Fang Zhang [1], Ghazal Ashraf [1], Guo-Qing Fan [1], Ming-Yu Tian [1], Xing Sun [1], Jing Yuan [1, 2]✉ and Yuan-Di Zhao [1, 2]✉

[1] Britton Chance Center for Biomedical Photonics at Wuhan National Laboratory for Optoelectronics-Hubei Bioinformatics & Molecular Imaging Key Laboratory, Department of Biomedical Engineering, College of Life Science and Technology, Huazhong University of Science and Technology, Wuhan 430074, Hubei, P. R. China. [2] Key Laboratory of Biomedical Photonics (HUST), Ministry of Education, Huazhong University of Science and Technology, Wuhan 430074, Hubei, P. R. China. [3] These authors contributed equally: Kai Cheng, Bo Liu, Xiao-Shuai Zhang, Ruo-Yun Zhang. Correspondence and requests for materials should be addressed to J.Y. (yuanj@mail.hust.edu.cn) or to Y.-D.Z. (zydi@mail.hust.edu.cn).

Additional experimental section

Instruments. Fluorescence imaging system was constructed by our laboratory; x'pert3 power X-ray diffractometer (Panalytical BV, Netherlands) was used for the structure analysis of nanoparticle; quantitative analysis of probe-induced apoptosis and antigen expression of cell surface were obtained through Cyto FLEX flow cytometer (Beckman Coulter, USA); FV3000 confocal microscope (Olympus, Japan) was to acquire cell fluorescence staining imaging; changes of characteristic functional groups during synthesis were verified by Vertex 70 infrared spectrometer (Bruker, Germany); absorbance detection of well plate was carried out by flexStation3 microplate reader (Molecular Devices, USA); vacuum concentrator (Eppendorf, Germany) was to purify the probe; MDL-III-808-2.5 W laser (Changchun New Industries Optoelectronics Tech. Co., Ltd, China) was used for laser irradiation; absorption spectrum of probe was proceeded by UV-2550 ultraviolet-visible spectrophotometer (Shimadzu, Japan); electron microscopy characterization was performed on 120 kV HT7700 transmission electron microscope (Hitachi, Japan); particle size and zeta potential of the probe were characterized by Nano-ZS90 nanometer (Malvern, UK).

Synthesis of PDA and Determination of its Mass Extinction Coefficient. According to the literature[1], 90 mL of dopamine hydrochloride (0.95 mmoL) was added in a 250 mL round bottom flask at 50 °C, followed by adding 0.79 mL of NaOH solution (1 mol L^{-1}) under vigorous stirring. When color of the solution changed from colorless to light yellow, and to dark brown, the reaction system was aged for 5 h and centrifuged at 11000 rpm for 15 min. After that, the supernatant was discarded and the precipitate was washed with anhydrous methanol until the supernatant was colorless and transparent. After centrifugation at 4000 rpm for 5 min, the supernatant was collected and dissolved in 16 mL of anhydrous methanol for later use.

PDA was weighed and dispersed in anhydrous methanol to prepare mother liquor (2 mg mL^{-1}), which subsequently was diluted to 60, 45, 30, 22.5, 15, and 7.5 μg mL^{-1} of PDA. With measuring the absorption spectrum of PDA, absorbance value at 280 nm was recorded. A scatter plot was drawed with the concentration as the abscissa and absorbance as the ordinate, then fitting the straight line to obtain the standard curve equation and correlation coefficient (R^2) of PDA.

Synthesis of PDA@Co-MOF (PCo). 1 mL of $Co(NO_3)_2{\cdot}6H_2O$ (0.08 mM) was mixed with 19 mL of methanol containing PDA (2 mg), and after fully stirring, quickly adding 5 mL of

dimethylimidazole (0.7 mM) and 5 μL of triethylamine. With stirred evenly at room temperature for 3.5 h and centrifuged at 11000 rpm for 15 min, the precipitate was collected after washing with methanol for several times.

Determination of the mass extinction coefficient of ADT. 2 mg of ADT powder was dissolved in anhydrous methanol to prepare a mother liquor (2 mg mL^{-1}), which was diluted to 50, 37.5, 25, 18.8, 12.9, 9.4, 6.3, 4.7, 3.2, 2.4, 1.6, 1.2, 0.8, and 0.6 μg mL^{-1} of ADT. With measuring the absorption spectrum of ADT, absorbance value at 432 nm was recorded. And then a scatter plot was drawed, while the concentration was regarded as the abscissa and absorbance as the ordinate. With fitting the straight line, the standard curve equation and correlation coefficient (R^2) were obtained.

Photothermal, photothermal stability and photothermal conversion efficiency experiments of PCoA@M. 300 μL different concentrations of PCoA@M (0, 0.125, 0.25, 0.5, and 0.75 mg mL^{-1}, the concentration was quantified by the mass of PDA) were irradiated with 808 nm laser (1 W cm^{-2}, 5 min), and the temperature change was recorded by an infrared thermal imager. Similarly, 0.9 mg mL^{-1} PCoA@M under different laser irradiation (0.25, 0.5, 0.75, 1.0, 1.5, and 2.0 W cm^{-2}) was also recorded the temperature change, and 4 parallels were set up in each group.

300 μL of PCoA@M (0.90 mg mL^{-1}) in detachable 96-well plate was irradiated with laser (1.5 W cm^{-2}, 5 min) and cooled to room temperature without irradiation. Additional three laser on/off cycles were further repeated, and the whole process of temperature change was recorded with an infrared thermal imager. After measuring the absorption value of PCoA@M at 808 nm, 0.3 mL of PCoA@M in a quartz cuvette was irradiated with 808 nm laser (1.5 W cm^{-2}, 5 min), and laser was subsequently turned off, while the temperature change was recorded throughout the process.

Cytotoxicity experiments of PCo@M. 4T1 and C26 cells in logarithmic growth phase were respectively inoculated into 96-well plates and cultured in a 5% CO_2 incubator at 37 °C overnight, then the medium was removed. 200 μL of serum-free medium containing different concentrations of PCo@M (0, 0.008, 0.016, 0.032, 0.063, 0.13, 0.25, 0.5, and 1 mg mL^{-1}) were added respectively, where concentration of PCo@M was quantified by concentration of PDA. 6 parallel wells in each group were set up. After 24 h, the culture medium was removed, and cells were washed three times with PBS. 200 μL of fresh serum-free medium containning 20 μL of CCK-8 solution was added to

each well, after incubation for 4 h, the absorbance value at 450 nm was measured with a microplate reader, and then the cell viability was calculated. Cell viability (%)=(experimental group-blank group)/(control group-blank group)

Stability and blood compatibility test of PCoA@M. The lyophilized PCoA@M was respectively dissolved in PBS, DMEM, and DMEM+50% serum. After measurement of particle size, PDI, and zeta potential, PCoA@M was stored at room temperature and repeated the above test every day to investigate the stability of probe. The same amount of PCoA@M was dissolved in H_2O, PBS, NaCl, DMEM, 1640, 10% FBS, 50% FBS, and FBS, respectively, and observed whether there existed precipitation after 24 h.

1.5 mL of fresh blood acquired from 2 six-week-old KM mice (purchased from Beijing Weitong Lihua Biotechnology Co., Ltd) was transfered into EDTA k2 anticoagulant tube. The supernatant was removed by centrifuging at 700 g for 5 min. After washing several times with PBS, 10 μL of the suspension was added to 250 μL of PBS containing 0.05, 0.1, 0.2, 0.4, 0.8, and 1.6 mg mL^{-1} of PCoA@M. Here, PBS and ultrapure water were regarded as negative control and positive control respectively. With culturing for 1 and 6 h in a 5% CO_2 incubator (37 °C), a picture was taken after centrifugation (4000 rpm, 5 min), the supernatant was to measure the absorbance at 577 nm, and the cell pellet made into a cell smear was to be observed the cell morphology under a microscope. Finally, the hemolysis rate of red blood cell was calculated. Hemolysis rate (%)=$(OD_{sample}-OD_{PBS})/(OD_{water}-OD_{PBS})\times100\%$

Release of cobalt ion and ADT. PCoA@M (3 mg) was fixed with a dialysis bag and placed in a beaker containing 80 mL of PBS separately, and divided into (1) pH 5.5, (2) pH 6.0, and (3) pH 7.2 groups. At 10 min, 1, 2, 4, 8, 12, 24, 72, and 96 h, 200 μL of the solution was taken out for detection. 9 aliquots of PCoA@M were separately incubated in 200 μL of water (pH 6.0) for 10 min, 1, 2, 4, 8, 12, 24, 72, and 96 h, then dissolved and centrifuged in 1 mL of methanol. The supernatant was taken out to measure the ADT absorption spectrum with a UV-Vis spectrophotometer.

In vivo toxicity test of PCoA@M. A total of 15 four-week-old BALB/c male mice (SPF grade) were randomly divided into 3 groups, of which two groups were injected with 200 μL of PCoA@M and PBS through the tail vein respectively, and the mice were sacrificed at the first day. After 200

μL of PCoA@M was intravenously injected, the third group was sacrificed at 30th d. Fresh blood was collected for blood routine and blood biochemical analysis. Concurrently, the organs of each group were weighed to calculate the organ index and analyzed by HE staining. Organ index=organ weight/mouse weight×100%.

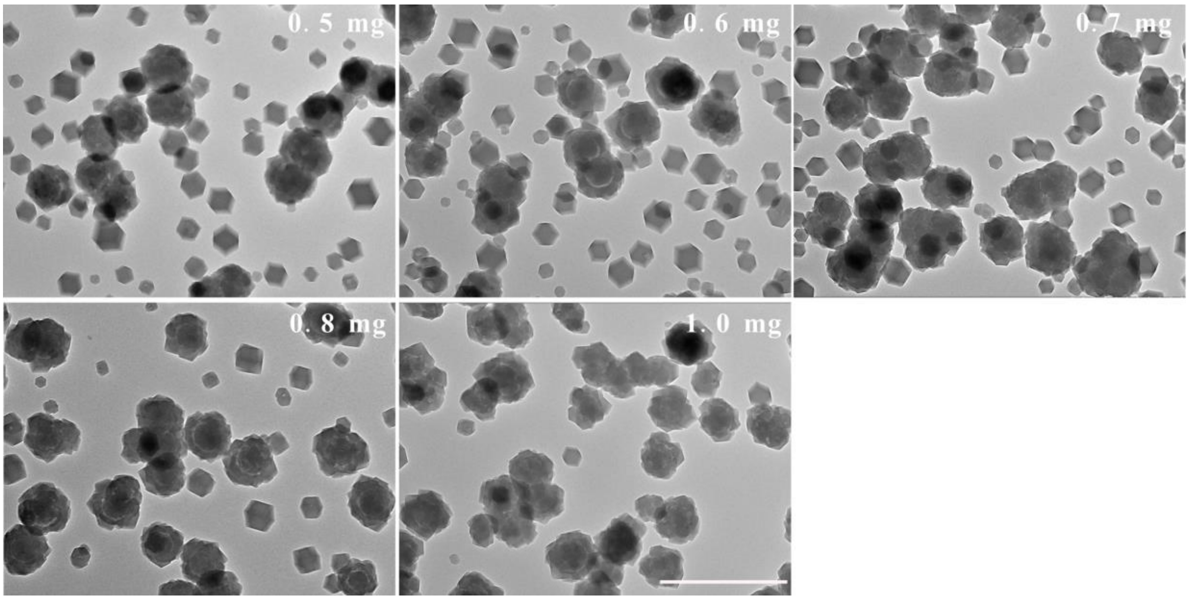

Supplementary Fig. 1 TEM of heterostructure formation under different reaction conditions. Scale bar: 500 nm.

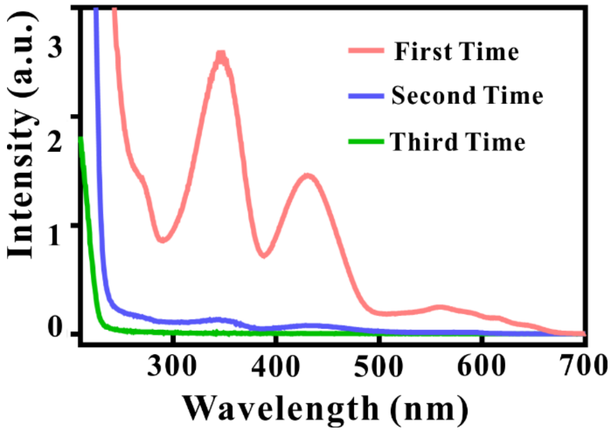

Supplementary Fig. 2 Absorption spectra of supernatant for three centrifugal purification of PCoA@M.

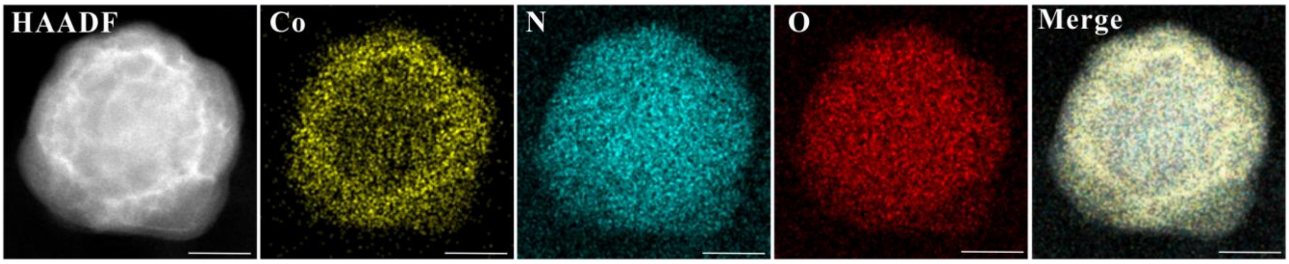

Supplementary Fig. 3 Elemental mapping of PCo, HAADF: High Angle Annular Dark Field. Scale bar: 50 nm.

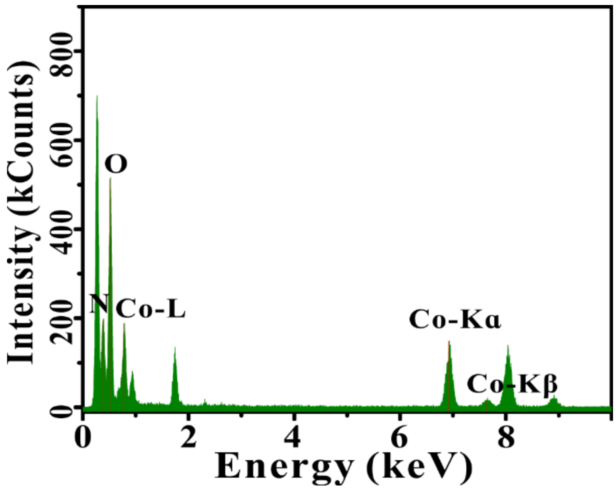

Supplementary Fig. 4 Energy dispersive spectroscopy analysis of PCo.

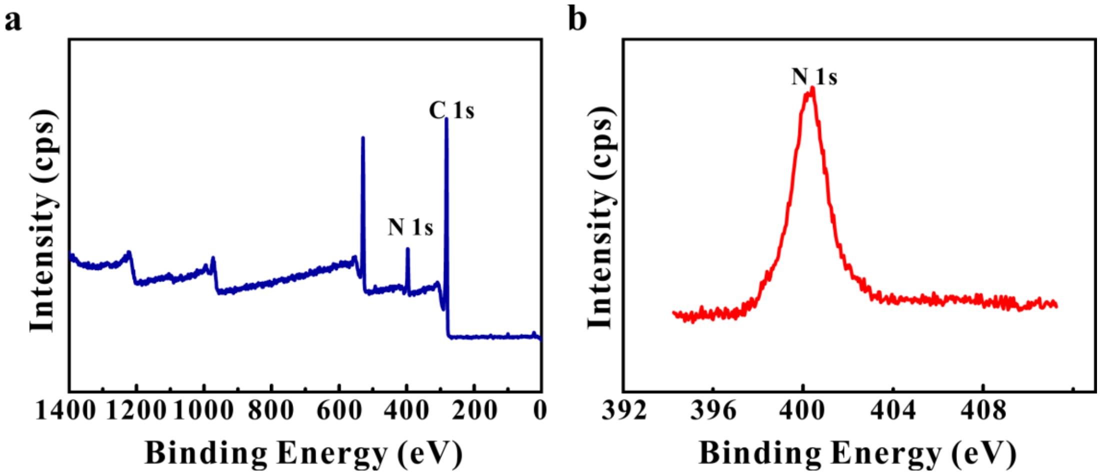

Supplementary Fig. 5 X-ray photoelectron spectroscopy of PDA (a) and high-resolution XPS spectra of N (b).

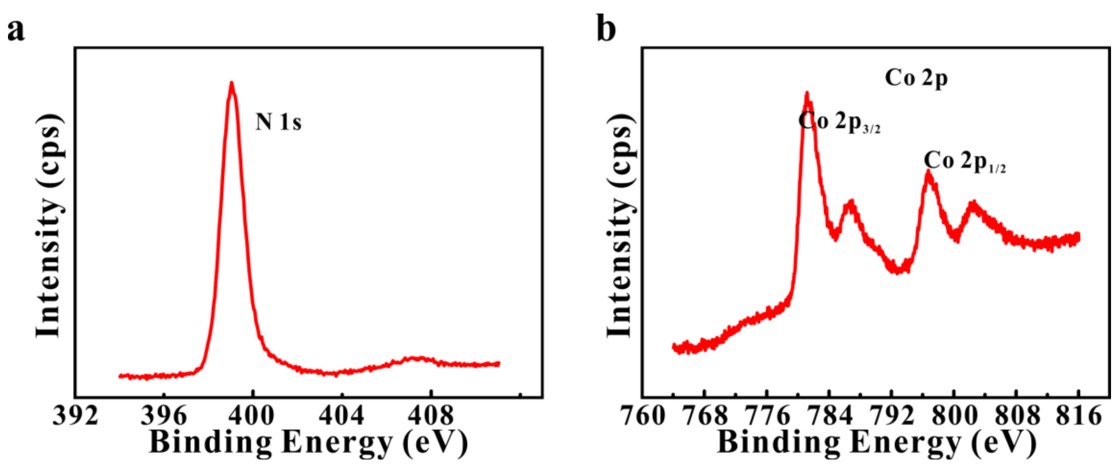

Supplementary Fig. 6 High-resolution XPS spectra of N (a) and Co (b) in PCo.

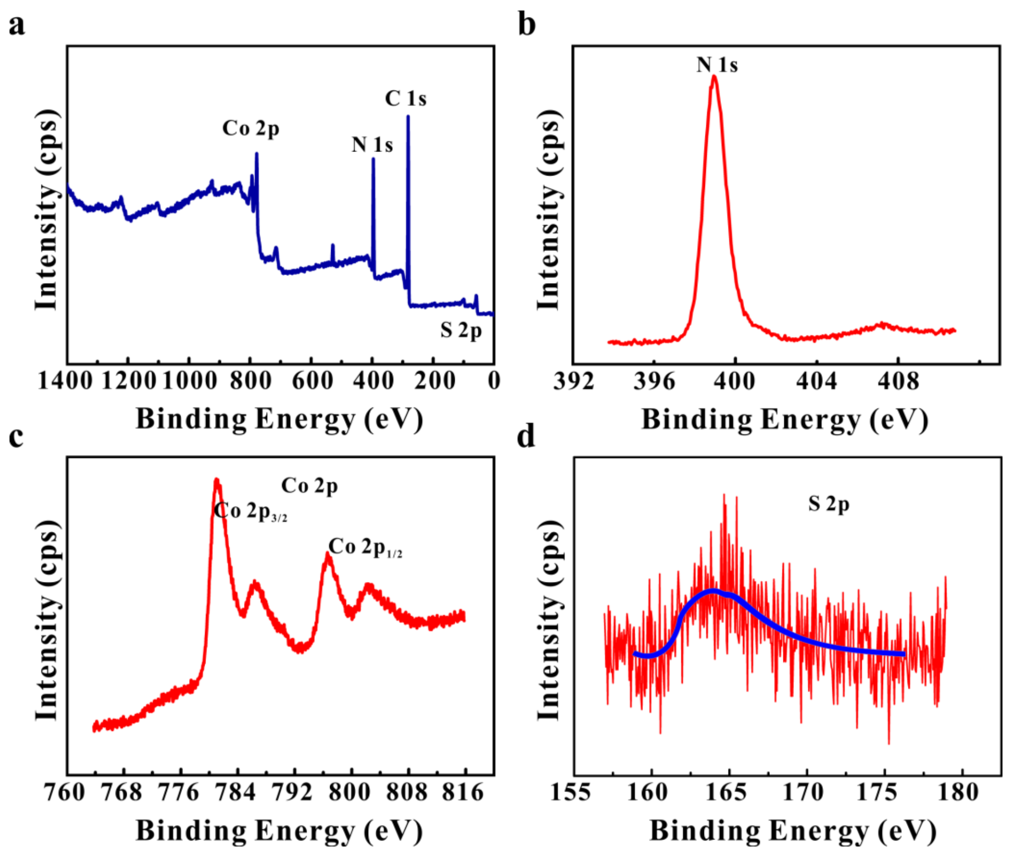

Supplementary Fig. 7 X-ray photoelectron spectroscopy of PCoA (a) and and high-resolution XPS spectra of N (b), Co (c), and S (d).

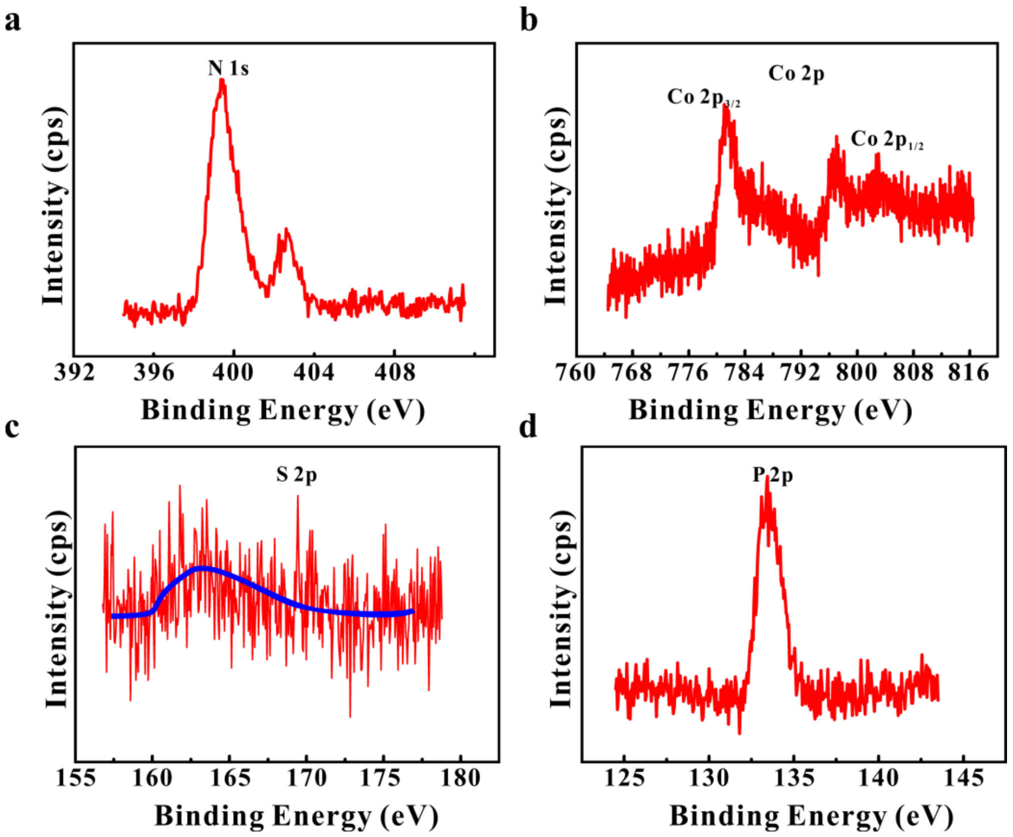

Supplementary Fig. 8 High-resolution XPS spectra of N (a), Co (b), S (c), and P (d) in PCoA@M.

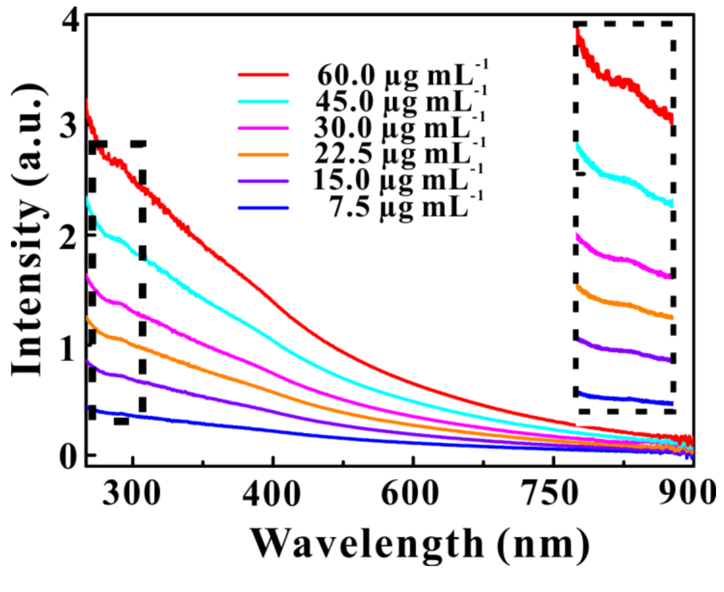

Supplementary Fig. 9 Absorption spectra of different concentrations of PDA.

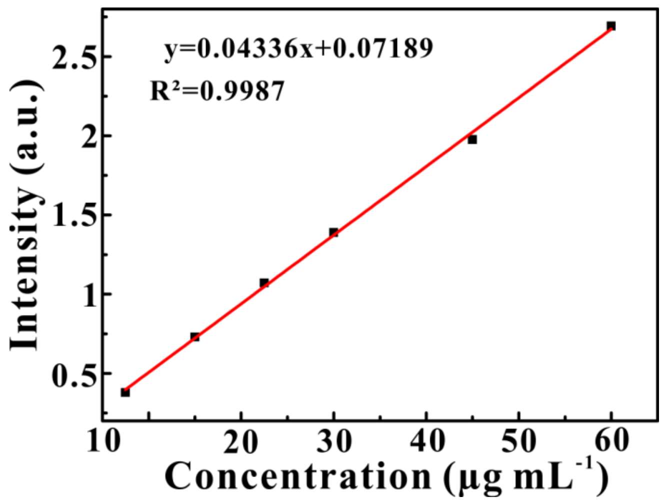

Supplementary Fig. 10 The standard curve of PDA concentration and absorption intensity.

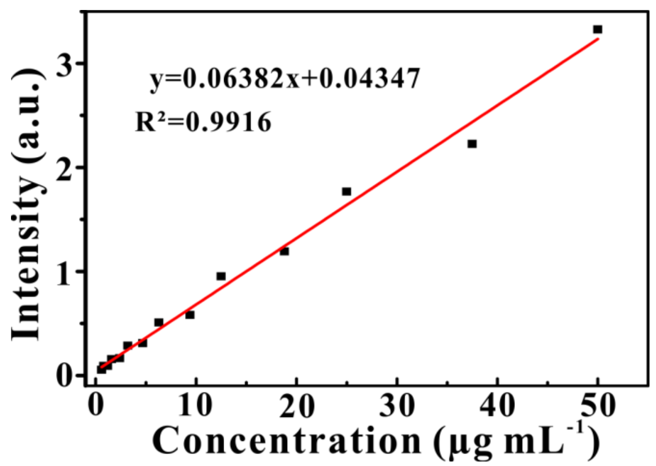

Supplementary Fig. 11 The standard curve of ADT concentration and absorption intensity.

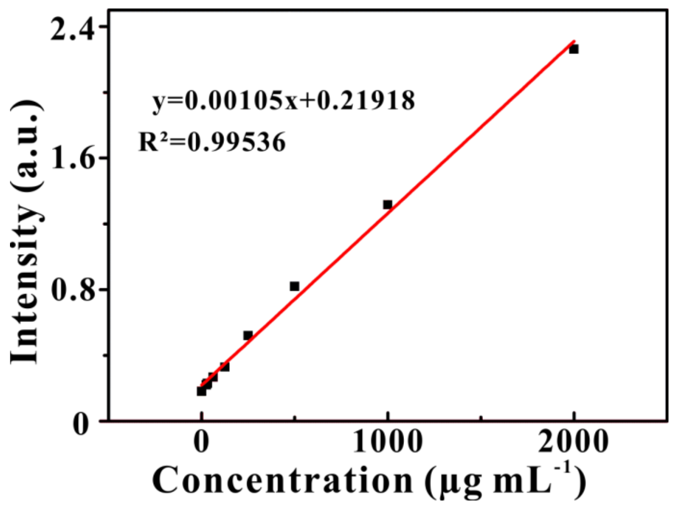

Supplementary Fig. 12 The standard curve of albumin concentration and absorption intensity.

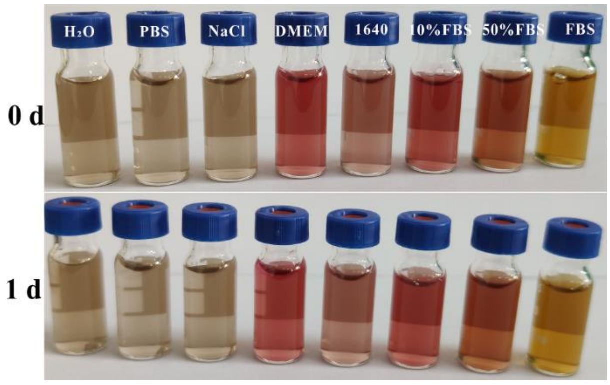

Supplementary Fig. 13 Brightfield map of PCoA@M in different media.

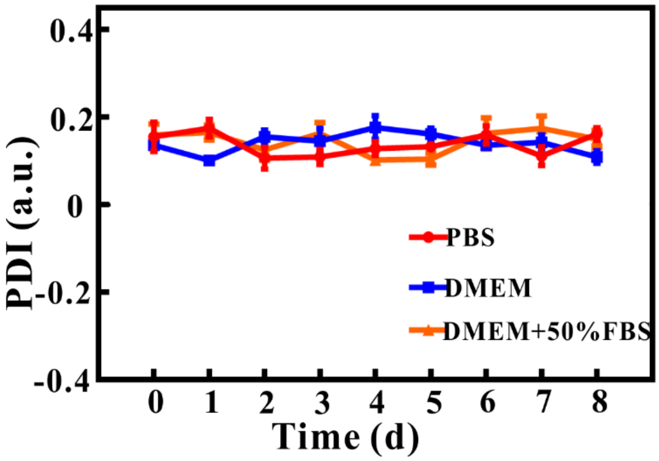

Supplementary Fig. 14 PDI of PCoA@M along with time in different media. Data are presented as the means ± s.d. (n=3 independent samples).

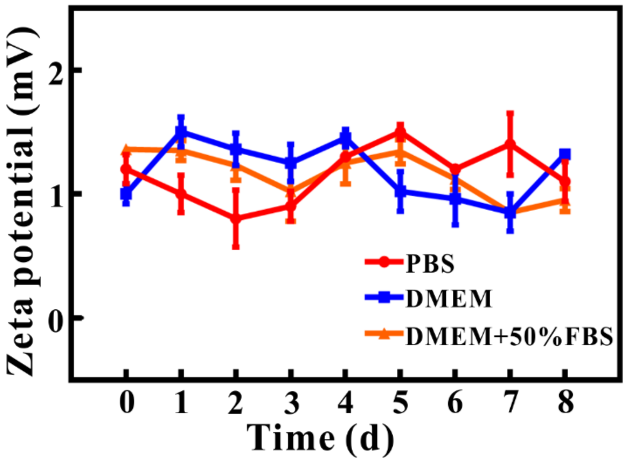

Supplementary Fig. 15 Zeta potential of PCoA@M along with time in different media. Data are presented as the means ± s.d. (n=3 independent samples).

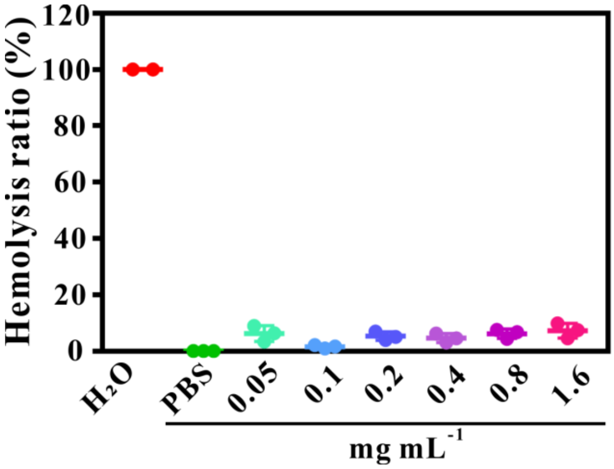

Supplementary Fig. 16 The hemolysis rates of erythrocytes after incubation with different concentrations of probe at 1 h. Data are presented as the means ± s.d. (n=3 independent samples).

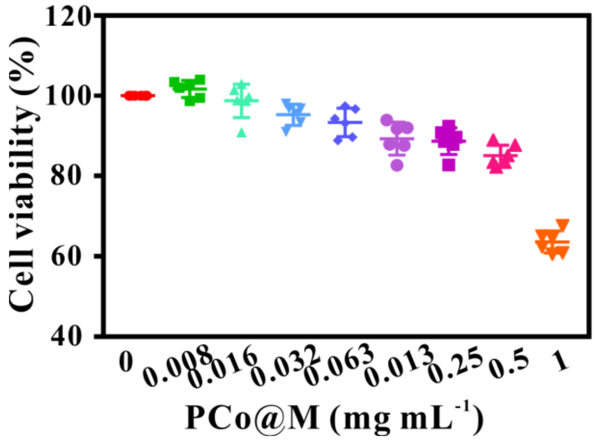

Supplementary Fig. 17 CCK-8 cytotoxicities of C26 cells incubated with different concentrations of PCo@M. Data are presented as the means ± s.d. (n=6 independent samples).

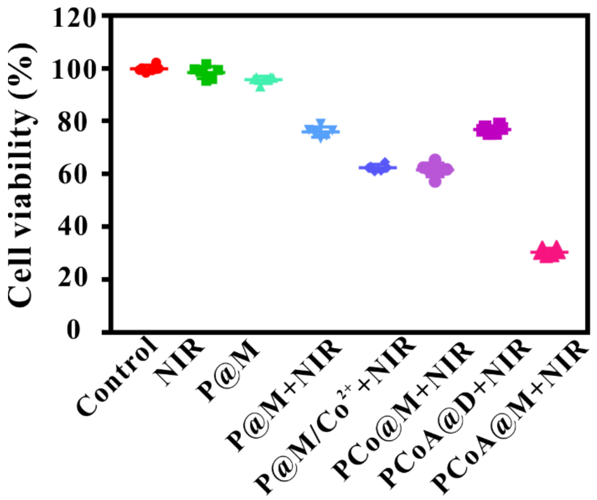

Supplementary Fig. 18 Survival rates of 4T1 cells incubated and treated with different probes (Concentration, PDA: 32 μg mL^{-1}; Co: 80 μg mL^{-1}; ADT: 4 μg mL^{-1}). Data are presented as the means ± s.d. (n=6 independent samples).

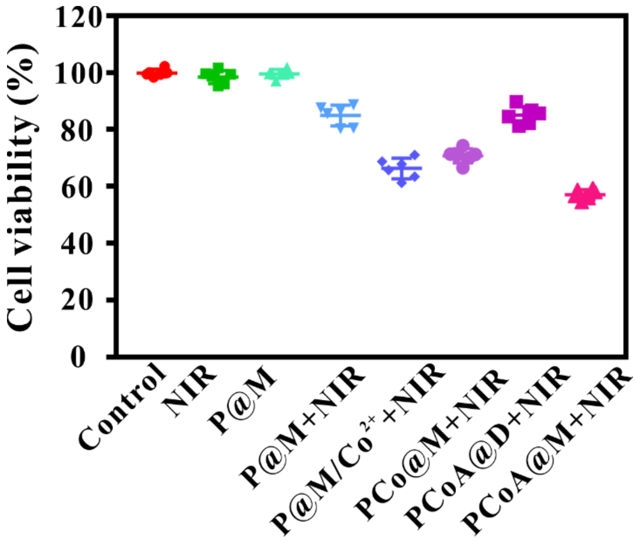

Supplementary Fig. 19 Survival rates of 4T1 cells incubated and treated with different probes (Concentration, PDA: 16 μg mL^{-1}; Co: 40 μg mL^{-1}; ADT: 2 μg mL^{-1}). Data are presented as the means ± s.d. (n=6 independent samples).

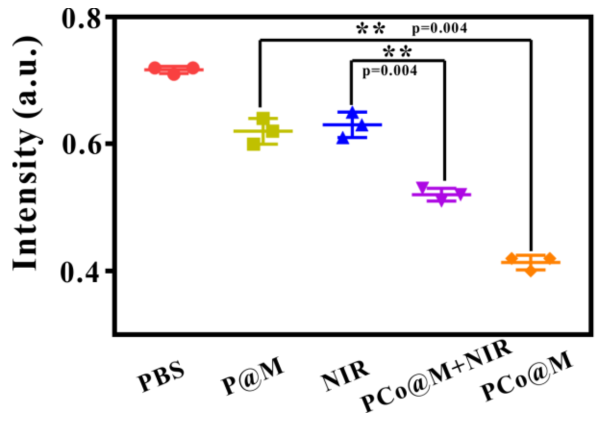

Supplementary Fig. 20 Gray-scale quantitative analysis of HSP90 in Figure 3e. Data are presented as means ± s.d. (n=3 independent samples). Statistical difference was calculated using two-tailed student's *t*-test, **: $p<0.01$.

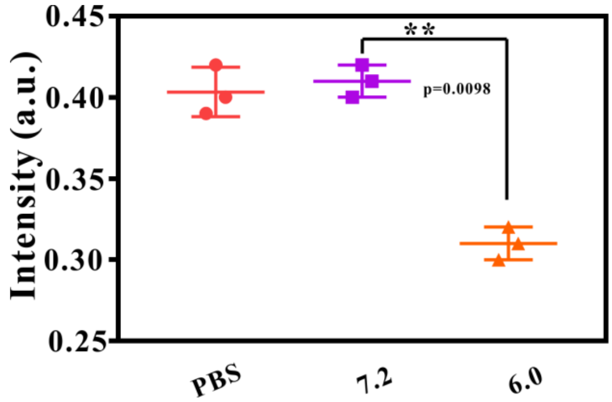

Supplementary Fig. 21 Gray-scale quantitative analysis of HSP90 in Figure 3f. Data are presented as the means ± s.d. (n=3 independent samples). Statistical difference was calculated using two-tailed student's *t*-test, **: $p<0.01$.

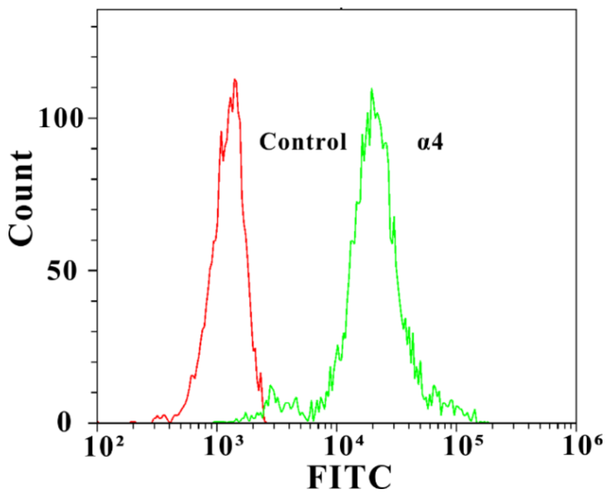

Supplementary Fig. 22 Flow cytometric analysis of α4 antigens on the cell membrane of macrophages.

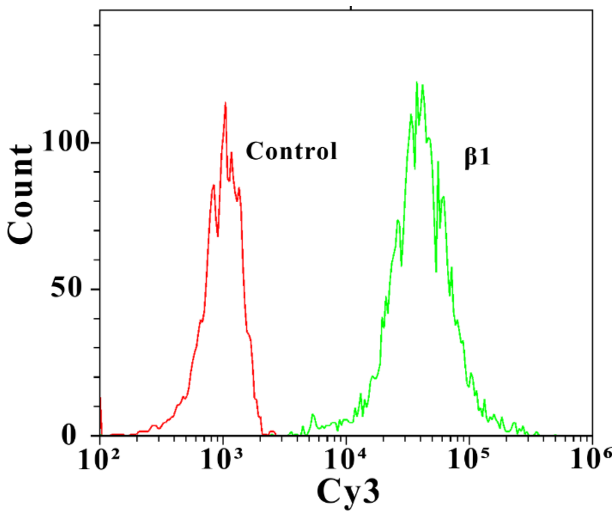

Supplementary Fig. 23 Flow cytometric analysis of β1 antigens on the cell membrane of macrophages.

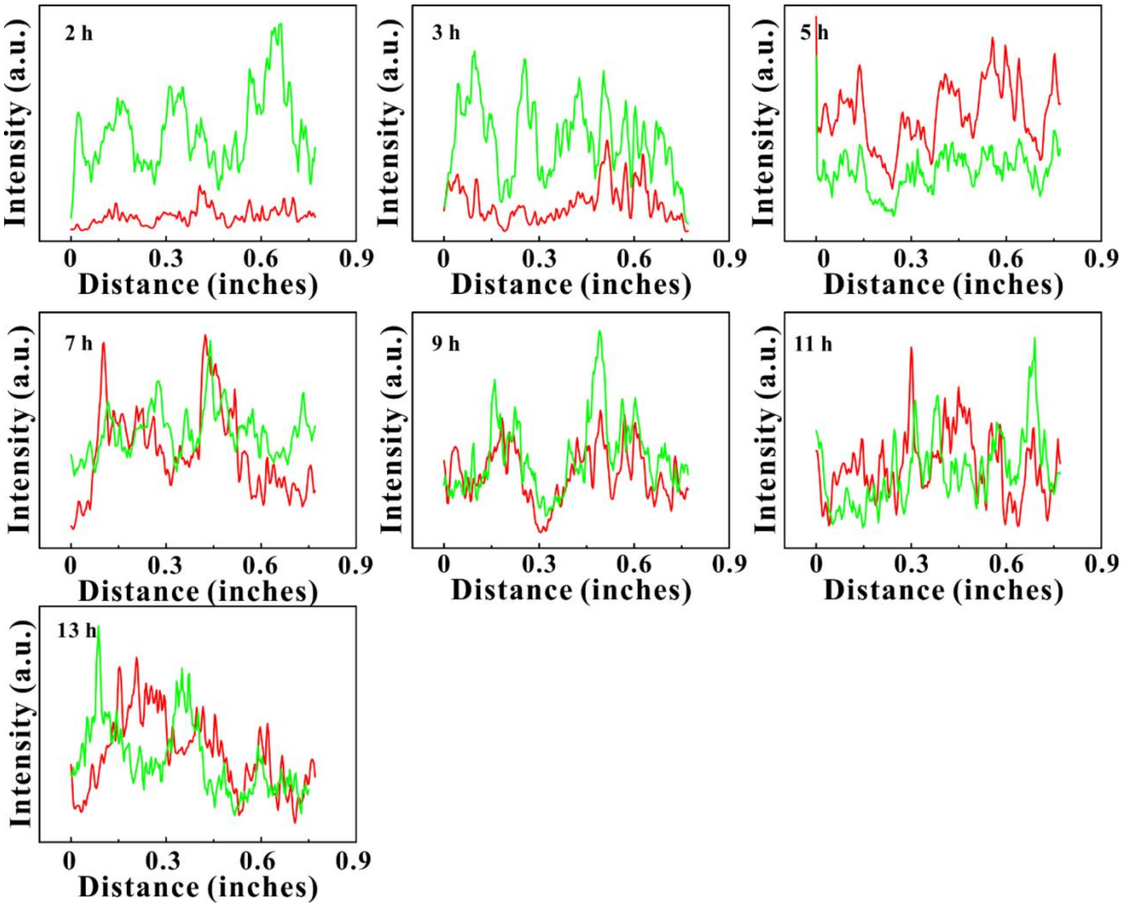

Supplementary Fig. 24 Quantitative analysis of fluorescence co-localization after Dil-labeled PCoA@M and 4T1 cells incubated for 2, 3, 5, 7, 9, 11, and 13 h and co-localized imaging.

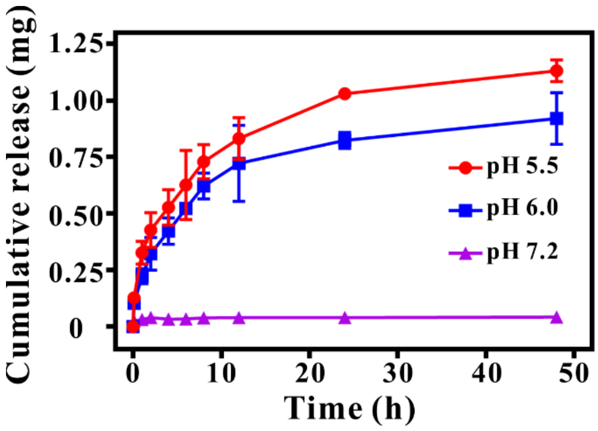

Supplementary Fig. 25 Release of cobalt ions at different times and pH. Data are presented as the means ± s.d. (n=3 independent samples).

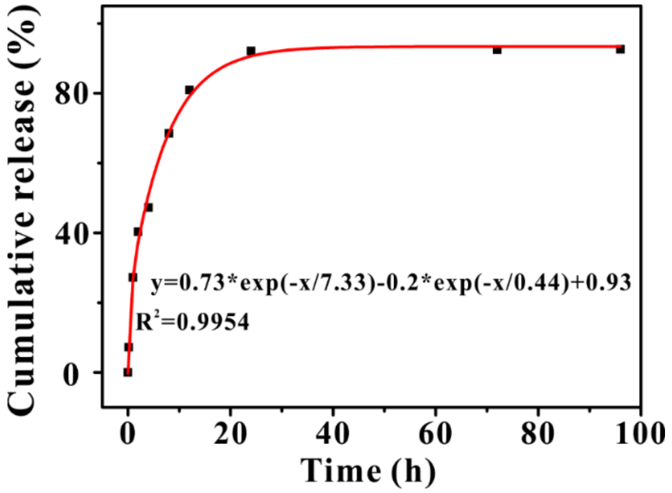

Supplementary Fig. 26 Cumulative release of ADT at pH 6.0.

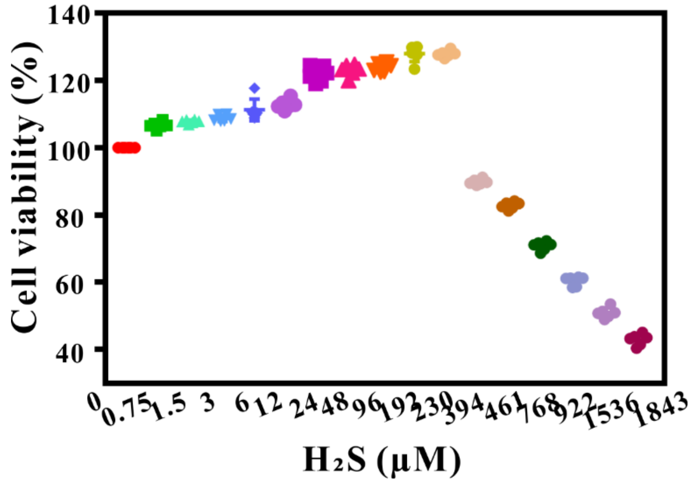

Supplementary Fig. 27 CCK-8 cytotoxicities of C26 cells incubated with different concentrations of H_2S probes. Data are presented as the means ± s.d. (n=6 independent samples).

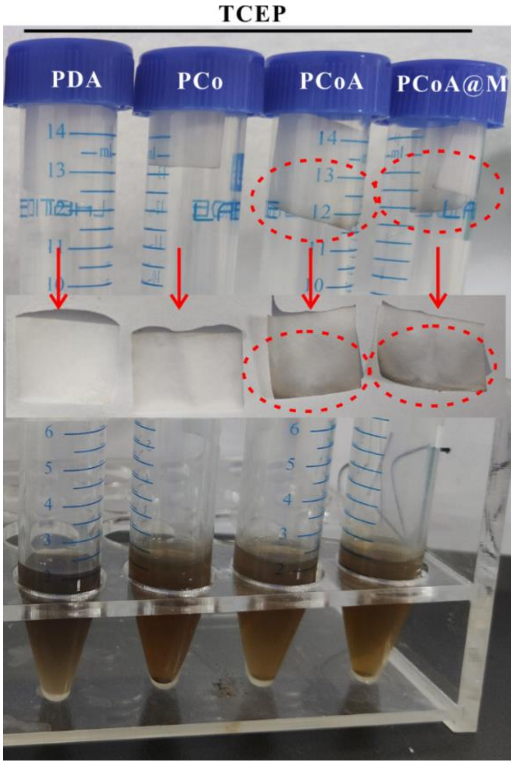

Supplementary Fig. 28 H_2S released from incubation of different probes with TCEP for 3 h under acidic condition and color reaction with lead acetate-impregnated filter paper.

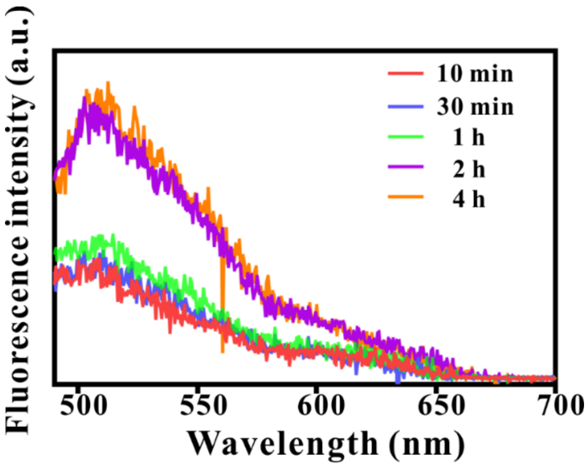

Supplementary Fig. 29 Fluorescence emission spectra after WSP-1 incubated with H_2S for different times.

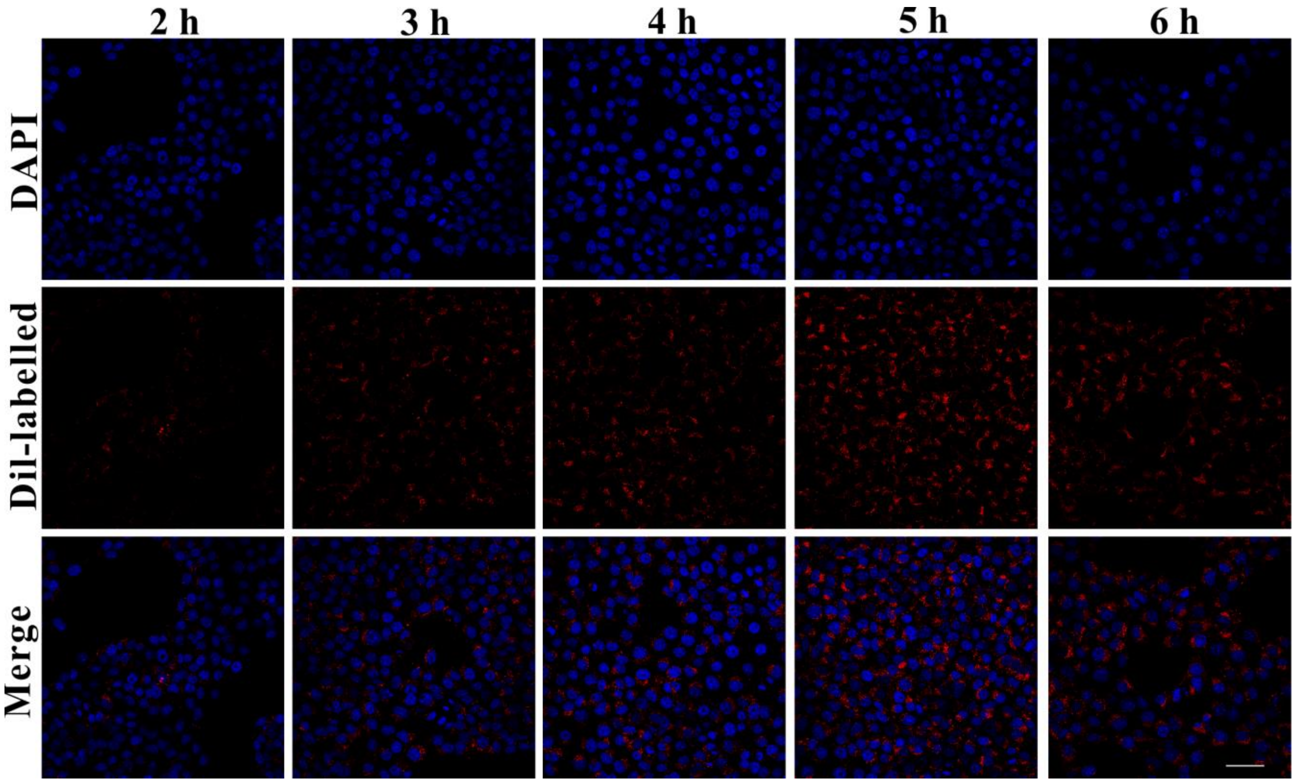

Supplementary Fig. 30 Confocal imagings of 4T1 cells after incubation with Dil-labeled PCoA@M for different times. Scale bar: 50 μm.

.

Thermal effect of PCoA@M. Next, the photothermal performance of PCoA@M was evaluated. The results exhibited the higher the concentration of PCoA@M, the faster the temperature rose (Supplementary Fig. 31a and c) under the same laser intensity. In addition, under the same concentration of PCoA@M, stronger the laser irradiation power incresaed, higher the temperature linearly raised (Supplementary Fig. 31b and d). With 0.9 mg mL^{-1} concentration and 1 W cm^{-2} laser power, the temperature of PCoA@M raised nearly 20 °C (Supplementary Fig. 31b). From the view of on-off laser irradiation cycle of probe (0.9 mg mL^{-1}, 2W cm^{-2}), PCoA@M had a good photothermal stability (Supplementary Fig. 31e). Because I was 600 mW, τ was 209.37 s (Supplementary Fig. 31f and g), A_{808} was 0.86, T_{max} was 59.4 ºC, and T_{sur} was 25.6 ºC, then according to formula 2[2], the photothermal conversion efficiency of PCoA@M was calculated as 39.33%, showing that PCoA@M presented a good photothermal conversion efficiency, and its application prospect in the field of tumor therapy was broad.

$$\eta = \frac{h(T_{\text{Max}}\text{-}T_{\text{Surr}})\text{-}Q_{\text{Dis}}}{I(1-10^{-A_{808}})} \quad (1)$$

where h=mc/τ, Q_{Dis}=$h\times(T_{\text{max water}}\text{-}T_{\text{surr}})$, m is the mass of the solution, c is specific heat capacity of

water, h is heat transfer coefficient, T_{max} is the reached highest temperature after laser irradiation, and T_{surr} is starting temperature of laser irradiation, Q_{Dis} is the energy emitted by the solute, I is the laser intensity value, and A_{808} is the absorbance value of probe at 808 nm.

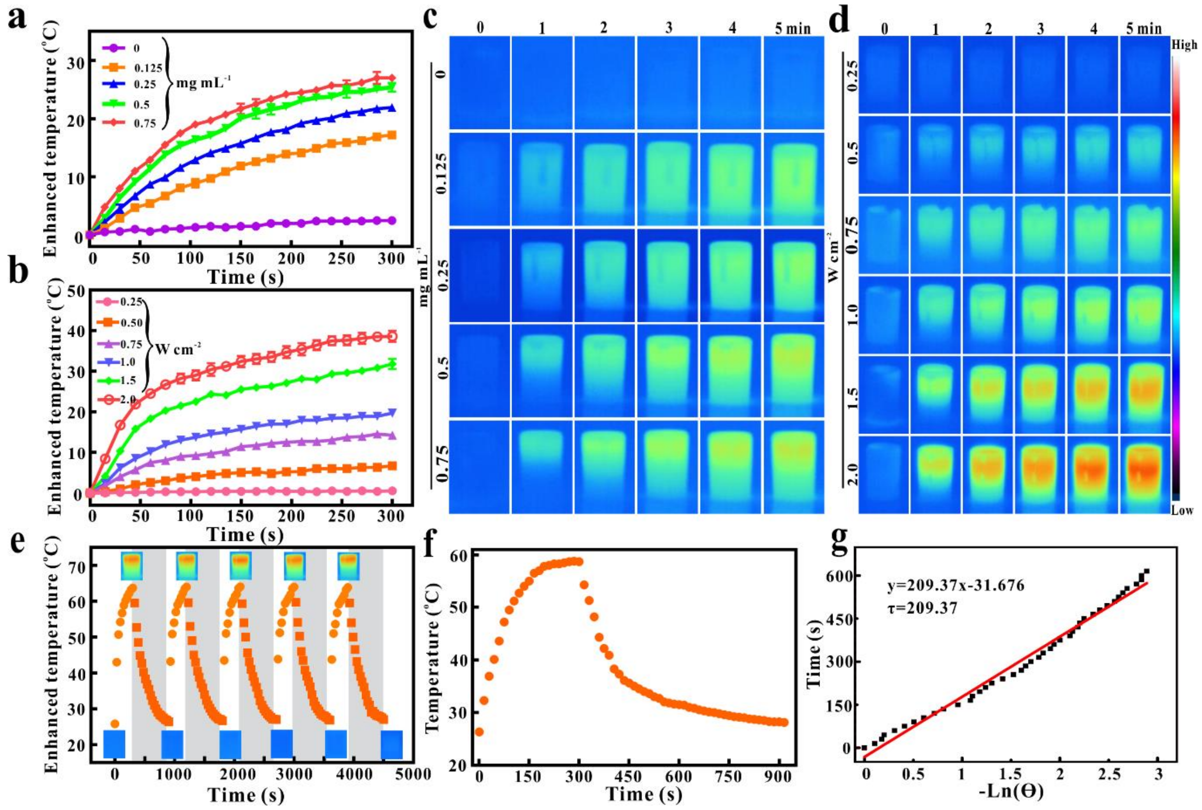

Supplementary Fig. 31 Thermal effect of PCoA@M. **a** Temperature changes and **c** thermal imagings of different concentrations of PCoA@M under laser treatment (1.5 W cm^{-2}). Data are presented as the means ± s.d. (n=3 independent samples). **b** Temperature changes and **d** thermal imaging of 0.9 mg mL^{-1} probe under different laser treatment. Data are presented as the means ± s.d. (n=3 independent samples). **e** Thermal stability of PCoA@M under repeated laser irradiation (2 W cm^{-2}) for five times, the illustrations were the thermal images of the corresponding time points. **f** Rise-fall temperature of PCoA@M and **g** calculation of τ value chart.

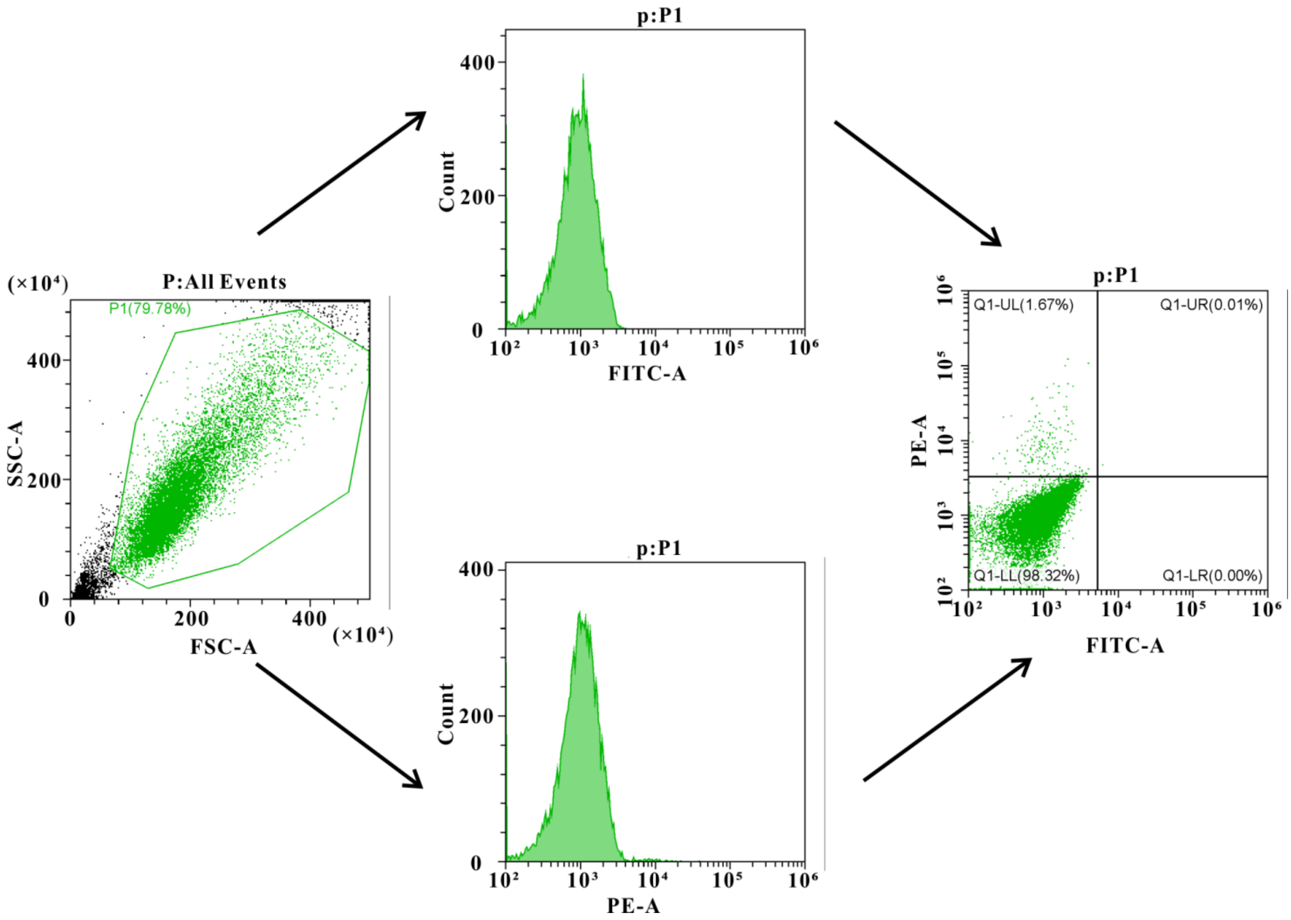

Supplementary Fig. 32 Gating strategy for determining FITC and PI staining.

In vivo safety of PCoA@M. To clarify whether the probe had a potential in vivo toxicity, safety of the probe was examined through blood biochemical indicators. The results showed, compared with PBS group, no difference in the white blood cells (WBC), red blood cells (RBC), platelets, (PLT) and hemoglobin (HGB) of healthy mice after 1 and 30 d probe injection (Supplementary Fig. 33a-d). Alanine aminotransferase (ALT), aspartate aminotransferase (AST), creatinine (CR), and uric acid (UA) were also within the normal floating range (Supplementary Fig. 33e-h), demonstrating that no significant effect on liver and kidney of mice. Organ index is one of the indicators for health. So, after injection of probe, the change of organ index in normal mice was analyzed to evaluate the influence of PCoA@M on organs. The results revealed that the organ index (liver, spleen, lung, and kidney of healthy mice) had no significant difference from the control group (Supplementary Fig. 33i-l). The above results further verified the in vivo low toxicity and excellent biological safety of PCoA@M. Moreover, HE results showed that after injection of PCoA@M, the morphology and structure of each tissue cell remained normal and clear after 1 and 30 d (Supplementary Fig. 33m), where the transverse striae of heart cell was clearly visible; the

liver lobules and other characteristic structures displayed no obvious edema; the splenic corpuscles were clearly structured with central artery; clear alveoli without alveolar wall thickening could be seen in lungs; kidney globules and the surrounding renal tubules were clearly distributed and there was no swelling of villi and no obvious macrophage infiltration in the small intestine, indicating that the organs and tissues were normal, and PCoA@M would not cause pathological change. The above experimental results confirmed that PCoA@M had biocompatibility and high long-term safety in vivo, which laid a superb foundation for in vivo application of the probe.

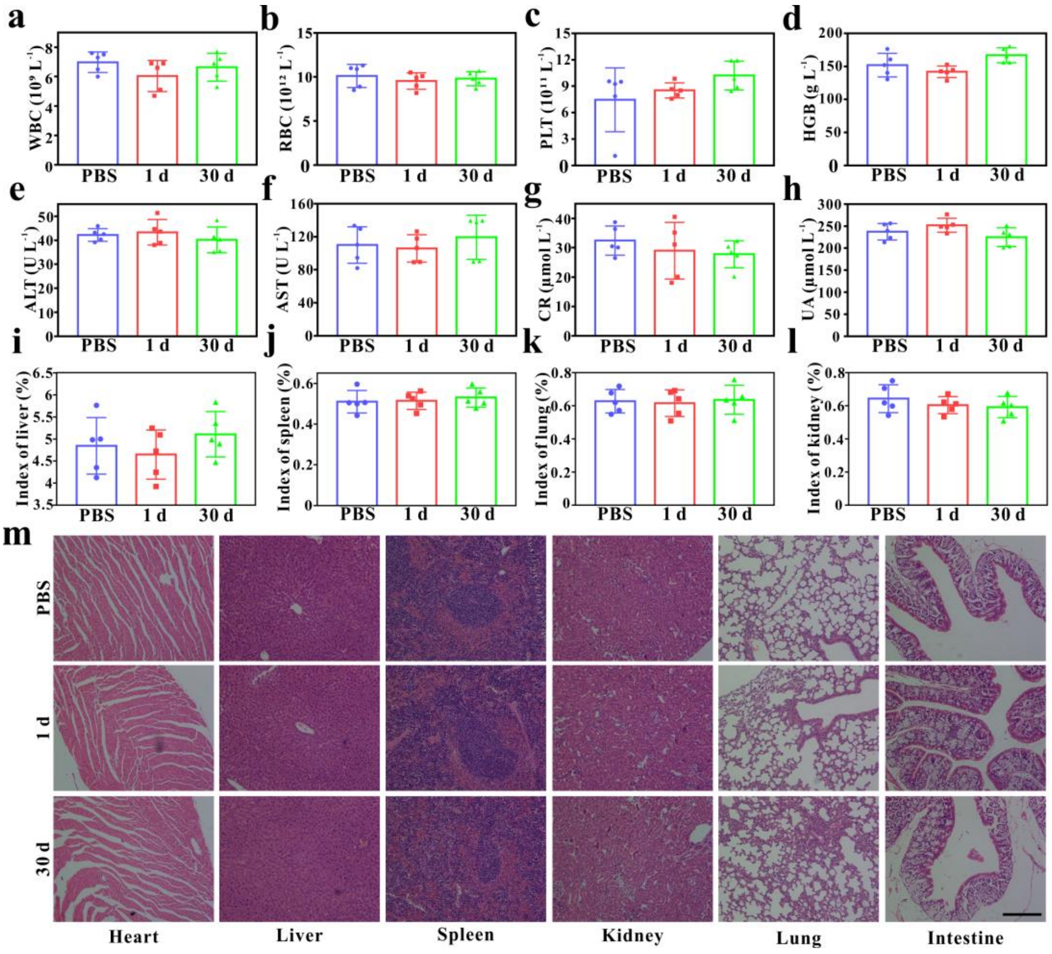

Supplementary Fig. 33 Biosafety of probes in vivo. **a-d** Blood analysis of mice after injection of PCoA@M: WBC, RBC, PLT, and HGB. Data are presented as the means ± s.d. (n=5 independent samples). **e-f** Liver function index analysis: ALT and AST. Data are presented as means ± s.d. (n=5 independent samples). **g-h** Kidney function index analysis: CR and UA. Data are presented as the means ± s.d. (n=5 independent samples). **i-l** The corresponding organ indexes of liver, spleen, lung, and kidney. Data are presented as the means ± s.d. (n=5 independent samples). **m** HE staining sections of corresponding organs after injection of PBS and probe for 1 and 30 d. Scale bar: 100 μm.

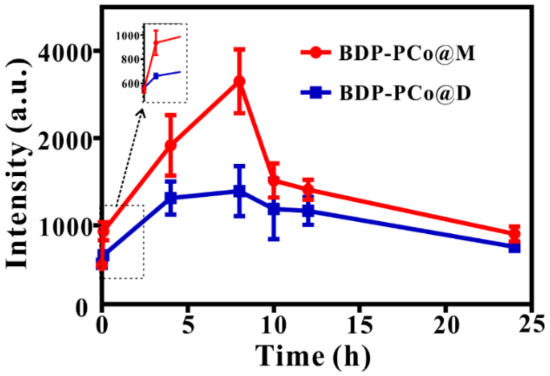

Supplementary Fig. 34 Changes of fluorescence intensity at tumor site in tumor-bearing mice after injection of BDP-labeled PCo@M and PCo@D. Data are presented as the means ± s.d. (n=4 independent samples).

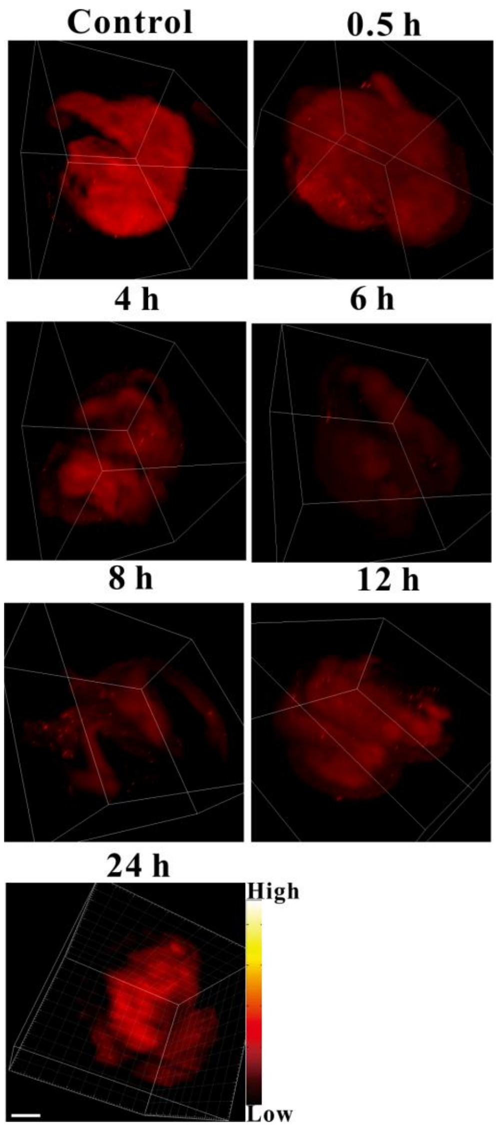

Supplementary Fig. 35 3D deep-hypothermia fluorescence imagings of NADH. Colour scale: black to white represent fluorescence intensity values of NADH from low to high. Scale bar: 2 μm.

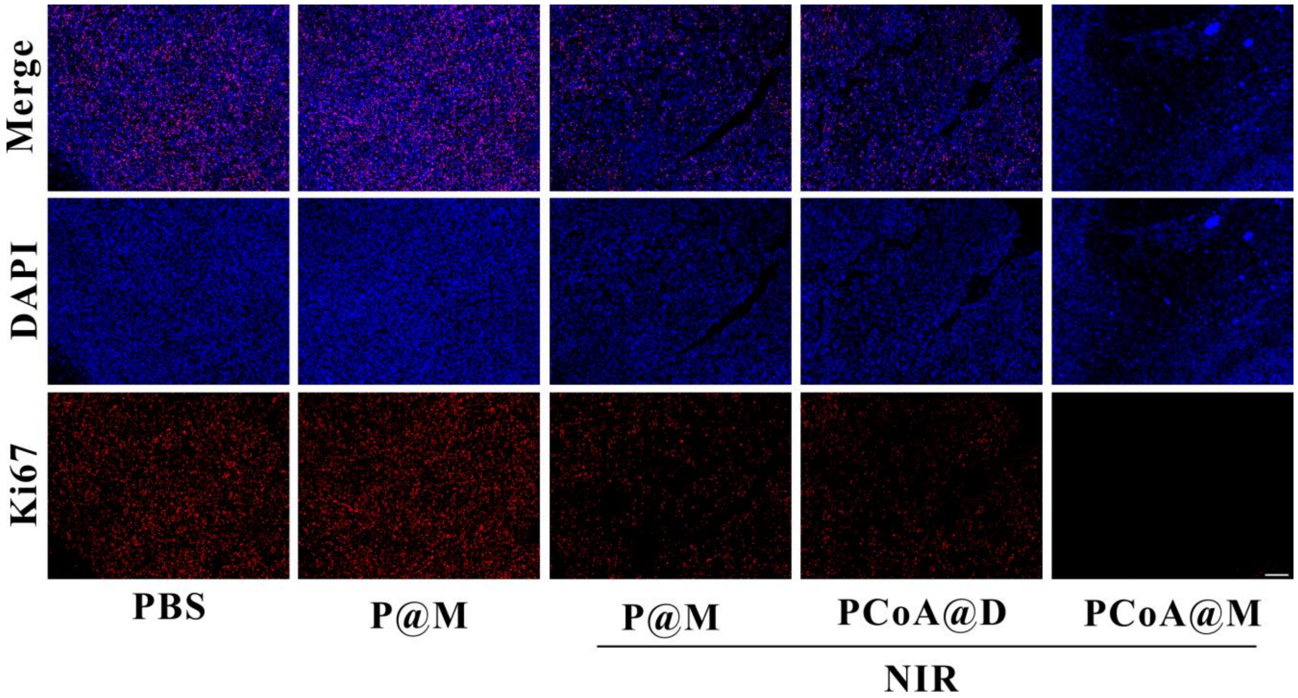

Supplementary Fig. 36 Ki67 immunofluorescence staining imagings of subcutaneous tumor site. Scale bar: 800 μm.

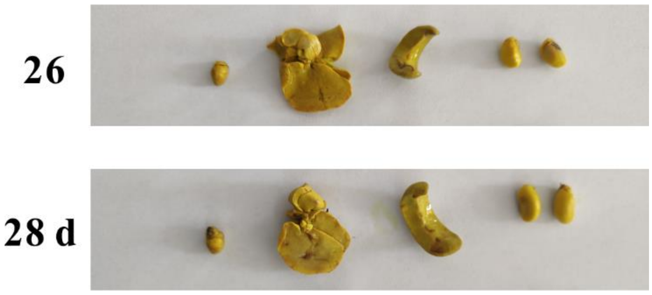

Supplementary Fig. 37 Bouins fixed organs map after PBS treatment of subcutaneous tumor.

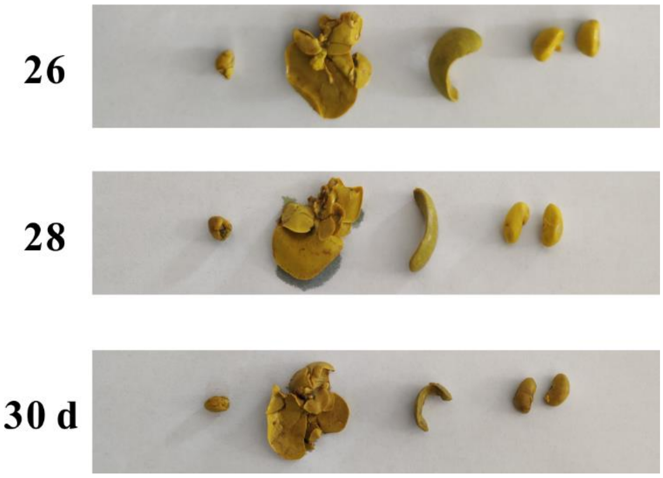

Supplementary Fig. 38 Bouins fixed organs map after P@M treatment of subcutaneous tumor.

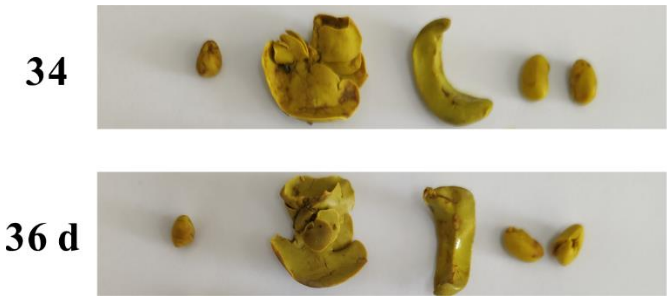

Supplementary Fig. 39 Bouins fixed organs map after P@M+NIR treatment of subcutaneous tumor.

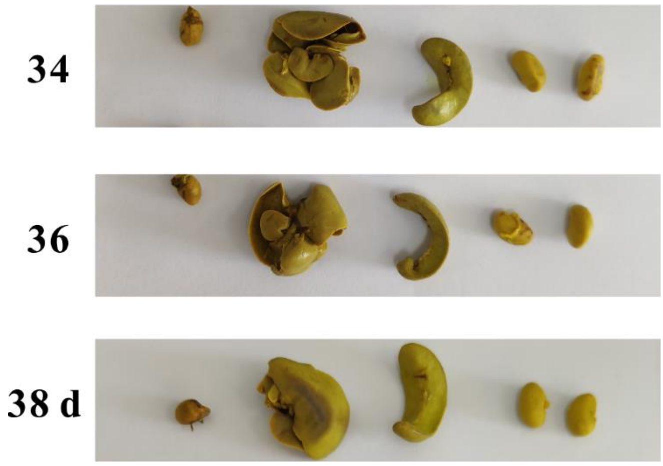

Supplementary Fig. 40 Bouins fixed organs map after PCoA@D+NIR treatment of subcutaneous tumor.

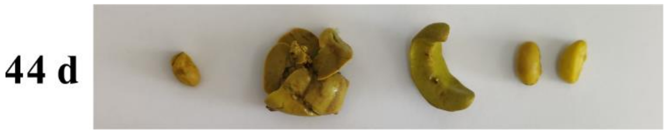

Supplementary Fig. 41 Bouins fixed organs map after PCoA@M+NIR treatment of subcutaneous tumor.

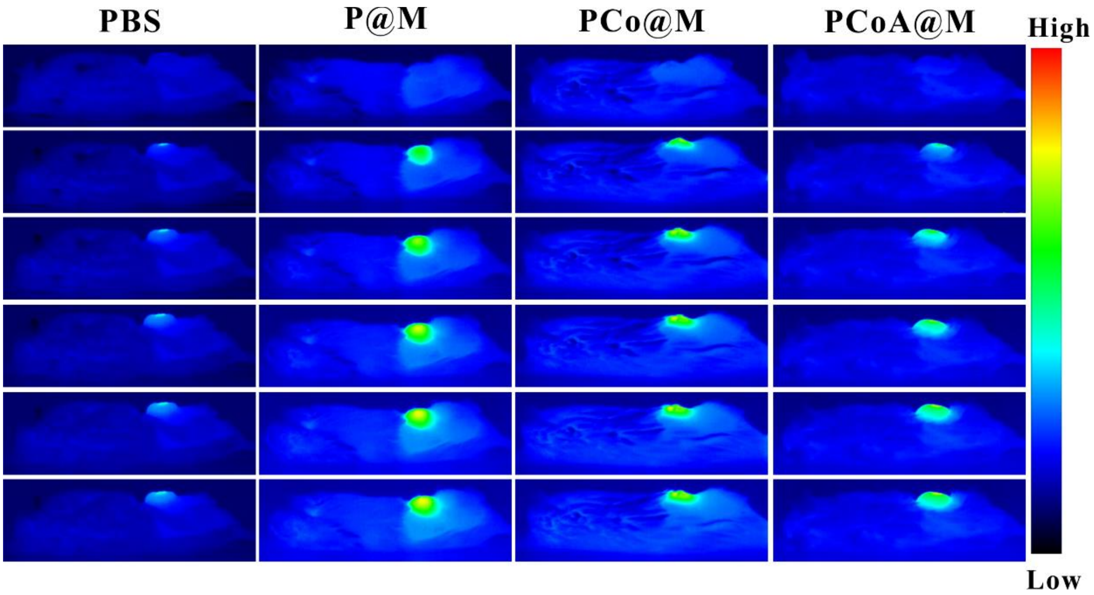

Supplementary Fig. 42 Thermal image of tumor after injection of *in situ* 4T1 tumor-bearing mice with PBS, P@M, PCo@M and PCoA@M and treated with NIR. Colour scale: black to red represent temperature of tumor from low to high.

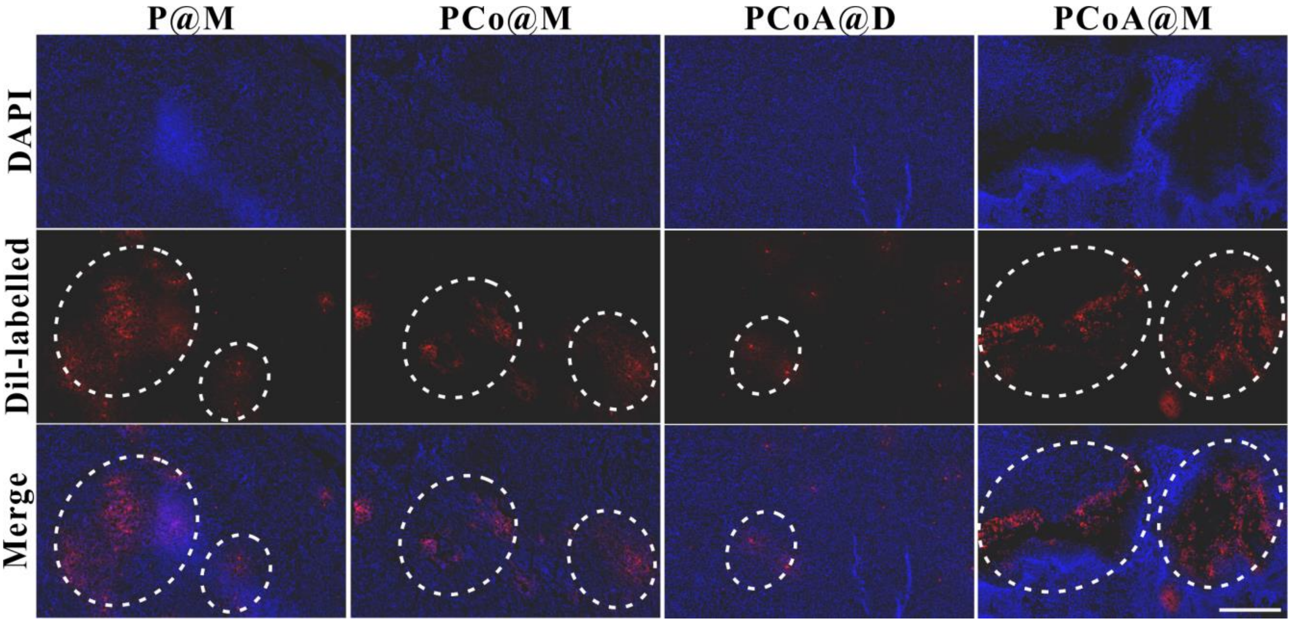

Supplementary Fig. 43 Fluorescence imagings of in situ 4T1 tumor sections after injection of different Dil-labelled probes to mice. Scale bar: 400 μm.

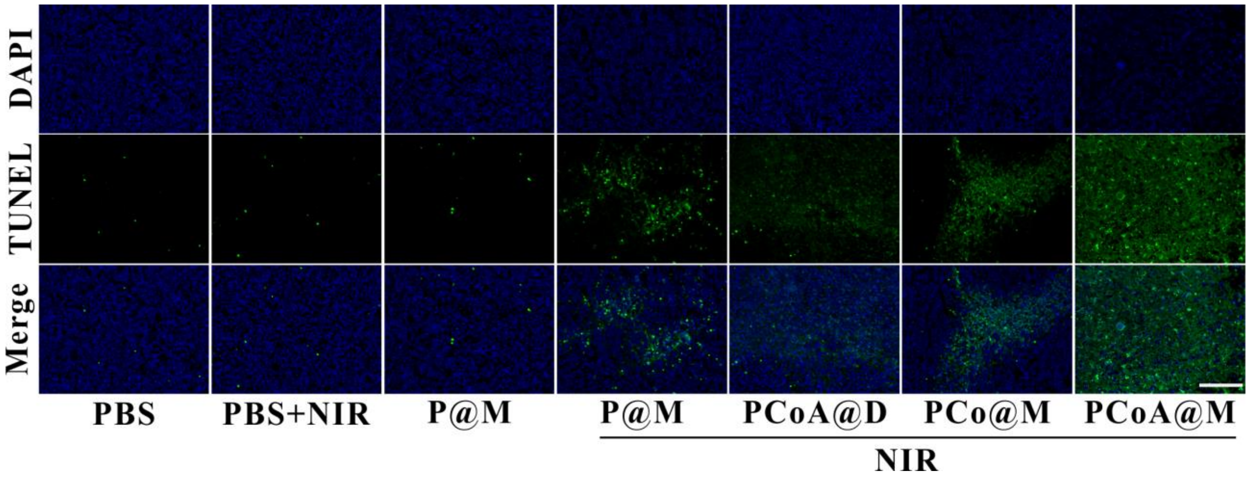

Supplementary Fig. 44 TUNEL immunofluorescence staining imagings of in situ tumor site. Scale bar: 100 μm.

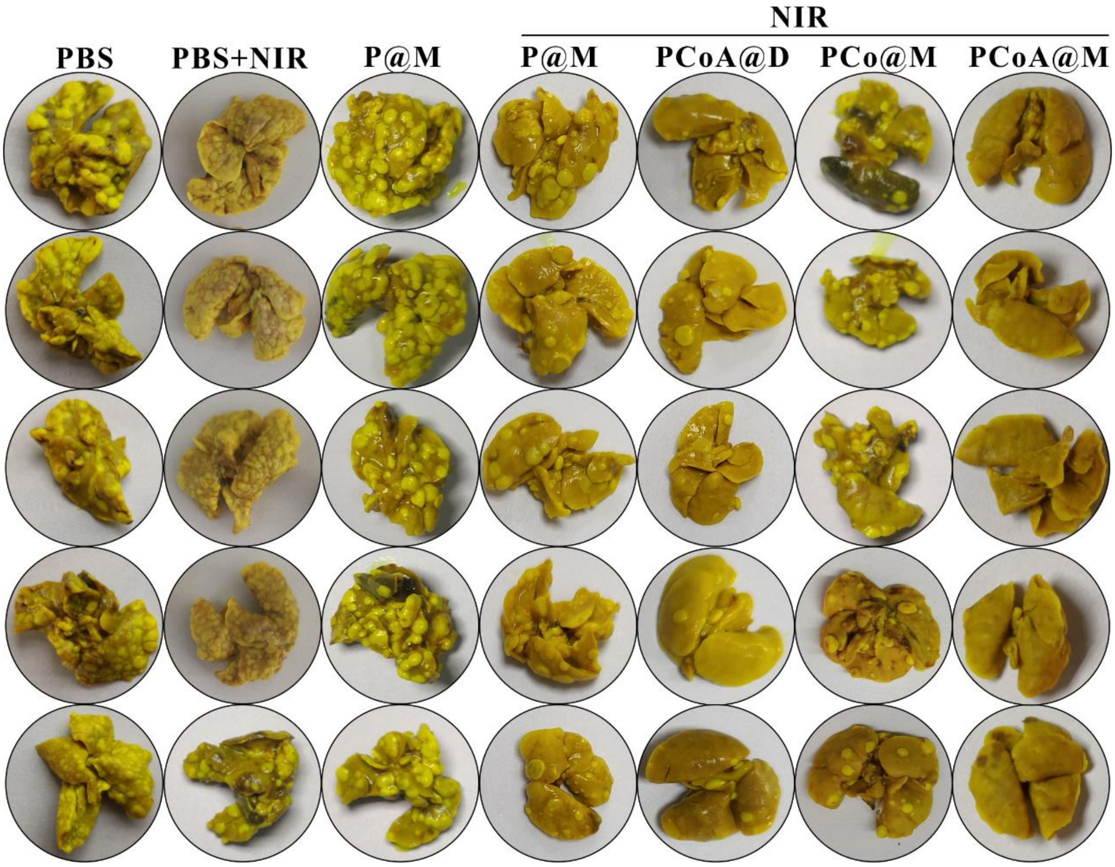

Supplementary Fig. 45 The brightfield of the lung tissue of mice with *in situ* 4T1 tumor-bearing mice after 28 d of different treatments, n=5.

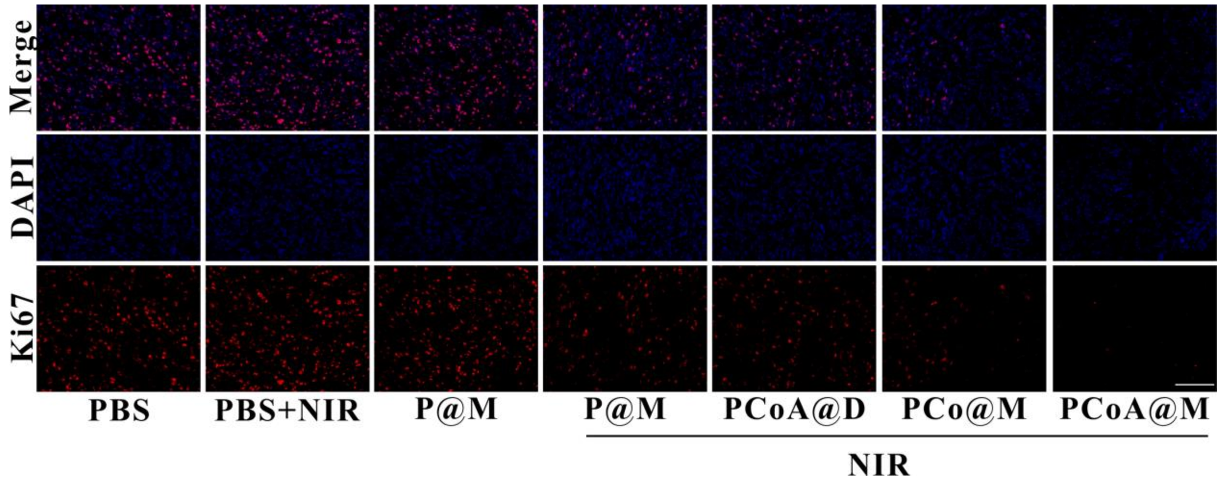

Supplementary Fig. 46 Ki67 immunostainning of 4T1 tumor in situ after treatment of different probes at 12 h. Scale bar: 100 μm.

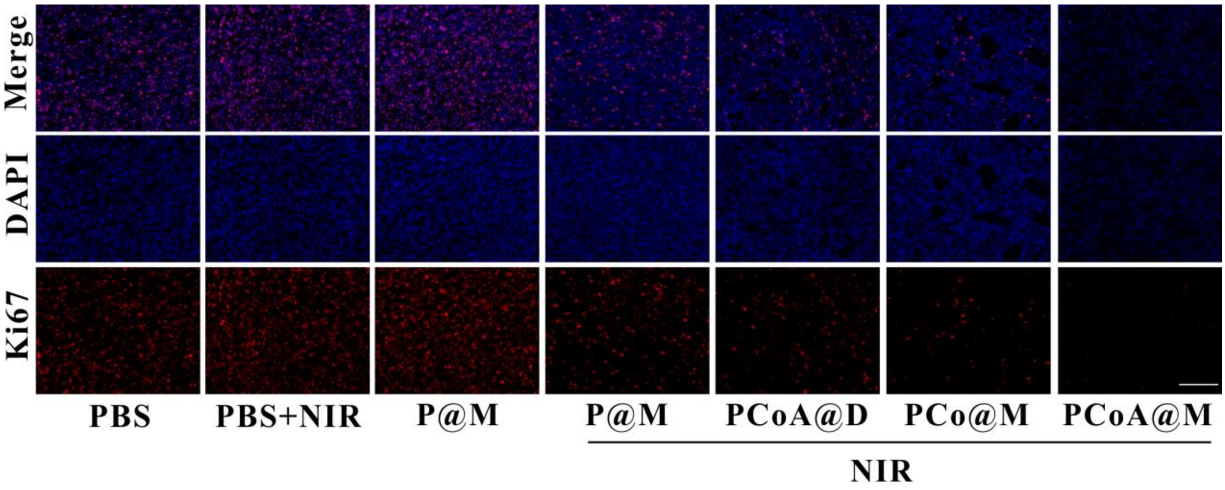

Supplementary Fig. 47 Ki67 immunostainning of 4T1 tumor in situ after treatment of different probes at 28 d. Scale bar: 100 μm.

Supplementary Table 1 Genotyping results of short tandem repeat (STR) loci and amelogenin loci of 4T1 cells used in this experiment.

Loci	submitted cell STR information			cell STR information of cell bank		
	submitted cell name: 4T1			cell name of cell bank: 4T1		
	Allele1	Allele2	Allele3	Allele1	Allele2	Allele3
STR 1-1	15	16		15	16	
STR 1-2	17			17		
STR 2-1	16	17		16	17	
STR 3-2	14	15		14	15	
STR 4-2	21.3			21.3		
STR 5-5	14			14		
STR 6-4	18			18		
STR 6-7	12			12		
STR 7-1	25.2			25.2		
STR 8-1	13			13		
STR 11-2	18	20		18	19	20
STR 12-1	16			16		
STR 13-1	16.2			16.2		
STR 15-3	22.3			22.3		
STR 17-2	15			15		
STR 18-3	18	19		18	19	
STR 19-2	13			13		
STR X-1	25			25		
D4S2408						
D8S1106						

Note: D4S2408 and D8S1106 are 2 human-derived STR marker sites to detect whether the cells are contaminated by human-derived cells.

Supplementary Table 2 Genotyping results of STR loci and amelogenin loci of RAW 264.7 cells used in this experiment.

Loci	submitted cell STR information			cell STR information of cell bank		
	submitted cell name: RAW 264.7			cell name of cell bank: RAW 264.7		
	Allele1	Allele2	Allele3	Allele1	Allele2	Allele3
STR 1-1	15	16		15	16	
STR 1-2	17			17		
STR 2-1	16			16		
STR 3-2	14			14		
STR 4-2	22.3			22.3		
STR 5-5	14			14		
STR 6-4	18			18		
STR 6-7	12	13		12		
STR 7-1	25.2			25.2		
STR 8-1	13			13		
STR 11-2	17			17		
STR 12-1	16			16		
STR 13-1	16.2			16.2		
STR 15-3	22.3			22.3		
STR 17-2	14	16		14	16	
STR 18-3	17	18		18		
STR 19-2	14			14		
STR X-1	24			24		
D4S2408						
D8S1106						

Note: D4S2408 and D8S1106 are 2 human-derived STR marker sites to detect whether the cells are contaminated by human-derived cells.

Supplementary References

1. Cheng, K. et al. Hitherto-unexplored photodynamic therapy of Ag_2S and enhanced regulation based on polydopamine in vitro and vivo. *Chem. - Eur. J.*, **25**, 7553-7560 (2019).

2. Cheng, K. et al. High-security nanocluster for switching photodynamic combining photothermal and acid-induced drug compliance therapy guided by multimodal active-targeting imaging. *Adv. Funct. Mater.* **28**, 1803118 (2018).
