# Supplementary material for: Biomimetic material degradation for synergistic enhanced therapy by regulating endogenous energy metabolism imaging under hypothermia
Source: Nat Commun. 2022 Aug 5;13:4567. doi: 10.1038/s41467-022-32349-2 (PMC9355994; doi:10.1038/s41467-022-32349-2)
Supplement: Supplementary file 3 — Reporting Summary [file 41467_2022_32349_MOESM3_ESM.pdf]

nature portfolio

Corresponding author(s): Yuan-Di Zhao

Last updated by author(s): Jun 29, 2022

Reporting Summary

Nature Portfolio wishes to improve the reproducibility of the work that we publish. This form provides structure for consistency and transparency in reporting. For further information on Nature Portfolio policies, see our Editorial Policies and the Editorial Policy Checklist.

Statistics

For all statistical analyses, confirm that the following items are present in the figure legend, table legend, main text, or Methods section.

n/a	Confirmed	
☐	☒	The exact sample size (*n*) for each experimental group/condition, given as a discrete number and unit of measurement
☐	☒	A statement on whether measurements were taken from distinct samples or whether the same sample was measured repeatedly
☐	☒	The statistical test(s) used AND whether they are one- or two-sided<br>*Only common tests should be described solely by name; describe more complex techniques in the Methods section.*
☒	☐	A description of all covariates tested
☒	☐	A description of any assumptions or corrections, such as tests of normality and adjustment for multiple comparisons
☐	☒	A full description of the statistical parameters including central tendency (e.g. means) or other basic estimates (e.g. regression coefficient) AND variation (e.g. standard deviation) or associated estimates of uncertainty (e.g. confidence intervals)
☐	☒	For null hypothesis testing, the test statistic (e.g. *F*, *t*, *r*) with confidence intervals, effect sizes, degrees of freedom and *P* value noted<br>*Give P values as exact values whenever suitable.*
☒	☐	For Bayesian analysis, information on the choice of priors and Markov chain Monte Carlo settings
☒	☐	For hierarchical and complex designs, identification of the appropriate level for tests and full reporting of outcomes
☒	☐	Estimates of effect sizes (e.g. Cohen's *d*, Pearson's *r*), indicating how they were calculated

Our web collection on statistics for biologists contains articles on many of the points above.

Software and code

Policy information about availability of computer code

Data collection	Flow-cytomery data were collected with CytExpert (ver. 2.3.0.84). Confocal images were collected with FluoView31S (ver.2.3.1.163).
Data analysis	Flow-cytomery data were analyzed with CytExpert (ver. 2.3.0.84). 2D/3D autofluorescence imaging of NADH/FAD were analyzed with Imaris x64 (ver. 9.0.1); Statistical analysis was performed with Origin Pro (ver. 8.00.000) and GraphPad Prism (ver. 7.00); ImageJ (ver. 1.4.3.67) and Matlab R2017a (ver. 1.0.0.1) were used to analyze fluorescent grayscale. Alpha EaseFC 4.0 was used to analyze the WB.

For manuscripts utilizing custom algorithms or software that are central to the research but not yet described in published literature, software must be made available to editors and reviewers. We strongly encourage code deposition in a community repository (e.g. GitHub). See the Nature Portfolio guidelines for submitting code & software for further information.

Data

Policy information about availability of data

All manuscripts must include a data availability statement. This statement should provide the following information, where applicable:

- Accession codes, unique identifiers, or web links for publicly available datasets
- A description of any restrictions on data availability
- For clinical datasets or third party data, please ensure that the statement adheres to our policy

The authors declare that the main data supporting the findings of this study are available within the article and its Supplementary Information or from the authors upon request. The raw data generated in this study are provided in the Supplementary Information/Source Data file. A reporting summary for this article is available as a Supplementary Information file.

Field-specific reporting

Please select the one below that is the best fit for your research. If you are not sure, read the appropriate sections before making your selection.

☒ Life sciences ☐ Behavioural & social sciences ☐ Ecological, evolutionary & environmental sciences

For a reference copy of the document with all sections, see nature.com/documents/nr-reporting-summary-flat.pdf

Life sciences study design

All studies must disclose on these points even when the disclosure is negative.

Sample size	To ensure the accuracy of the experiments, at least three replicates were performed. In the characterization experiments, 3-4 samples were used to analyze particle size and zeta potentials. In the cell experiments, 3-6 samples were used to analyze biocompatibility, toxicity, and therapeutic effect of probes. In antitumor experiments, 5 mice each group were used to analyze tumor volume, survival rate, body weight in mice. For other experiments, the sample size for each group was 3-5.
Data exclusions	No data were excluded from the analyses.
Replication	The experiments were reproduced successfully,the number of experiments was listed in corresponding figure legends.
Randomization	The samples were allocated into experimental groups randomly.
Blinding	The investigators were blinded to group allocation during data collection and analysis.

Reporting for specific materials, systems and methods

We require information from authors about some types of materials, experimental systems and methods used in many studies. Here, indicate whether each material, system or method listed is relevant to your study. If you are not sure if a list item applies to your research, read the appropriate section before selecting a response.

Materials & experimental systems

n/a	Involved in the study	
☐	☒	Antibodies
☐	☒	Eukaryotic cell lines
☒	☐	Palaeontology and archaeology
☐	☒	Animals and other organisms
☒	☐	Human research participants
☒	☐	Clinical data
☒	☐	Dual use research of concern

Methods

n/a	Involved in the study	
☒	☐	ChIP-seq
☐	☒	Flow cytometry
☒	☐	MRI-based neuroimaging

Antibodies

Antibodies used	Anti-HSP90 Rabbit pAb, P07900, P82995, P07901, diluted to 1:1000;<br>Anti-HSP60 Rabbit pAb, P10809, P63039, P63038, diluted to 1:500;<br>Anti-Integrin α4 Rabbit pAb, p13612, diluted to 1:1000;<br>Anti-Integrin β1 Rabbit pAb, P09055, P49134, diluted to 1:1000;<br>Cy3-labeled goat anti-rabbit IgG, FITC-labeled goat anti-rabbit IgG, diluted to 1:200;<br>All the above were obtained from Wuhan service Biotechnology Co., Ltd.
Validation	All information about antibodies can be found on the respective antibody website:<br>Anti-HSP90 Rabbit pAb, P07900, P82995, P07901, diluted to 1:1000, https://www.servicebio.cn/goodsdetail?id=1506<br>Anti-HSP60 Rabbit pAb, P10809, P63039, P63038, diluted to 1:500, https://www.servicebio.cn/goodsdetail?id=5537<br>Anti-Integrin α4 Rabbit pAb, p13612, diluted to 1:1000, https://www.servicebio.cn/goodsdetail?id=746<br>Anti-Integrin β1 Rabbit pAb, P09055, P49134, diluted to 1:1000, https://www.servicebio.cn/goodsdetail?id=2618<br>Cy3-labeled goat anti-rabbit IgG, FITC-labeled goat anti-rabbit IgG, diluted to 1:200,https://www.servicebio.cn/goodsdetail?id=253;https://www.servicebio.cn/goodsdetail?id=259

Eukaryotic cell lines

Policy information about cell lines

Cell line source(s)	4T1 and C26 cell line was obtained from China Center for Type Culture Collection (Wuhan, China), Luc-4T1 cell line was

March 2021

Cell line source(s)	obtained from Wuhan service Biotechnology Co., Ltd., RAW 264.7 cell line was was kindly provided by Dr. Deqiang Deng (Britton Chance Center for Biomedical Photonics at Wuhan National Laboratory for Optoelectronics).
Authentication	Each cell line was morphologically confirmed according to the information provided by the cell-source center, and the main 4T1 and RAW 264.7 cell line were authenticated with short tandem repeat (STR) analysis.
Mycoplasma contamination	All cell lines were confirmed to be mycoplasma-negative by DAPI staining.
Commonly misidentified lines (See ICLAC register)	4T1 and RAW 264.7 cell lines were of stable origin and there was little possibility of misidentification.

Animals and other organisms

Policy information about studies involving animals; ARRIVE guidelines recommended for reporting animal research

Laboratory animals	BALB/c female nude mice (purchased as 4-week-old, used as 5-week-old, ~18 g), BALB/c female mice(purchased as 4-week-old, used as 5-week-old, ~18 g), KM male mice (purchased as 5-week-old, used as 6-week-old, ~32 g) were purchased from Beijing Weitong Lihua Biotechnology Co., Ltd. All mice were housed in an animal facility under constant environmental conditions (room temperature, 22±1°C, relative humidity, 40-70 % and a 12 h light-dark cycle). All mice had access to food and water ad libitum.
Wild animals	The study did not involve wild animals.
Field-collected samples	The study did not involve samples collected from the field.
Ethics oversight	All animal experiments were approved by the animal experiment ethics committee of Huazhong University of Science and Technology (IACUC Number: S904).

Note that full information on the approval of the study protocol must also be provided in the manuscript.

Flow Cytometry

Plots

Confirm that:

☒ The axis labels state the marker and fluorochrome used (e.g. CD4-FITC).

☒ The axis scales are clearly visible. Include numbers along axes only for bottom left plot of group (a 'group' is an analysis of identical markers).

☒ All plots are contour plots with outliers or pseudocolor plots.

☒ A numerical value for number of cells or percentage (with statistics) is provided.

Methodology

Sample preparation	4T1 breast cancer cells were derived from mice, and the cells were cultured in a 5% CO2 incubator at 37 °C for 12 h before used.
Instrument	CytoFLEX flow cytometer
Software	CytExpert (ver. 2.3.0.84) was used to collect and analyze the flow cytometry data.
Cell population abundance	After the cells were centrifuged, the pelleted cells were taken for flow cytometric analysis. With adjusting the gain and compensation, 10,000 cells were taken from each group for analysis.
Gating strategy	The untreated cell group was taken, and the voltage was adjusted to make the intensity of FTIC and PI channels around 1000; the single-stained cell group was taken for compensation adjustment and deducting the mutual influence, finally experimental groups were carry out. If the intensity value was greater than 1000, positive signal was considered to exist.

☒ Tick this box to confirm that a figure exemplifying the gating strategy is provided in the Supplementary Information.
