# Supplementary material for: Biomimetic material degradation for synergistic enhanced therapy by regulating endogenous energy metabolism imaging under hypothermia
Source: Nat Commun. 2022 Aug 5;13:4567. doi: 10.1038/s41467-022-32349-2 (PMC9355994; doi:10.1038/s41467-022-32349-2)
Supplement: Supplementary file 1 — Peer review file [file 41467_2022_32349_MOESM1_ESM.pdf]

# Peer Review File

## Biomimetic-Materials Degradation for Synergistic Enhanced Photothermal-Gas-Starving Therapy through Regulating Endogenous Energy-Metabolism Fluorescence Imaging under Deep-Hypothermia

**Open Access** This file is licensed under a Creative Commons Attribution 4.0 International License, which permits use, sharing, adaptation, distribution and reproduction in any medium or format, as long as you give appropriate credit to the original author(s) and the source, provide a link to the Creative Commons license, and indicate if changes were made. In the cases where the authors are anonymous, such as is the case for the reports of anonymous peer reviewers, author attribution should be to 'Anonymous Referee' followed by a clear attribution to the source work. The images or other third party material in this file are included in the article's Creative Commons license, unless indicated otherwise in a credit line to the material. If material is not included in the article's Creative Commons license and your intended use is not permitted by statutory regulation or exceeds the permitted use, you will need to obtain permission directly from the copyright holder. To view a copy of this license, visit http://creativecommons.org/licenses/by/4.0/.

REVIEWER COMMENTS

Reviewer #1 (Remarks to the Author): Expert in surface engineered nanoparticles

Cheng et al. prepared a biomimetic multifunctional nano-platform combining several factors, including Co-based MOF-polydopamine heterostructure, anethole trithione (ADT), and macrophage membrane for inhibiting the metastasis. The nano platform is equipped with photothermal-gas-starving therapeutic effects. But this complex combined formulation design profile is not very novel and additional evidence is needed to support the conclusions.

1. About Co-MOF, there are no physiological characteristics provided.

2. In Figure 1C, the cell membrane coating is not obvious. It is recommended to confirm this by immunogold staining TEM imaging.

3. The release profiles of the contents, including Co2+ and ADT, are not provided. Especially, ADT is related to the concentration of H2S gas in tumor cells.

4. It is mentioned in the Introduction that "H2S is equipped with two distinct physiological effects: pro-apoptosis at high concentration and growth-promoting at a low concentration". What is the specific range representing high or low concentration respectively? And does the H2S concentration generated in tumor cells assisted by the nano platform locate in the scope of high concentration?

5. In Figure 3H, it is recommended to prove the lysosomes escape by suppling the images showing that the fluorescence of Dil-labeled PCoA@M and lysosomes changed from totally merge to separate gradually.

Reviewer #2 (Remarks to the Author): Expert in nanoparticles and glycolysis

In the current study, the authors have developed a new biomimetic functional nano-platform with macrophage membrane for tumor metastasis, combined PDA and ADT in Co-MOF. It had multiple functions to inhibit the tumor, like photothermal thermal, high concentrations of H2S, inhibition of NADH, but why we use all this "cocktail" treatment on tumor metastasis, the author did not seem to give a suitable reason to answer from a clinical point of view (lung metastasis of breast cancer), and did not proposed a suitable design concept for tumor lung metastasis. Although this study is an interesting work, unfortunately I don't believe it good enough to publish in Nature Communications.

Several issues list as follow

1. Line 1, "attaention" should be replaced by "attention";

2. The author declare Co-MOF as MOF, but all through the paper there weren't any evidence to support this, is there any TEM micrographs with higher magnification or BET data to show its frame hole and heterostructure?

3. Line 383, "Compared with the P@M+NIR group, a large amount of red fluorescence appeared in the 4T1 cells of PCo@M+NIR group, maybe because the degradation of Co-MOF led to the large release of Co2+, and down-regulated the expression of heat shock protein, which, in turn enhanced the PTT effect and prompted large numbers of cell apoptosis." I am just curious about why the other content of Co-MOF can't be the reason to down-regulate the expression of heat shock protein. In addition, the released Co2+ played an important role in the whole process, then the release kinetic of Co2+ should be added to support this statement.

4. The images of the tumors should be put in the figure to support figure7's study.

5. The images of fluorescence were dark, please make it brighter to ensure clear pictures.

6. Line 509, the authors stated that " In comparison, P@M+NIR and PCo@M+NIR groups displayed different degrees of vacuolization, cell necrosis, atrophy, and separation of nuclear and cytoplasmic." However, there is no result to illustrate or support this finding.

7. It's difficult to find clear pictures of the positive biomarkers in figure7D and figure7E. So it's necessary to get some more typical views under a high-scale microscope.

8. Line 610, the authors stated that " Because normal lung tissue contains a large amount of dense myofilament actin, α-SMA fluorescent staining can well reflect this situation." Several evidences suggested that tumor tissues can get a great α-SMA fluorescent staining. Please consider whether this indicator is a reasonable choice.

Reviewer #3 (Remarks to the Author): Expert in nanoparticles

The authors of this paper designed a macrophage membrane nanoplatform grown on PDA as the core and co MOF as the shell layer to load H2S releasing precursor trithioanisine (ADT) into the pores of the MOF and the interstices of the heterostructure, and finally modified the macrophage membrane with a series of modifications. However, conceptual innovation is rather less. This is just good-considered combination of pre-existing systems and idea. It includes possible aspect into one system and nothing more. This work well perform on (i) materials characterization and (ii) biomedical effects. However, scientific resonation between these data, innovative concept, and surprising finding beyond expectable effects upon the combination are not well included. Therefore, insights in nanosciece and nanotechnology are not well included although it gives comprehensive results on materials for biomedical applications. There are no serious comparisons over huge numbers of related research works in the past literatures. I do not think that Nature Communications is suitable medium for this work.

**Reviewer #1** (Remarks to the Author): Expert in surface engineered nanoparticles

Cheng et al. prepared a biomimetic multifunctional nano-platform combining several factors, including Co-based MOF-polydopamine heterostructure, anethole trithione (ADT), and macrophage membrane for inhibiting the metastasis. The nano platform is equipped with photothermal-gas-starving therapeutic effects. But this complex combined formulation design profile is not very novel and additional evidence is needed to support the conclusions.

1. About Co-MOF, there are no physiological characteristics provided.

Reply: Thanks for your suggestion. In addition to the existing results, we added some new experiments to enhance physiological characteristics of Co-MOF. Firstly, for PDA, PCo (PDA@Co-MOF), PCoA (PDA@Co-MOF/ADT), and PCoA@M (PDA@Co-MOF/ADT@membrane), X-ray photoelectron spectroscopy analysis was performed to detect the composition and chemical state of the surface elements. It is well known that the characteristic element N exists in PDA, Co element exists in the cobalt-based MOF, S element exists in the prodrug ADT, and P element exists in the cell membrane. For PDA, it showed that signal peaks of elements such as C and N appeared (Figure S5), and the characteristic peak of N 1s was located at 400.4 eV. For PCo, the results exhibited that signal peaks of elements such as C, N, and Co emerged (Figure 1j). The characteristic main peak of N 1s was located at 399.03 eV (Figure S6), and four characteristic peaks belonged to Co 2p, which were Co $2p_{3/2}$ (780.73 eV & 786.68 eV) and Co $2p_{1/2}$ (796.73 eV & 802.63 eV), respectively. For PCoA, signal peaks of elements such as C, N, Co, and S occurred (Figure S7). Among them, the characteristic main peak of N 1s was located at 398.933 eV (Figure S7b), and Co 2p possessed four characteristic peaks: Co $2p_{3/2}$ at 780.983 eV & 786.833 eV, and Co $2p_{1/2}$ at 796.583 eV & 802.283 eV (Figure S7c). Additionally, the small peaks at 165.0 eV belonged to S 2p (Figure S7d). For PCoA@M, signal peaks of elements such as C, N, Co, S, and P appeared (Figure 1j). Similarly, the characteristic main peak of N 1s was located at 399.35 eV (Figure S8a); Co 2p had four characteristic peaks, where 781.05 eV and 787.35 eV belonged to Co $2p_{3/2}$, 797.25 eV and 802.70 eV belonged to Co $2p_{1/2}$ (Figure S8b); the peak at 165.5 eV belonged to S 2p (Figure S8c) and the characteristic peak of P 2p was located at 133.45 eV (Figure S8d). From the above results, there were relatively weak signal peaks of Co and S in PCoA@M, which was because the maximum depth of detection by XPS was about 10 nm, and in PCOA@M, the thickness of the cell membrane was about 8 nm, which would lead to the poor detection sensitivity of the cobalt-based MOF chelated on the surface of PDA and the loaded pre-drug ADT. In summary, The XPS results fully verified the cobalt-based MOF, the heterostructuring process of cobalt-based MOF

on the PDA surface, and the successful synthesis of PCoA@M. This section was added in lines 10-31, page 7, and lines 1-3, page 8.

Secondly, the process of the cobalt-based MOF coating on the surface of PDA was investigated under different reaction conditions. The results showed that when the dosage of PDA was 0.5, 0.6, 0.7, 0.8, and 1 mg, the imidazole was 60 mg, and the rotation speed was 420 rpm, the amount of free hexagonal cobalt-based MOF was continuously decreased along with the increase of PDA dosage (Figure S1). When the dosage reached to 1 mg, there was almost no free cobalt-based MOF, indicating that MOF was mostly chelated on the surface of PDA, which proved the synthesis of heterostructure. This section was added in lines 14-20, page 6.

Finally, the characteristic elemental mapping analysis was carried out for PCo and PCoA@M. It was clear that PCo exhibited a well-proportioned distribution of N and Co elements (Figure S3 and Figure S4), and relatively obvious N, Co, S, and P signal were in PCoA@M nanoparticles (Figure 1h and 1i). This also fully explained the formation of the cobalt-based MOF on the surface of PDA and the functional modification of cell membranes. This section was added in lines 5-10, page 7.

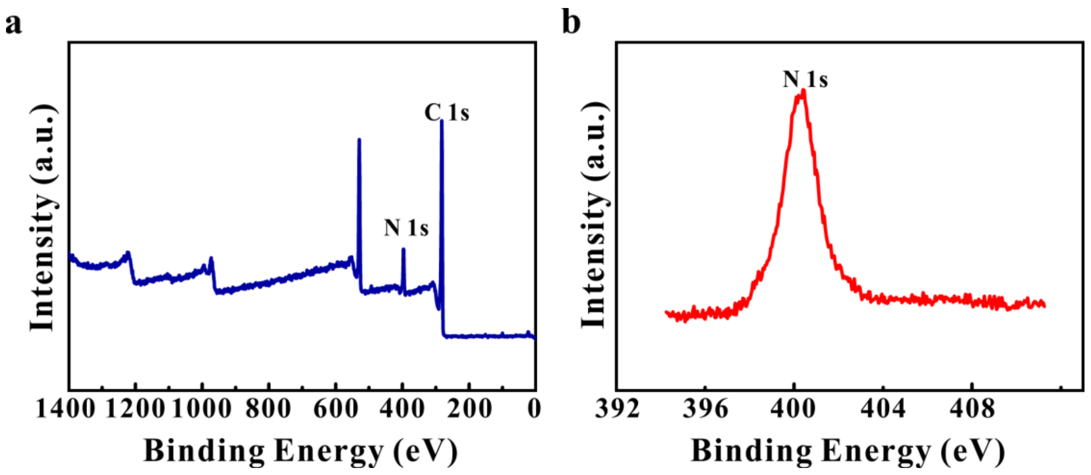


**Figure S5.** X-ray photoelectron spectroscopy of PDA (A) and high-resolution XPS spectra of N (B).

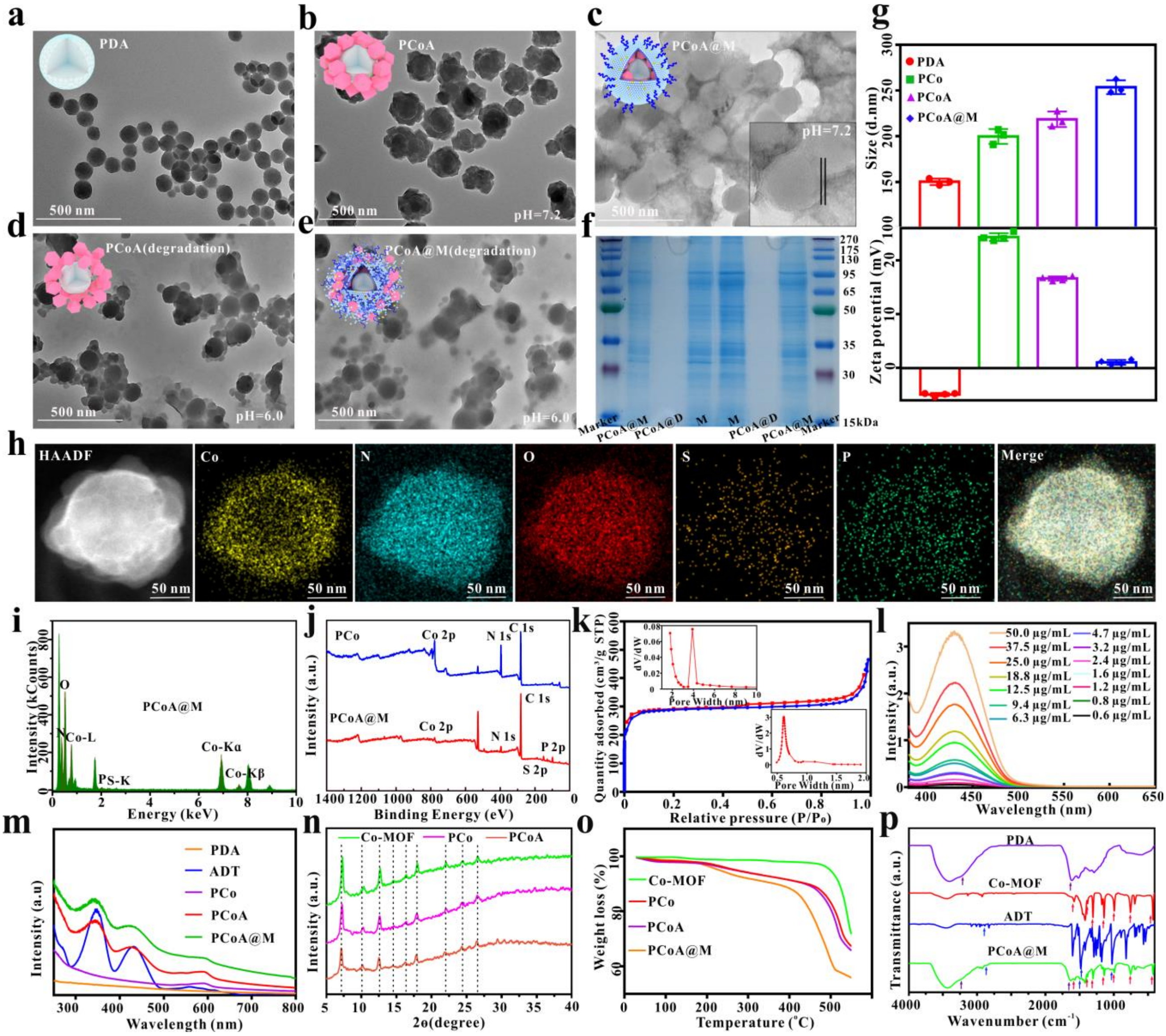

**Figure 1.** TEMs of PDA (a), PCoA (b) and PCoA@M (c) at pH=7.2; PCoA (d) and PCoA@M (e) at pH=6.0; SDS-PAGE protein analysis of macrophage membrane, PCoA@M and PCoA@D (f); the hydrated particle sizes, and zeta potentials of PDA, PCo, PCoA and PCoA@M (g); elemental mapping (h) and energy dispersive spectrometer (i) of PCoA@M; X-ray photoelectron spectroscopy of PCo and PCoA@M (j); nitrogen adsorption-desorption isothermal curve of PCo (k), inset: pore size distribution; absorption spectra of different concentrations of ADT (l); absorption spectra of PDA, PCo, PCoA and PCoA@M (m); X-ray powder diffractions of Co-MOF, PCo and PCoA (n); thermogravimetric analysis of Co-MOF, PCo, PCoA and PCoA@M (o); infrared spectra of PDA, Co-MOF, ADT and PCoA@M (p).

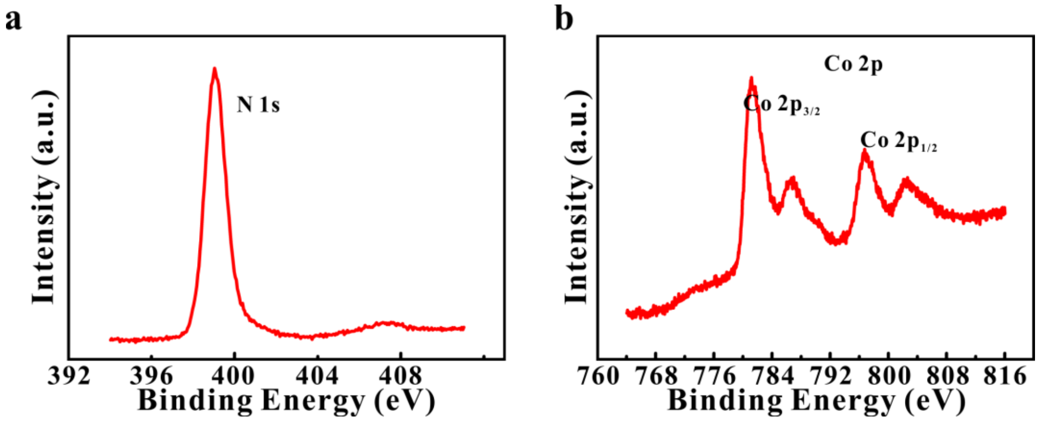


**Figure S6.** High-resolution XPS spectra of N (a) and Co (b) in PCo.

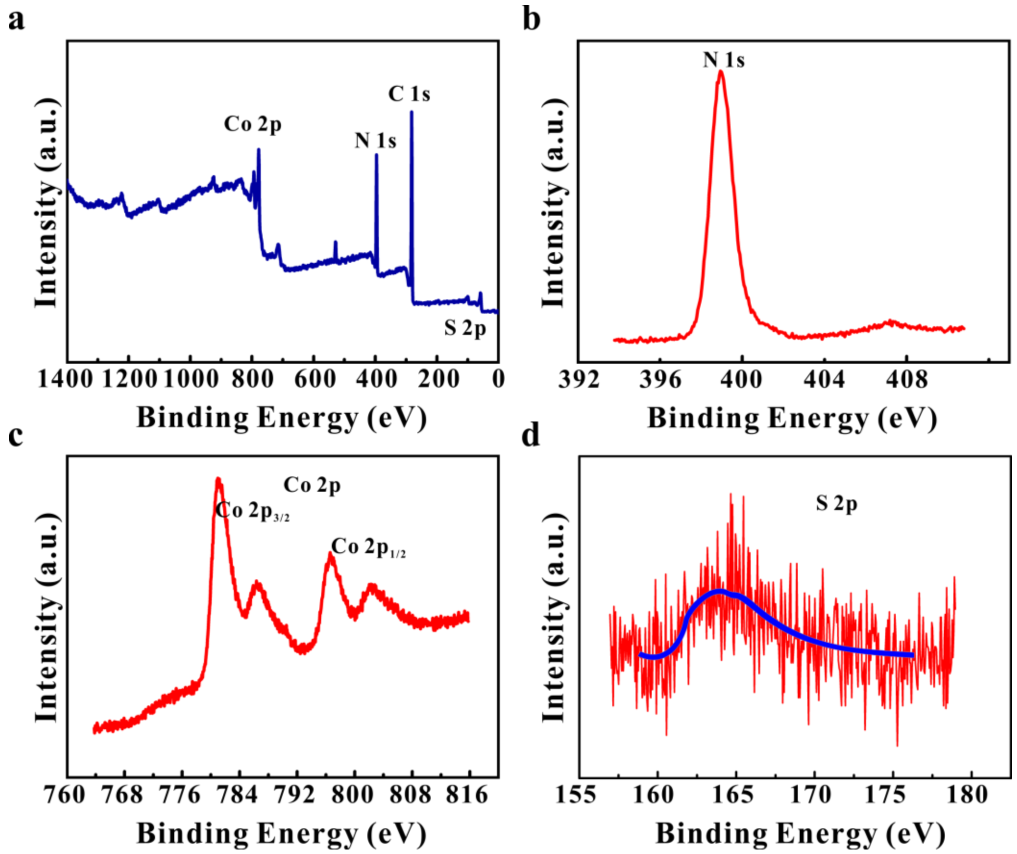


**Figure S7.** X-ray photoelectron spectroscopy of PCoA (a) and and high-resolution XPS spectra of N (b), Co (c), and S (d), respectively.

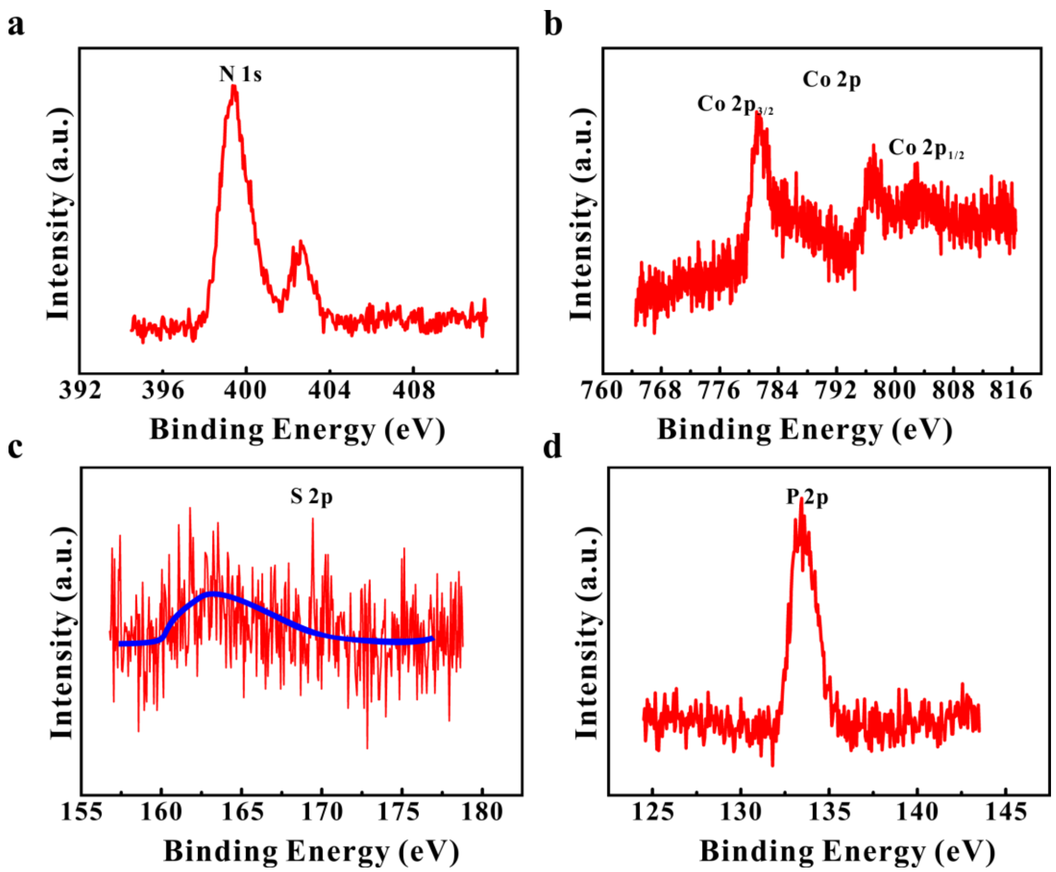


**Figure S8.** High-resolution XPS spectra of N (a), Co (b), S (c), and P (d) in PCoA@M.

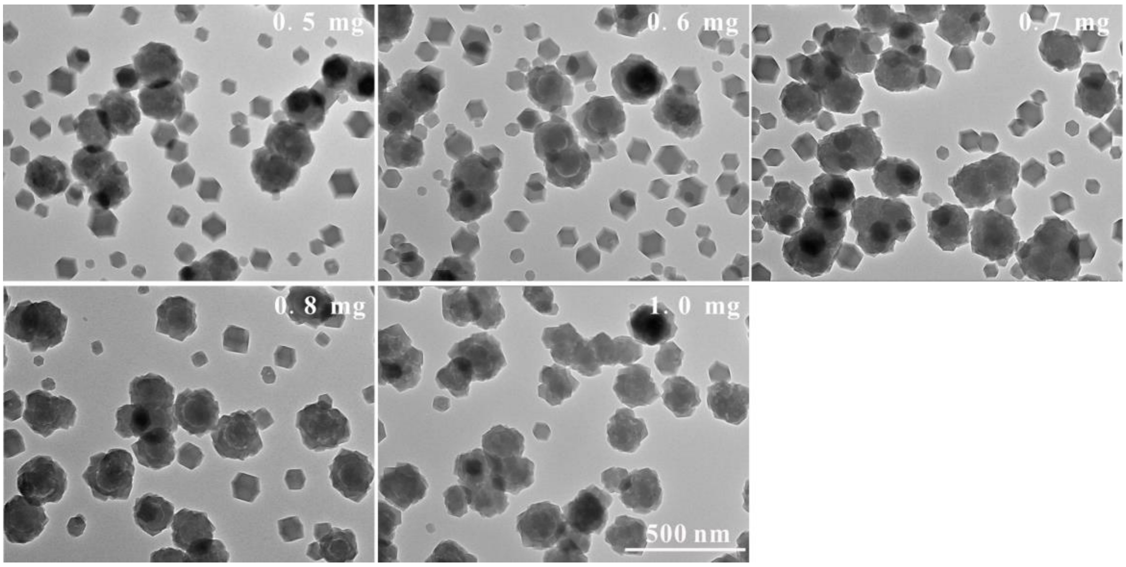


**Figure S1.** TEM of heterostructure formation under different PDA dosage.

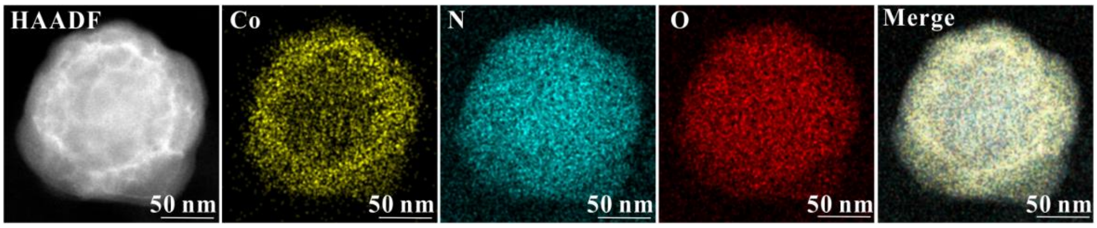


**Figure S3.** High angle annular dark field (HAADF) imaging and elemental mapping of PCo.

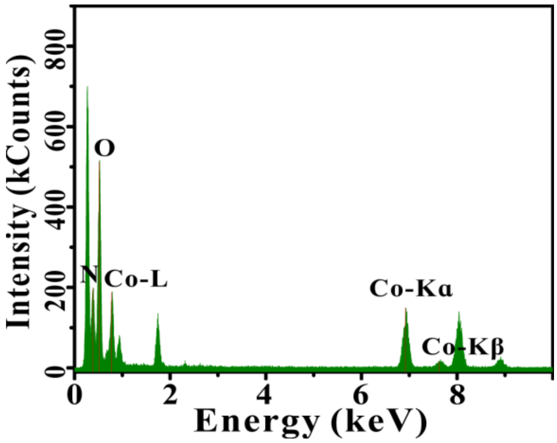


**Figure S4.** Energy dispersive spectroscopy analysis of PCo.

2. In Figure 1C, the cell membrane coating is not obvious. It is recommended to confirm this by immunogold staining TEM imaging.

Reply: Thanks for your suggestion. We added such experiments according to the advice. Firstly, for cell membrane coating, PCoA@M was negatively stained with 2% ammonium molybdate under the condition that the pH was adjusted to 7.4 with ammonium acetate. The TEM results showed that the structure of the cell membrane could be well highlighted (Figure 1c), which presented as a white circle, indicating the successful synthesis of the cell membrane functionalized probe. This section was added in lines 14-16, page 6.

Secondly, the XPS results exhibited that there were characteristic peaks of N, Co, S, and P in PCoA@M (Figure 1j), while only characteristic peaks of N, Co, and S in PCoA (Figure S7), and the difference between PCoA and PCoA@M lied in the presence of cell membrane wrapping, while P was a characteristic element in the cell membrane, so the XPS results further demonstrated that the cell membrane was successfully wrapped on the heterostructure. This section was added in lines 10-31, page 7, and lines 1-3, page 8.

Finally, high-resolution elemental mapping detection of PCoA@M (Figure 1h) was performed, and similarly, the appearance of P element mapping also indicated the functional coating of the cell

membrane. Therefore, according to the above three methods, the formation of cell membrane-functionalized heterostructure was verified. This section was added in lines 5-10, page 7. New Figure 1 and Figure S7 were shown in Reply to Question 1.

3. The release profiles of the contents, including $Co^{2+}$ and ADT, are not provided. Especially, ADT is related to the concentration of $H_2S$ gas in tumor cells.

Reply: Thanks for your suggestion. We added such experiments according to the advice. To study the release of cobalt ion, the detection of Co element was carried out by atomic absorption spectrophotometer. PCoA@M (3 mg) was fixed with a dialysis bag and placed in a beaker containing 80 mL of PBS separately, and divided into (1) pH 5.5, (2) pH 6.0, and (3) pH 7.2 groups. At 10 min, 1, 2, 4, 8, 12, 24, 72, and 96 h, 200 μL of the solution was taken out for detection. The results showed that when the pH was 5.5, the release of cobalt ion increased with time (Figure 4a), and reaching to 55.42% of the drug loading at 12 h; when the pH was 6.0, the release trend of cobalt ion was basically the same with pH 5.5, but the release amount (48.15%) was slightly smaller at the same time point; while the pH was 7.2, the release amount of cobalt ion changed little along with time, and 2.63% at 12 h, which was much lower than those of pH 5.5 and pH 6.0. The above results fully demonstrated that cobalt-based MOF possessed low pH responsiveness, and the lower the pH, the faster the degradation rate of cobalt-based MOF. This section was added in lines 11-22, page 18. The release of ADT depended on the degradation of MOF, so ADT release experiment at pH=6.0 was performed based on the above results of MOF degradation. 9 aliquots of PCoA@M were separately incubated in 200 μL of water (pH 6.0) for 10 min, 1, 2, 4, 8, 12, 24, 72, and 96 h, then dissolved and centrifuged in 1 mL of methanol. The supernatant was taken out to measure the ADT absorption spectrum with a UV-Vis spectrophotometer. The results showed that under the condition of pH 6.0, ADT started to release at 10 min (Figure 4b and S26), gradually increased with time, and finally stabilized after 72 h. This section was added in lines 22-28, page 18.

To study the release of $H_2S$ in PCoA@M, a qualitative experiment of the induced release was proceeded by tris (2-carboxyethyl) phosphine (TCEP). It was known that TCEP could induce the rupture of disulfide bond in ADT and release $H_2S$, which chemically reacted with lead acetate to form black lead sulfide precipitate. Results showed that after 3 h reaction at room temperature, lead acetate-impregnated filter paper in PCoA and PCoA@M groups appeared black in color compared

with PDA and PCo groups (Figure S28), indicating that the released $H_2S$ from PCoA and PCoA@M chemically reacted with lead acetate to generate black PbS, it proved that PCoA@M could release $H_2S$ in a very responsive manner.

Washington State Probe-1 (WSP-1) could react with $H_2S$ selectively and rapidly to generate benzodithiolone and another fluorescent compound ($E_x$=465nm, $E_m$=515nm). Subsequently, the fluorescence detection analysis of $H_2S$ was performed based on fluorescence-on/off reaction strategy. When the $H_2S$ solution was co-incubated with WSP-1, the fluorescence signal of that fluorescent compound increased with the prolongation of the incubation time within 2 h (Figure S29), and the intensity of fluorescence signal held steady when the $H_2S$ solution was incubated with WSP-1 for 4 h, so the optimal time for the reaction of $H_2S$ with WSP-1 was 2~4 h. This section was added in lines 15-21, page 19.

Next, PCoA@M and WSP-1 were co-incubated with 4T1 cells for different times (10, 30 min, 1, and 4 h). The results showed that with the increase of incubation time, the green fluorescence area expanded continuously due to the reaction of WSP-1 (Figure 4d), and its intensity also increased, reaching to the maximum at 4 h, which was basically consistent with the results of fluorescence detection in vitro. This also showed that with the co-incubation of PCoA@M and 4T1 cells, the ADT pro-drug released hydrogen sulfide, and its released amount continued to increase with the incubation time, which then led to a large amount of fluorescent product from reaction of $H_2S$ and WSP-1. This section was added in lines 21-28, page 19.

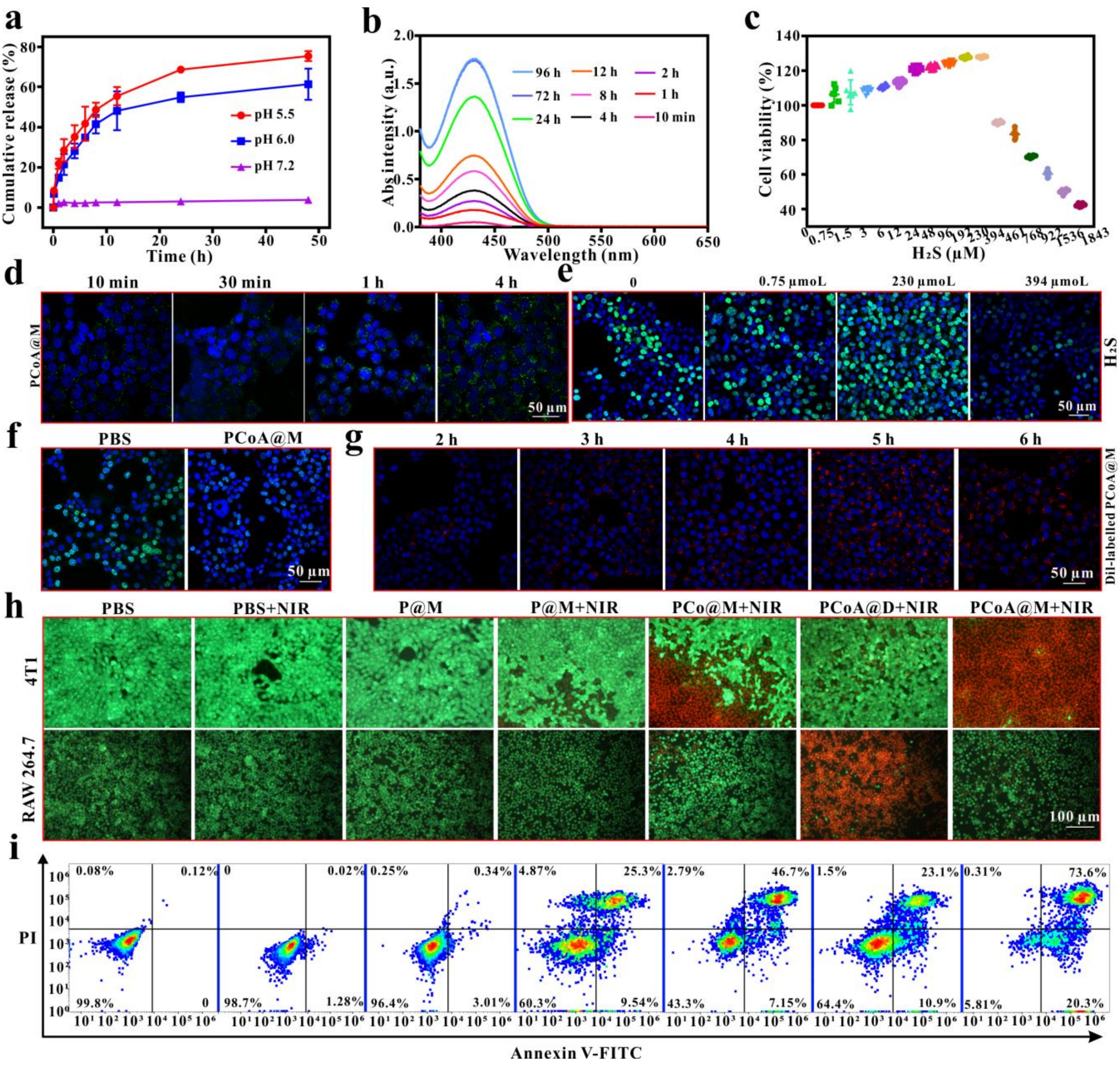


**Figure 4.** Cumulative release of cobalt ion at different times and pH (a); absorption spectra of ADT drugs released at different time points (b); CCK-8 cytotoxicities of 4T1 cells incubated with different concentrations of $H_2S$ (c); confocal imaging of 4T1 cells after incubation with PCoA@M+WSP-1 for different times (d); EDU proliferation of 4T1 cells co-incubated with different concentrations of $H_2S$ (e); EDU proliferation of 4T1 cells co-incubated with PBS and PCoA@M (f); confocal imaging of 4T1 cells after incubation with Dil-labeled PCoA@M for different times (g); fluorescence imaging of calcein/PI staining 4T1 and RAW 264.7 cells incubated with different probes under different conditions (h); flow cytometry analysis of 4T1 cells' apoptosis under above different conditions (i).

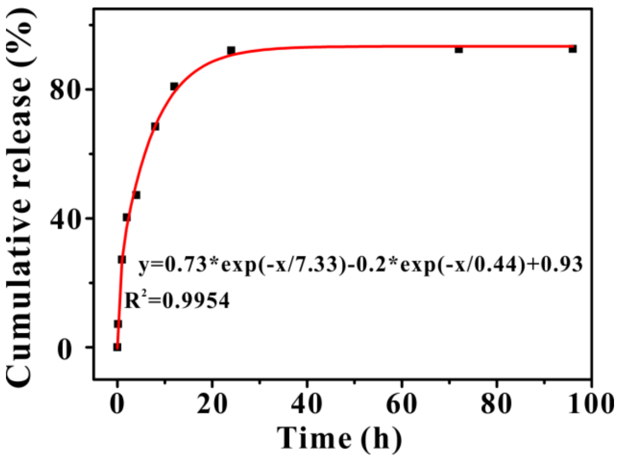


**Figure S26.** Cumulative release of ADT at pH 6.0.

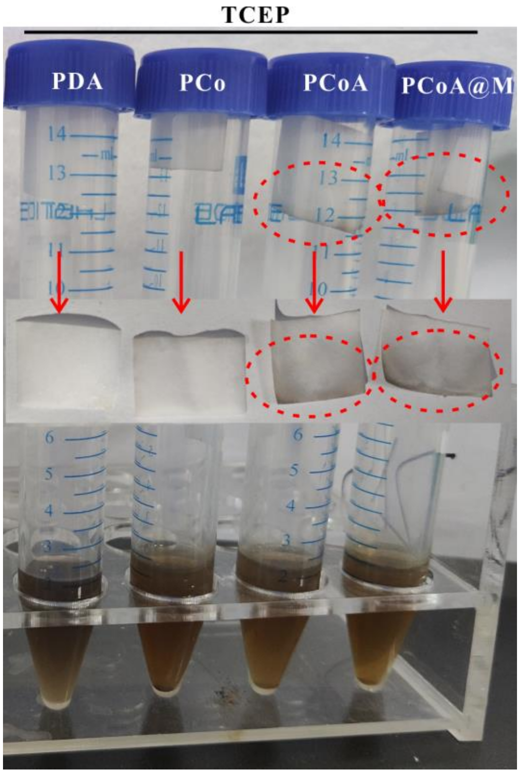


**Figure S28.** $H_2S$ released from incubation of different probes with TCEP for 3 h under acidic condition and color reaction with lead acetate-impregnated filter paper.

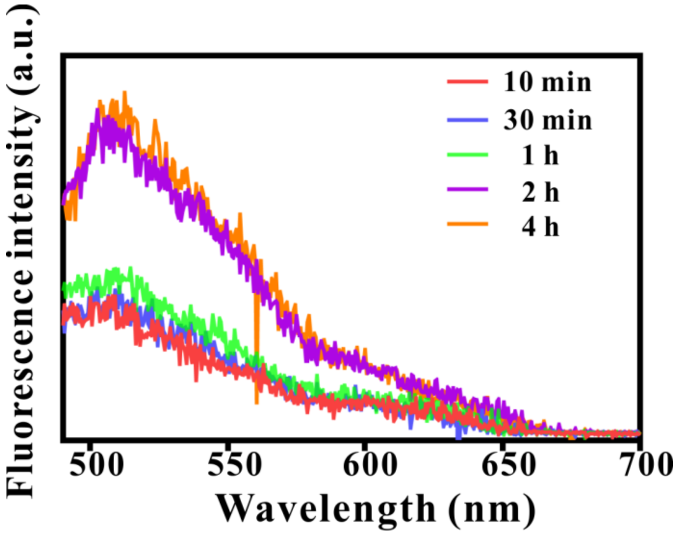


**Figure S29.** Fluorescence emission spectra after WSP-1 incubated with $H_2S$ for different times.

4. It is mentioned in the Introduction that "$H_2S$ is equipped with two distinct physiological effects: pro-apoptosis at high concentration and growth-promoting at a low concentration". What is the specific range representing high or low concentration respectively? And does the $H_2S$ concentration generated in tumor cells assisted by the nano platform locate in the scope of high concentration?

Reply: Thanks for your suggestion. To verify this view "low concentration promote cell growth and high concentration inhibit cell growth", standardized CCK-8 cytotoxicity experiments with different concentrations of $H_2S$ were performed by incubating different concentrations of $H_2S$ (1843, 1536, 922, 768, 461, 394, 230, 192, 96, 48, 24, 12, 6, 3, 1.5, 0.75, and 0 μM) with 4T1 and C26 cells. The results exhibited that for 4T1 cells, when the concentration of $H_2S$ was less than 230 μM, the cell viability increased with the rise of $H_2S$ concentration (Figure 4c), but when the concentration of $H_2S$ was higher than 230 μM, the cell viability decreased. For C26 cells, when the concentration of $H_2S$ was less than 394 μM, the cell viability increased with the rise of $H_2S$ concentration; while the $H_2S$ concentration was higher than 394 μM, the cell viability decreased along with the $H_2S$ concentration (Figure S27). This section was added in lines 29-31, page 18, and lines 1-6, page 19.

5-ethynyl-2-deoxyuridine (EDU) is a thymine nucleoside analog, whose acetylene group is rare in natural compounds and can be inserted into the replicating DNA molecules during cell proliferation. Therefore, based on the conjugation reaction of EDU with dye, cell proliferation can be efficiently and rapidly detected, and percentage of cells in S phase also can be effectively detected. Then, in order to study the growth promotion or inhibition of cells resulted from $H_2S$, EDU cell proliferation experiments were conducted. The results showed that when the concentration of $H_2S$ probe was 0,

the proliferating cells (green fluorescence) accounted for 41.98% of the total cells (Figure 4e); when the concentration of $H_2S$ probe was 0.75 μM, the proliferating cells accounted for 51.89% of the total cells, while 230 μM for 71.5%, and 394 μM for 29.24% of the total cells. The above results further illustrated that low concentrations of $H_2S$ would promote cell growth, and high concentrations of $H_2S$ would inhibit cell growth. This section was added in lines 5-15, page 20.

In order to investigate whether the released $H_2S$ from the PCoA@M probe would accumulate in the cell to reach a high concentration range, concentration of $H_2S$ was firstly calculated. When the PCoA@M probe was incubated at pH=6.0 for more than 8 h, the amount of released ADT was up to 68.5% at 8 h (Figure 4b and Figure S26), that was, the concentration of released $H_2S$ was about 953.9 μM. There was inherent $H_2S$ in 4T1 cells, so after the PCoA@M probe was incubated with the cells, the final concentration of $H_2S$ was more than 953.9 μM, which was higher than the lower limit of $H_2S$ concentration that inhibited cell growth. This section was added in lines 28-31, page 19, and lines 1-4, page 20. The inhibitory effect was further verified by the EDU cell proliferation experiment, that was, after the cells were co-incubated with PBS and PCoA@M, the proportion of proliferating cells was significantly lower than that of the PBS group (Figure 4f), which further proved that the released $H_2S$ by PCoA@M probe could significantly inhibit the proliferation and growth of 4T1 cells. This section was added in lines 15-19, page 20.

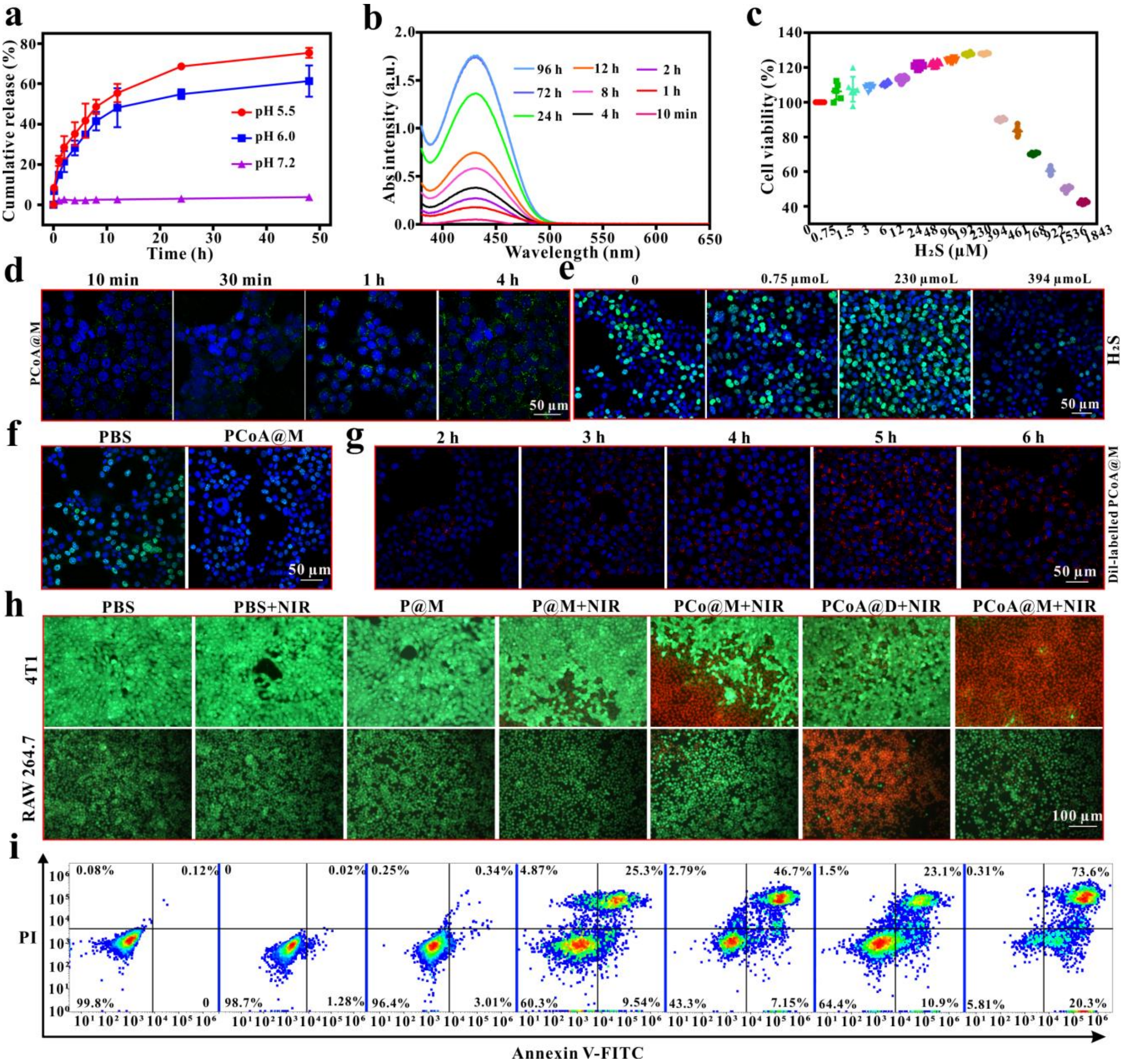


**Figure 4.** Cumulative release of cobalt ion at different times and pH (a); absorption spectra of ADT drugs released at different time points (b); CCK-8 cytotoxicities of 4T1 cells incubated with different concentrations of $H_2S$ (c); confocal imaging of 4T1 cells after incubation with PCoA@M+WSP-1 for different times (d); EDU proliferation of 4T1 cells co-incubated with different concentrations of $H_2S$ (e); EDU proliferation of 4T1 cells co-incubated with PBS and PCoA@M (f); confocal imaging of 4T1 cells after incubation with Dil-labeled PCoA@M for different times (g); fluorescence imaging of calcein/PI staining 4T1 and RAW 264.7 cells incubated with different probes under different conditions (h); flow cytometry analysis of 4T1 cells' apoptosis under above different conditions (i).

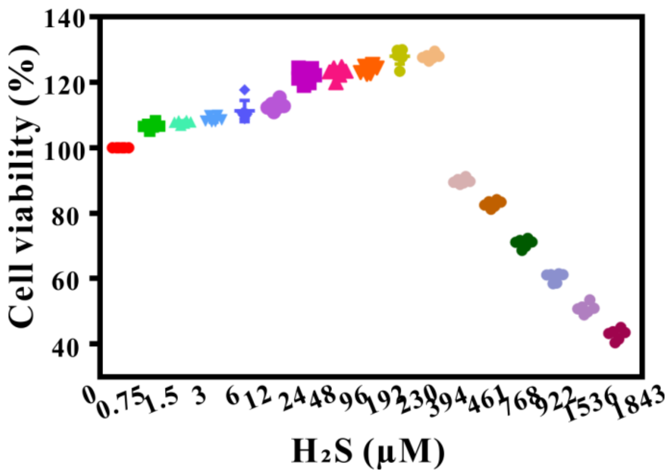


**Figure S27.** CCK-8 cytotoxicities of C26 cells incubated with different concentrations of $H_2S$ probes.

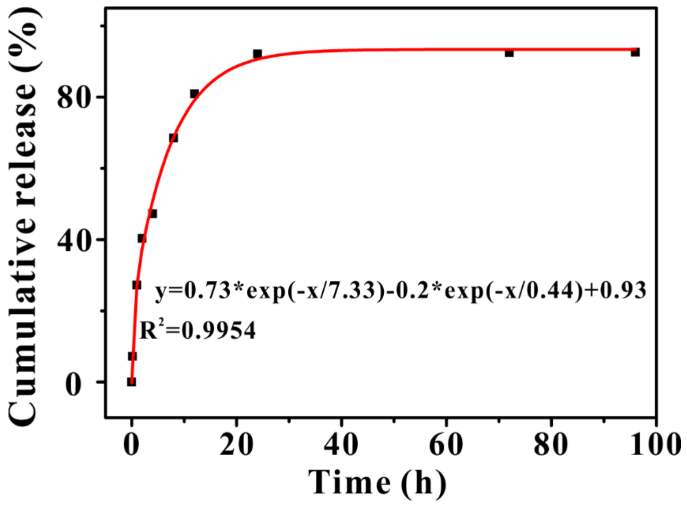


**Figure S26.** Cumulative release of ADT at pH 6.0.

5. In Figure 3H, it is recommended to prove the lysosomes escape by suppling the images showing that the fluorescence of Dil-labeled PCoA@M and lysosomes changed from totally merge to separate gradually.

Reply: Thanks for your suggestion. For verifying the lysosome escape of PCoA@M probe, lysosomal co-localization experiments were conducted by incubating the Dil-labeled PCoA@M probe with 4T1 cells for different times (2, 3, 5, 7, 9, 11, and 13 h). The results showed that when 4T1 cells were incubated with the Dil-labeled PCoA@M probe for 2 h, the red fluorescence was weak (Figure 3i), which might be due to less phagocytosis of the Dil-labeled PCoA@M probe by the cells. With the prolongation of time, the red fluorescence of the probe largely overlapped with the green fluorescence of lysosome at 5 h. However, as the incubation time of the probe prolonged, the

overlapping area of the red fluorescence from the Dil-labeled PCoA@M probe and the green fluorescence of lysosome gradually decreased. At 13 h, the red fluorescence of the probe and the green fluorescence of the lysosome were separated clearly, which further indicated that the lysosome escape ability of the Dil-labeled PCoA@M probe was realized along with the increasing of the incubation time of the probe and cells, all the above results were also demonstrated by quantitative analysis of fluorescence co-localization (Figure S24), where the intensity firstly approached and gradually become inconsistent with distance. This section was added in lines 28-31, page 17, and lines 1-10, page 18.

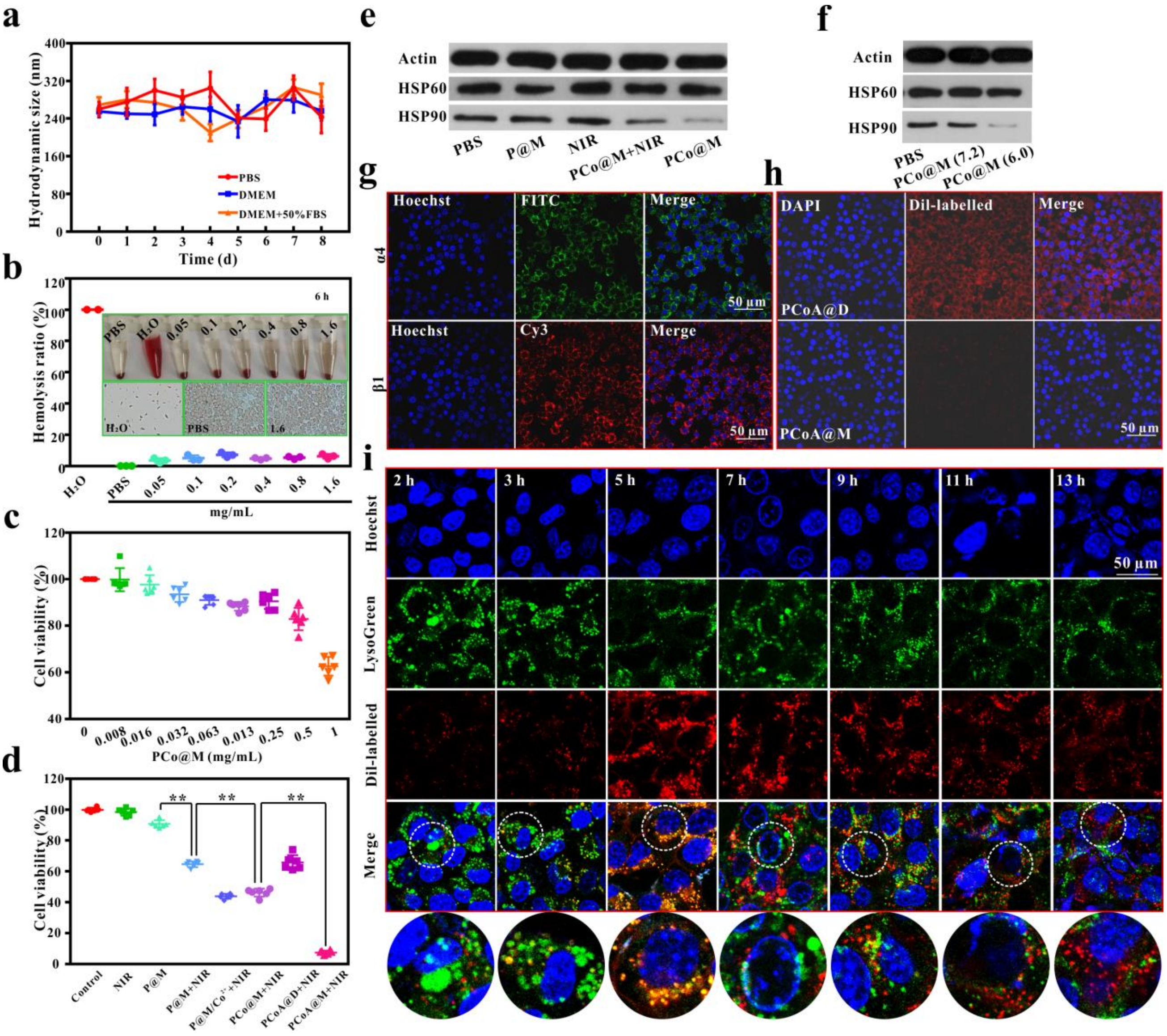


**Figure 3.** Hydrodynamic particle sizes of PCoA@M along with time in different media (a); the hemolysis rates of erythrocytes after incubation with different concentrations of probe, the inset was the bright-field image and the micrograph of the cell smear made by the precipitate after incubation (b); CCK-8 cytotoxicities of 4T1 cells incubated with different concentrations of PCo@M (c); survival rates of 4T1 cells incubated and treated with different probes, n=6, **: $p<0.01$ (d); west blot analysis of heat shock protein after different probe treatments of 4T1 cells (e) and treatments under different pH (f); immunofluorescence imaging for the detection of α4 and β1 antigens on the cell membrane of macrophages (g); confocal imaging of Dil-labeled PCoA@M and PCoA@D incubated with RAW264.7 for 5 h (h); Dil-labeled PCoA@M and 4T1 cells incubated for 2, 3, 5, 7, 9, 11, and 13 h and co-localized imaging (i).

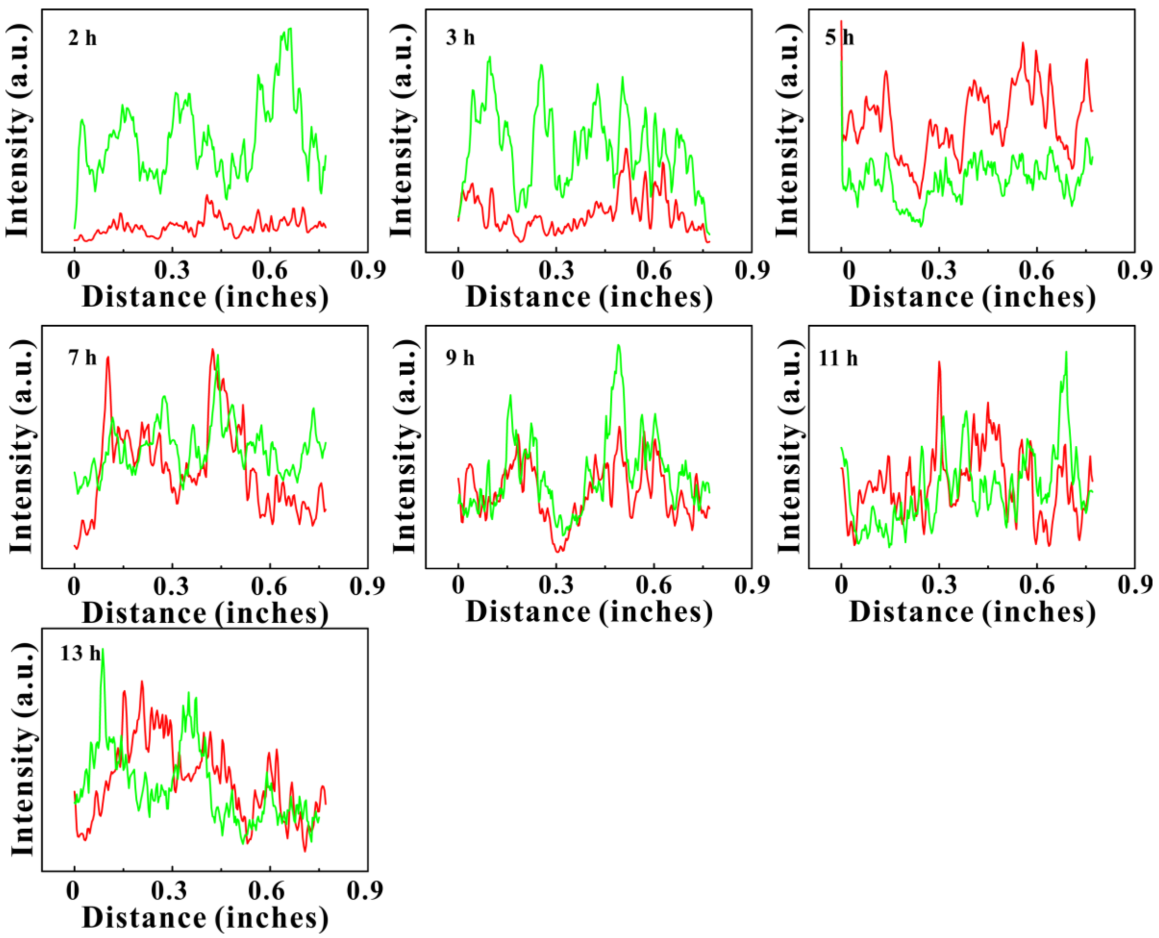


**Figure S24.** Quantitative analysis of fluorescence co-localization after Dil-labeled PCoA@M and 4T1 cells incubated for 2, 3, 5, 7, 9, 11 and 13 h and co-localized imaging.

**Reviewer #2** (Remarks to the Author): Expert in nanoparticles and glycolysis

In the current study, the authors have developed a new biomimetic functional nano-platform with macrophage membrane for tumor metastasis, combined PDA and ADT in Co-MOF. It had multiple functions to inhibit the tumor, like photothermal thermal, high concentrations of $H_2S$, inhibition of NADH, but why we use all this “cocktail” treatment on tumor metastasis, the author did not seem to give a suitable reason to answer from a clinical point of view (lung metastasis of breast cancer), and did not proposed a suitable design concept for tumor lung metastasis. Although this study is an interesting work, unfortunately I don't believe it good enough to publish in Nature Communications.

Reply: Thanks for your suggestion. It is well known that “cocktail” therapy is initially proposed and applied in the field of AIDS treatment, and its advantages lie in reducing the drug resistance caused by a single drug, maximally inhibiting the replication of the virus, partially or even fully recovering the damaged immune function, delaying the progression of the disease, extending patients' lives, and improving their quality of life. Now, the clinical drug treatment of tumor is based on NCCN (National Comprehensive Cancer Network) or CSCO (Chinese Society of Clinical Oncology) tumor treatment guidelines. First, the most suitable first-line drugs are selected, if poor tumor inhibition effect and drug resistance appear, second-line drugs and later-line drugs are used in combination. For the treatment of breast cancer, the commonly used first-line treatment is the combination of CDK4/6 inhibitors such as palbociclib with endocrine drugs. Clinical studies showed that the progression-free survival time of patients in the palbociclib combination group was 27.6 months. For the second- and later-line treatment, patients who progressed after first-line endocrine therapy and have not received CDK4/6 inhibitor therapy can choose CDK4/6 inhibitors such as palbociclib combined with fulvestrant or aromatase inhibitor. Here, the progression-free survival time of patients treated with palbociclib and fulvestrant was 46.7 months. According to the age of human and rat (a 12-month-old rat is equal to a thirty-year-old human), the first-line combination therapy can prolong the survival time of rats by 27.6 d, and the second-line and later-line treatment can prolong the survival time of rats by 46.7 d.

In this paper, tumor model and lung metastasis model were established by subcutaneous (Figure 7), orthotopic (Figure 9), and tail vein (Figure 10) injection, respectively. The subcutaneous injection method was to form a solid tumor area by subcutaneously injecting a certain concentration of tumor cells into the back of the mouse. In order to strictly protect animal welfare and comply with animal ethics standards, when the tumor volume reached to 1000 $mm^3$ or the mice died naturally, this time was defined as the death time of the mouse. When the tumor volume reached to about 100 $mm^3$,

different probes (PBS, P@M, P@M+NIR, PCoA@D+NIR, and PCoA@M+NIR) were injected. According to statistical data, the average death time of mice in the PBS group was 26 d (Figure 7k), while only one mouse in the PCoA@M+NIR group still developed tumor recurrence at 44 d (Figure 7o). Based on the growth rate of tumor recurrence, we calculated that it would take at least 26 d for the tumor recurrence mouse to reach a volume of 1000 $mm^3$, which would be considered to die, that was, the survival time of mice treated with PCoA@M+NIR was extended by at least 44 d (for the recurrence one), which was superior to the first-line combination drug of clinical breast cancer treatment, so we thought this probe was promise to be a supplement for the first-line drugs. By dissecting the organs of the mice at the sacrifice time, it was obvious that there was few metastatic nodule in the lungs of PBS and P@M groups (Figure 7h), which could not form effective lung metastasis of breast cancer, and no obvious metastatic nodule appeared in organs such as the heart, liver, spleen, and kidney (Figure S35-39). It also showed that although the subcutaneous injection method was convenient and simple to operate, the formation rate of tumor *in situ* was low, so there were many restrictions on modeling. Therefore, orthotopic injection of tumor cells into the second pair of mammary glands in mice was performed. When the tumor volume reached to 100 $mm^3$, different probes (PBS, P@M, PBS+NIR, P@M+NIR, PCo@M+NIR, PCoA@D+NIR, and PCoA@M+NIR) were injected. After 28 d of treatment, lung tissue was dissected out. It was obvious that significant increase in number of metastatic lung nodule occurred in PBS, P@M, and PBS+NIR groups (Figure 9g and h), which also indicated that the orthotopic injection method was easier to establish the lung metastatic tumor model than the subcutaneous injection method, and comparing with the other treatment groups, the PCoA@M+NIR group had significantly fewer lung metastatic nodule, implying that the PCoA@M probe produced a strong effect of metastatic inhibition on the orthotopic tumor. As we know, the key of tumor metastasis is that tumor cells in tumor tissue migrate into the blood, reaching to all the body through blood circulation, and form new cell implantation sites. The reason why breast cancer cells form more lung metastasis is due to short-term proximal metastases, that is, the lung is relatively close to the breast. So, to establish a lung metastasis model more quickly, Luc-4T1 cells were injected into mice through the tail vein. After 1, 2, and 9 d of treatment (Figure 10a), bioluminescence images on 16, 19, and 25 d of mice were performed, and for PCoA@M+NIR group, it was clear that no obvious metastatic tumor cell signal appeared in the lungs and other organs (Figure 10d), while other groups had a certain degree of metastatic tumor cell

signal, which fully exhibited that the PCoA@M probe possessed strong inhibitory and killing effects on metastatic cells. The mice were dissected after 25 d, and a large number of aggregated tumor cell populations were found in lungs of PBS group by HE staining (Figure 10e), which was significantly different from pulmonary fibrosis, and a large number of metastatic plaques were also present in the liver, while it was relatively few in the PCoA@M+NIR group. Furthermore, comparing with orthotopic injection, tail vein injection has a faster success rate of lung metastasis, and tumor metastasis in other organs can also be achieved. This may be because the direct injection of tumor cells into the tail vein will cause the tumor cells to fully form implantation in the organs after a long period of blood circulation. So this method is helpful for the exploration of metastatic tumor models such as lungs, liver, and other organs metastasis, and it is also a powerful mean to study the treatment of drugs on organ metastasis models. It is well known that when a large number of tumor cells aggregate to form tumor spheres, the cancer tends to reach an advanced stage, and most advanced cancers are incurable. Therefore, the purpose of tumor treatment is to control the tumor volume, relieve symptoms, and prolong survival on the basis of ensuring the life quality of patients.

The treatment of metastatic breast cancer is a complex process. Although there are many clinical research data on first- and second-line drugs and the regimens are relatively standardized, some limitations still exist in the recommended treatment plan for the later line due to the lack of high-level evidence-based medical evidence. Now, for seeking the optimal treatment strategy and optimal drug selection for advanced breast cancer (including dosing, dosing schedule, and markers for efficacy prediction), it is necessary to bring together all parties to jointly launch some well-designed and high-quality clinical trials. At the same time, the concept of multidisciplinary comprehensive treatment should be actively promoted, that is, a more precise and individualized comprehensive treatment plan is given to each patient, and the research results are widely extended to clinical practice, so as to optimize the treatment of patients with advanced breast cancer, and ultimately prolonging the survival time of patients and improving the life quality of patients.

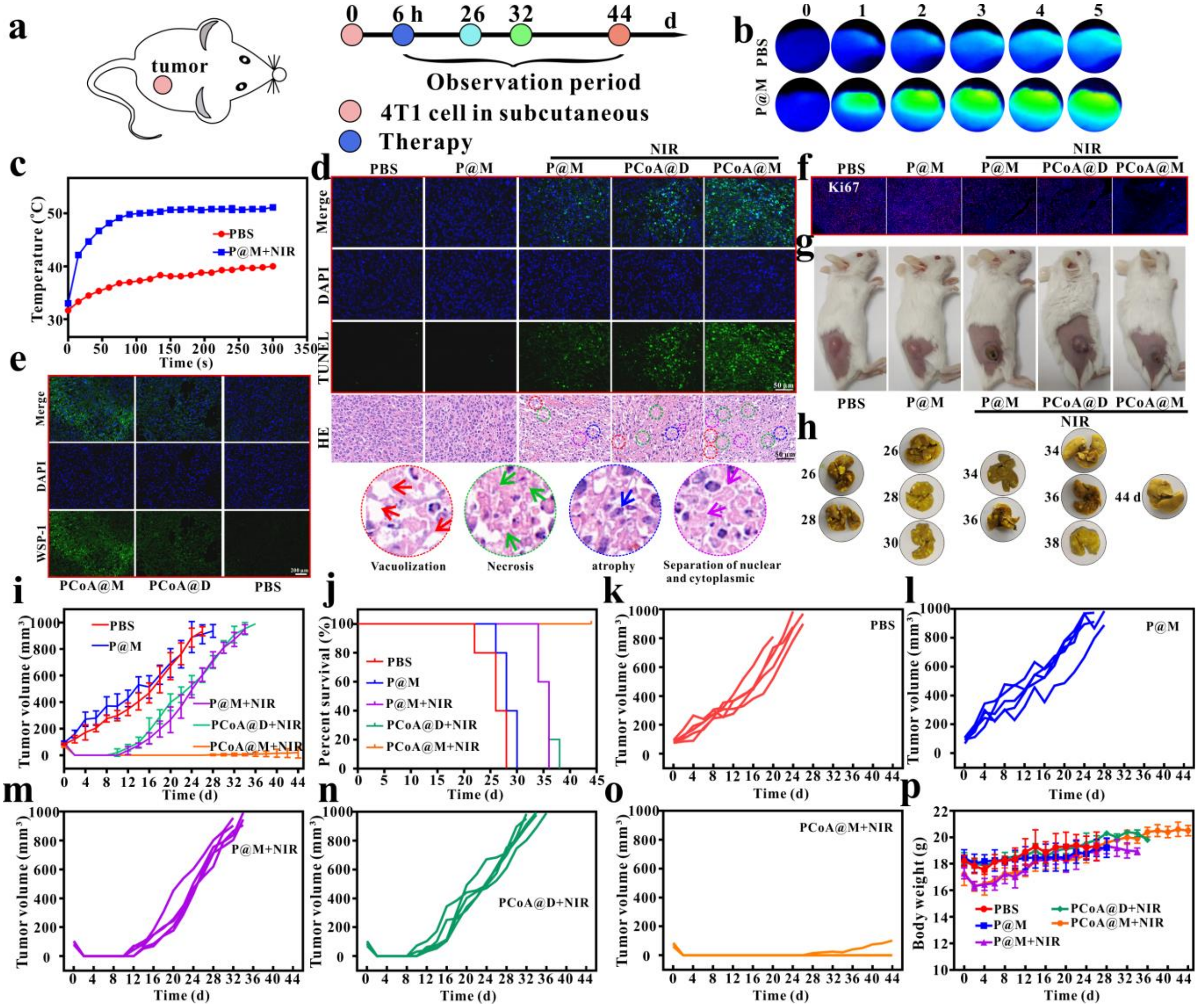

**Figure 7.** Treatment process of subcutaneous tumor (a); thermal image of tumor after injection of *in situ* 4T1 tumor-bearing mice with PBS, P@M and treated with NIR (b); temperature change curves with time (c); TUNEL and HE (d), WSP-1 (e), and Ki67 (f) immunofluorescence staining imaging of tumor site; bright-field imaging of mice after different treatments (g); the bright-field of the lung tissue of mice with subcutaneous 4T1 tumor-bearing mice after sacrifice, n=5 (h); mean tumor volume changes, n=5 (i), survival curve (j) after different treatments; changes of tumor volume in different parallel treatment groups (k-o); weight changes with time (p).

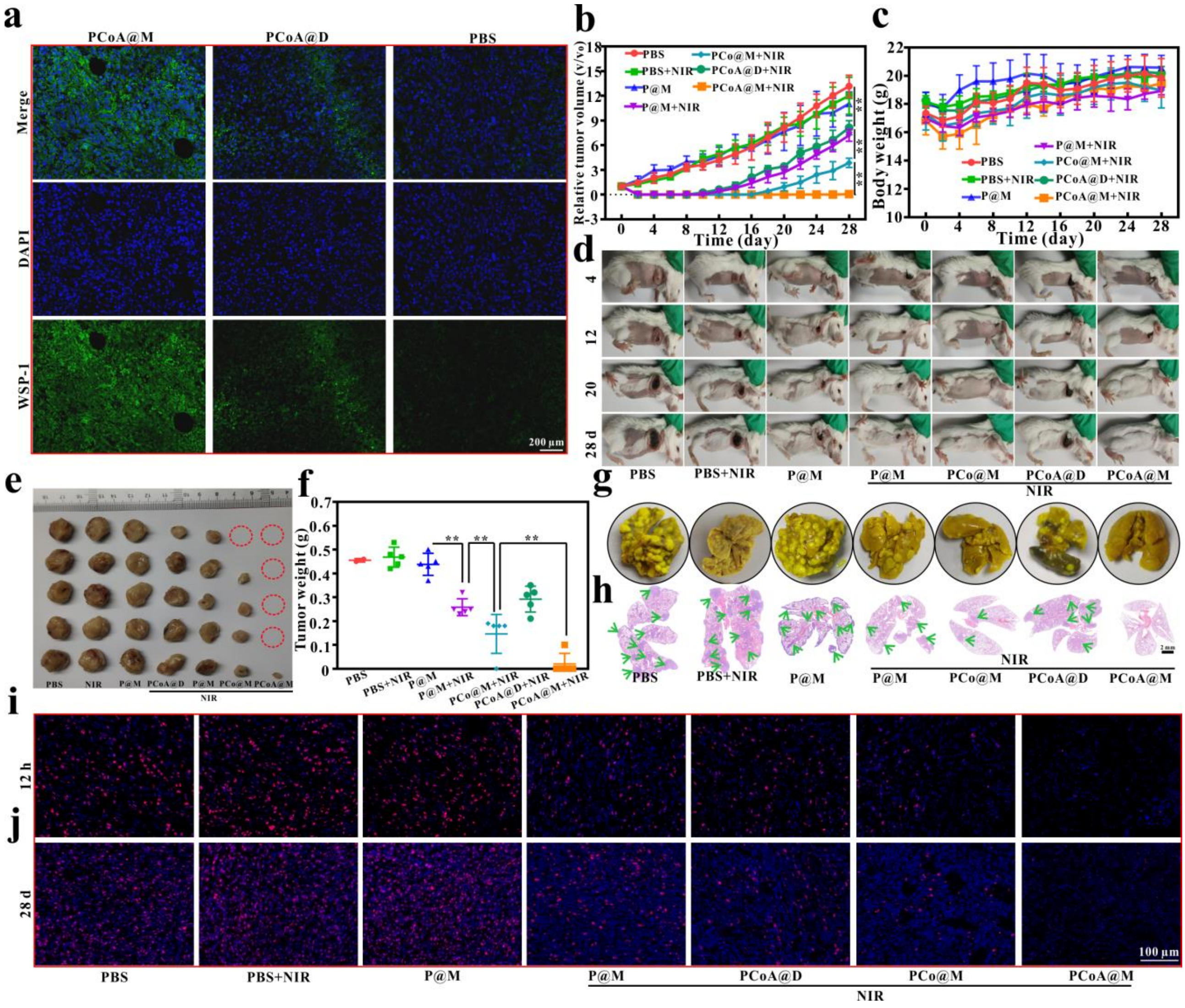

**Figure 9.** WSP-1 immunofluorescence staining imaging of tumor site after different treatments (a); relative tumor volume changes, n=5, **: p<0.01 (b), weight change (c), bright-field imaging of mice with *in situ* 4T1 tumor-bearing mice (d), the bright-field of tumors (e) and tumor quality analysis (f); the Bouins fixed lung tissue (g) of mice after sacrifice and the corresponding HE staining (h), Ki67 immunofluorescence staining imaging of tumor site under different time after different treatments (i and j).

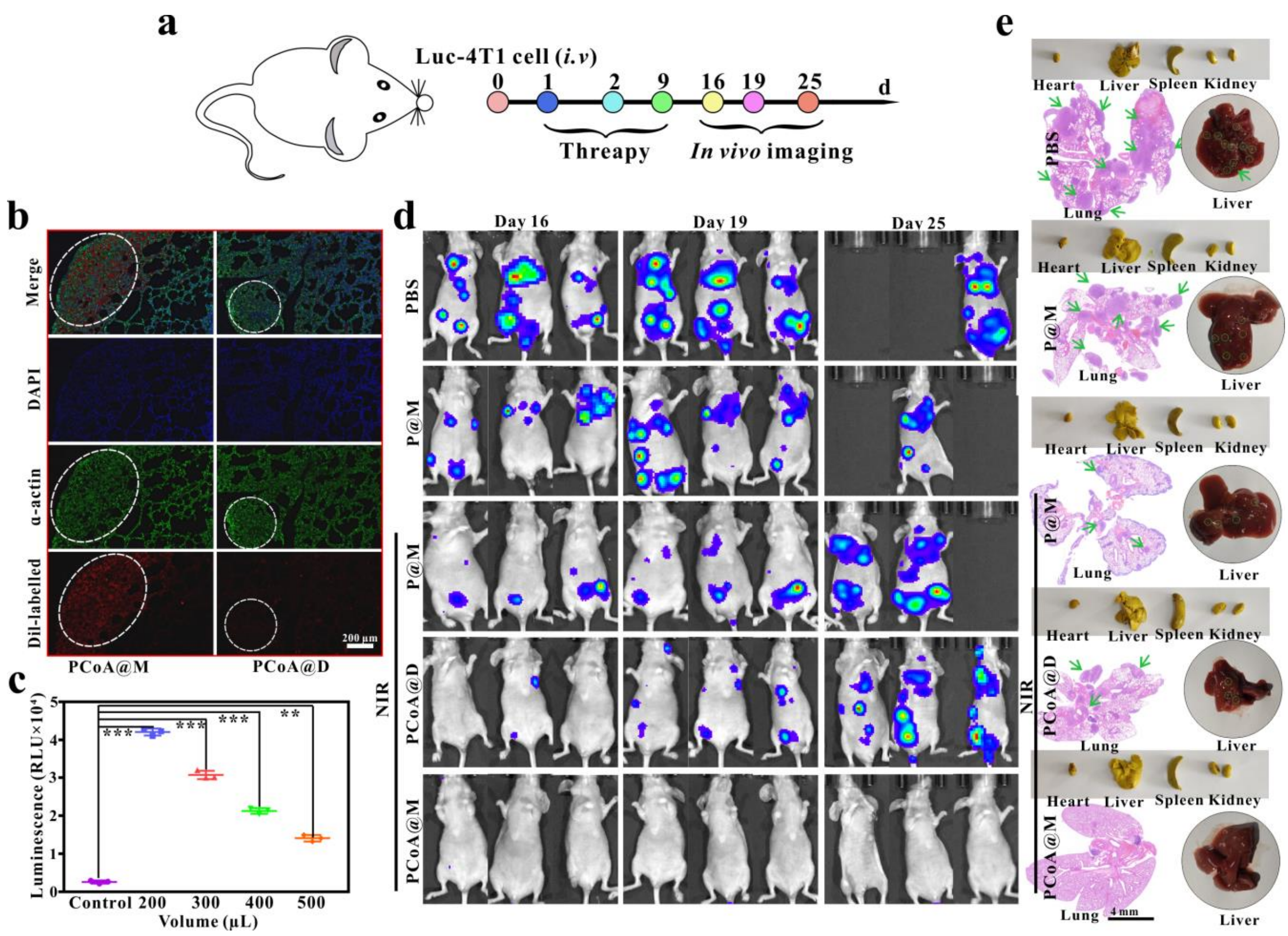


**Figure 10.** Treatment process of metastatic tumor model by injecting Luc-4T1 tumor cell into the tail vein (a); α-actin immunofluorescence imaging of lung tissues of metastatic tumor model mice after treatment with different Dil-labelled probes (b); luminescence intensity of luciferin reporter gene after Luc-4T1 cells incubation with different volumes of lysate, n=3, **: $p<0.01$ (c); bioluminescence imaging at different time points after injection of different probes into 4T1 tumor lung metastatic model mice (d); bright-field imaging of main organs fixed with Bouins (yellow), original liver and HE staining of corresponding lung tissues (e).

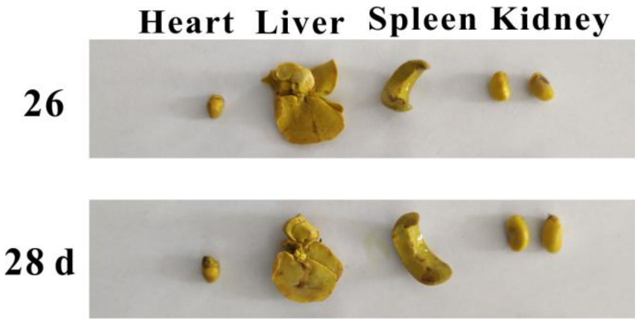


**Figure S35.** Bouins fixed organs map after PBS treatment of subcutaneous tumor.

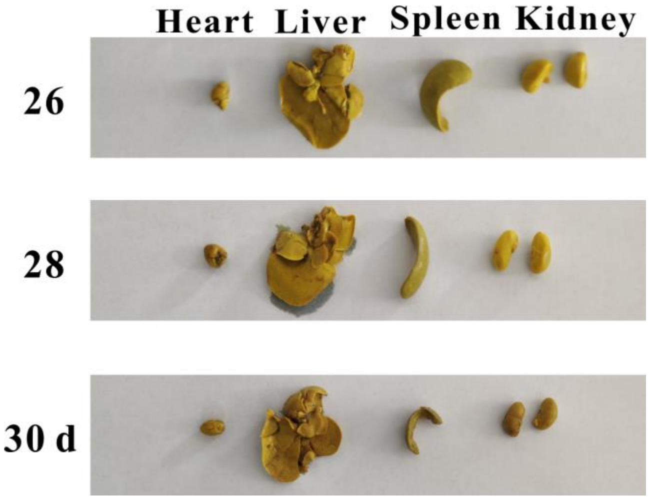


**Figure S36.** Bouins fixed organs map after P@M treatment of subcutaneous tumor.

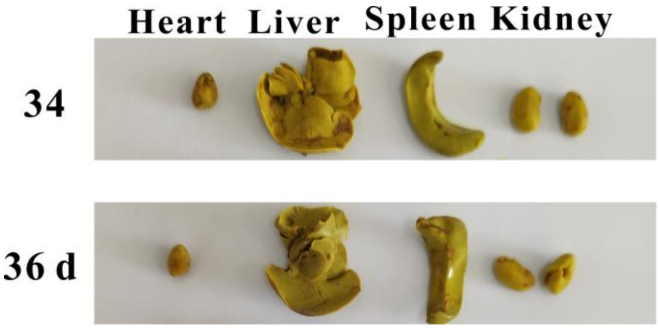


**Figure S37.** Bouins fixed organs map after P@M+NIR treatment of subcutaneous tumor.

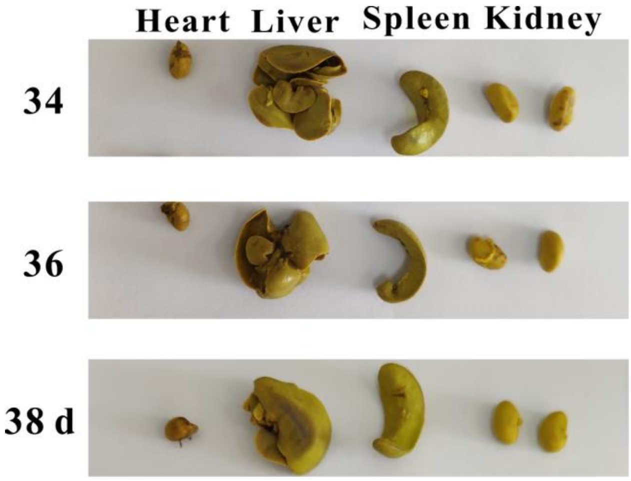


**Figure S38.** Bouins fixed organs map after PCoA@D+NIR treatment of subcutaneous tumor.

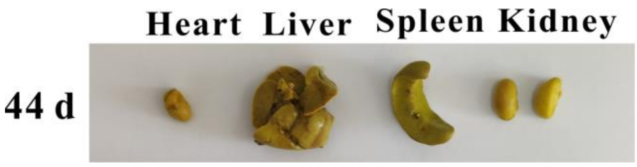


**Figure S39.** Bouins fixed organs map after PCoA@M+NIR treatment of subcutaneous tumor.

Several issues list as follow
1. Line 1, “attaention” should be replaced by “attention”;
Reply: Thanks for your suggestion. We are very sorry for such a low mistake, and we have made the changes and checked the full paper.

2. The author declare Co-MOF as MOF, but all through the paper there weren’t any evidence to support this, is there any TEM micrographs with higher magnification or BET data to show its frame hole and heterostructure?
Reply: Thanks for your suggestion. In order to identify the structure of nano-platform, we added some experiments. Firstly, for the cobalt-based MOF structure, the process of the cobalt-based MOF coating on the surface of PDA was investigated under different reaction conditions. The results exhibited that when the dosage of PDA was 0.5, 0.6, 0.7, 0.8, and 1 mg, the imidazole was 60 mg, and the rotation speed was 420 rpm, the amount of free hexagonal cobalt-based MOF was continuously decreased along with the increase of PDA dosage (Figure S1). When the dosage reached to 1 mg, there was almost no free cobalt-based MOF, indicating that MOF was mostly chelated on the surface of PDA, which proved the synthesis of heterostructure. Therefore, when the cobalt-based MOF was wrapped on the surface of PDA, we could not intuitively see the regular hexahedral structure of the MOF from the heterostructure, but during the wrapping process, it was obvious that with the increasing of PDA, the MOF of regular hexahedron decreased constantly, and the heterostructure in encapsulated state rose. This was precisely because the MOF was continuously chelated on the surface of the PDA, so we declared Co-MOF as MOF. This section was added in lines 14-20, page 6.

Next, the mesoporous and microporous structures of PCo were characterized by BET. The nitrogen adsorption-desorption isotherm of PCo (Figure 1k) showed a typical type IV isotherm with a hysteresis loop, and there existed obvious capillary condensation phenomenon in the lower relative partial pressure range ($P/P_0$=0.14~0.45) resulting from uniform mesopores; mesoporous BET total specific surface area and total pore volume were 1133.7655 $m^2/g$ and 0.720007 $cm^3/g$, respectively,

and the average pore diameter was 10.4916 nm. This section was added in lines 3-9, page 8.

Finally, the XPS results exhibited that there were characteristic peaks of N and Co in PCo (Figure 1j and Figure S6), while only characteristic peak of N in PDA (Figure S5), and the difference between PCo and PDA lied in the presence of cobalt-based MOF wrapping, while Co was a characteristic element in the cobalt-based MOF, so the XPS results further indicated that the cobalt-based MOF was successfully chelated on the PDA surface to form the heterostructure, which was also demonstrated by high-resolution elemental mapping detection of PCo (Figure S3 and S4), it was clear that PCo exhibited a well-proportioned distribution of N and Co elements. In summary, we declared Co-MOF as MOF. This section was added in lines 10-31, page 7, and lines 1-3, page 8.

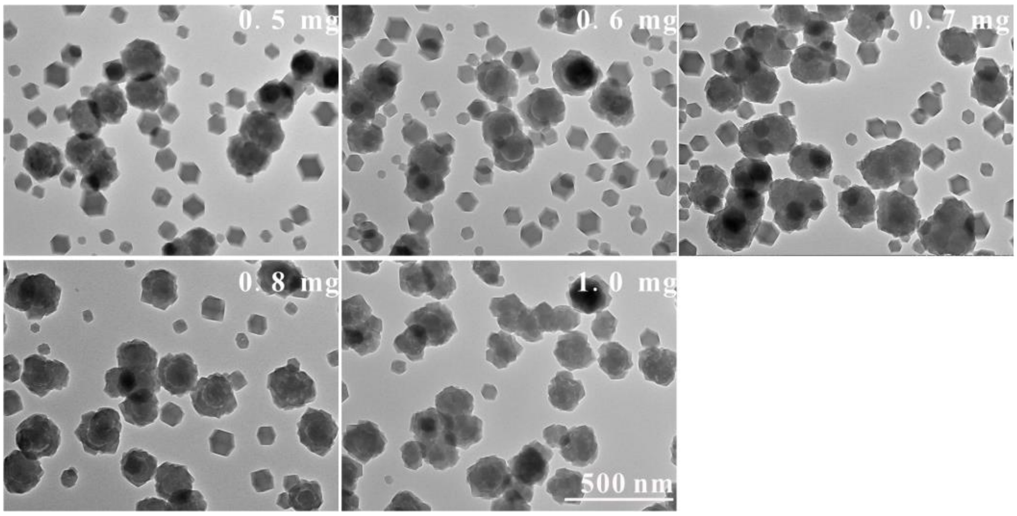


**Figure S1.** TEM of heterostructure formation under different PDA dosage.

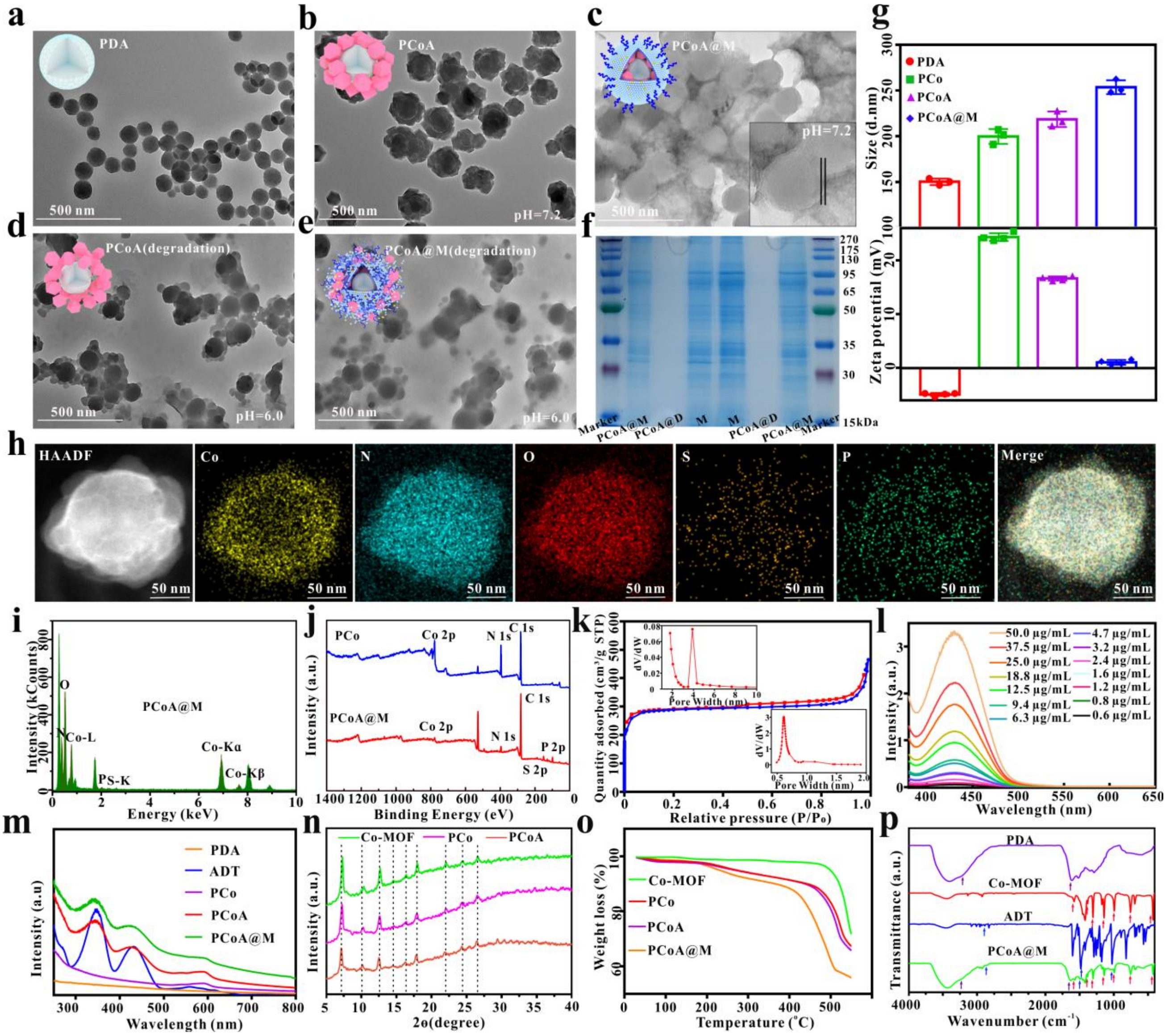

**Figure 1.** TEMs of PDA (a), PCoA (b) and PCoA@M (c) at pH=7.2; PCoA (d) and PCoA@M (e) at pH=6.0; SDS-PAGE protein analysis of macrophage membrane, PCoA@M and PCoA@D (f); the hydrated particle sizes, and zeta potentials of PDA, PCo, PCoA and PCoA@M (g); elemental mapping (h) and energy dispersive spectrometer (i) of PCoA@M; X-ray photoelectron spectroscopy of PCo and PCoA@M (j); nitrogen adsorption-desorption isothermal curve of PCo (k), inset: pore size distribution; absorption spectra of different concentrations of ADT (l); absorption spectra of PDA, PCo, PCoA and PCoA@M (m); X-ray powder diffractions of Co-MOF, PCo and PCoA (n); thermogravimetric analysis of Co-MOF, PCo, PCoA and PCoA@M (o); infrared spectra of PDA, Co-MOF, ADT and PCoA@M (p).

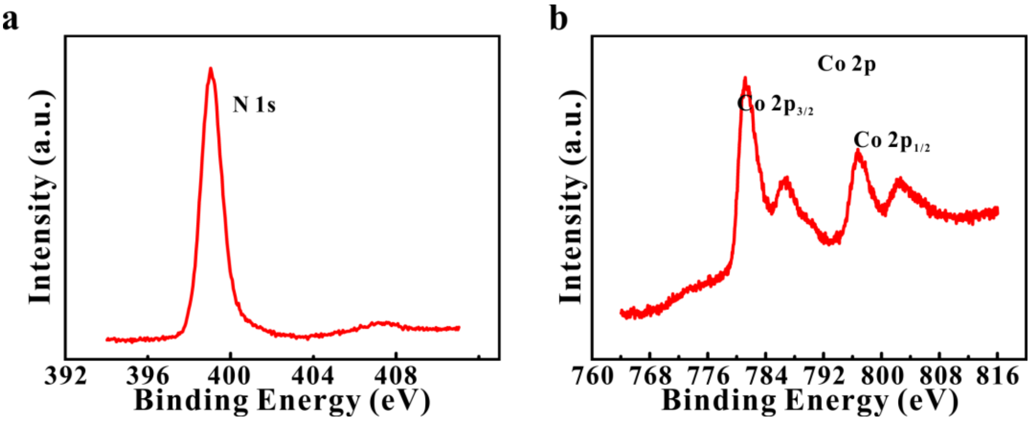


**Figure S6.** High-resolution XPS spectra of N (a) and Co (b) in PCo.

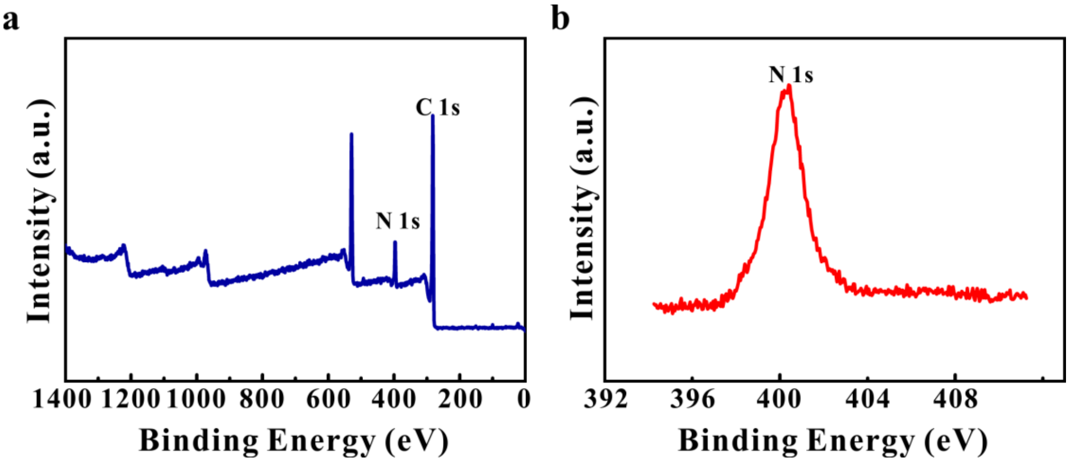


**Figure S5.** X-ray photoelectron spectroscopy of PDA (a) and high-resolution XPS spectra of N (b).

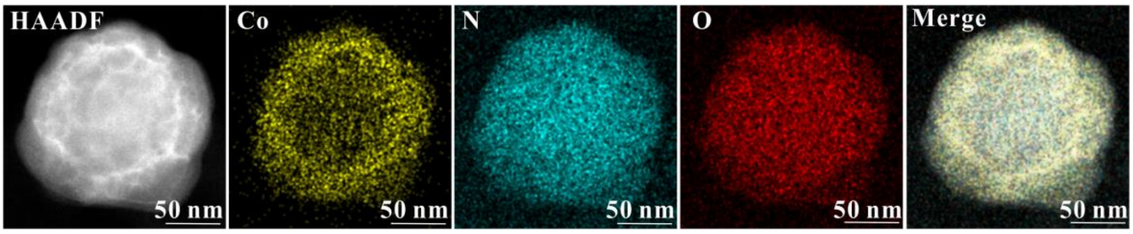


**Figure S3.** High angle annular dark field (HAADF) imaging and elemental mapping of PCo.

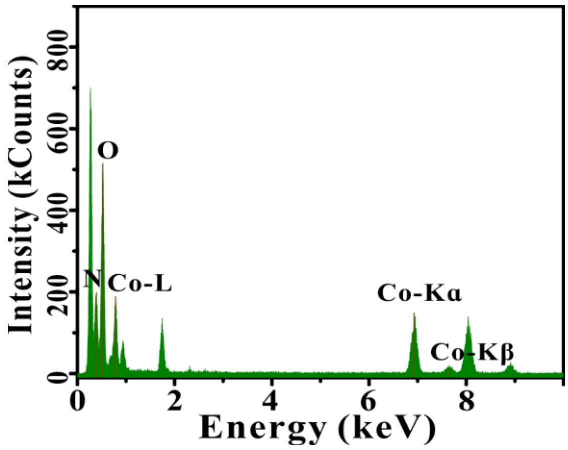


**Figure S4.** Energy dispersive spectroscopy analysis of PCo.

3. Line 383, “Compared with the P@M+NIR group, a large amount of red fluorescence appeared in the 4T1 cells of PCo@M+NIR group, maybe because the degradation of Co-MOF led to the large release of $Co^{2+}$, and down-regulated the expression of heat shock protein, which, in turn enhanced the PTT effect and prompted large numbers of cell apoptosis.” I am just curious about why the other content of Co-MOF can’t be the reason to down-regulate the expression of heat shock protein. In addition, the released $Co^{2+}$ played an important role in the whole process, then the release kinetic of $Co^{2+}$ should be added to support this statement.

Reply: Thanks for your suggestion. Here, the reason why cobalt ion was regarded to down-regulate the expression of heat shock protein rather than Co-MOF was that, in the cytotoxicity experiments (Figure 3d), the cell viability was not much different from the Co-MOF group when the cell was co-incubated with the same content of cobalt ion. The pH of DMEM high-glucose medium was previously detected to be about 6.0, in order to further verify our conjecture, we also adjusted the pH of the medium to be 7.2, and incubated Co-MOF probe with 4T1 cell for 6 h at pH 7.2 and 6.0 at the same time, respectively. Then, west blot detection was performed. The results exhibited that the expression of heat shock protein in the Co-MOF group incubated at pH 6.0 decreased significantly (Figure 3f and Figure S21), while the expression of heat shock protein in the Co-MOF group incubated at pH 7.2 was not significantly different from that in the PBS group. Therefore, we speculated that cobalt ion played a role in reducing the expression of heat shock protein.

To study the release of cobalt ion, the detection of Co element was carried out by atomic absorption spectrophotometer. PCoA@M (3 mg) was fixed with a dialysis bag and placed in a beaker containing 80 mL of PBS separately, and divided into (1) pH 5.5, (2) pH 6.0, and (3) pH 7.2 groups. At 10 min, 1, 2, 4, 8, 12, 24, 72, and 96 h, 200 μL of the solution was taken out for detection. The results showed that when the pH was 5.5, the release of cobalt ion increased with time (Figure 4a and S25), and reaching to 55.42% of the drug loading at 12 h; when the pH was 6.0, the release trend of cobalt ion was basically the same with pH 5.5, but the release amount was slightly smaller at the same time point, and the release amount was 48.15% at 12 h; while the pH was 7.2, the release amount of cobalt ion changed little along with time, and the release amount at 12 h was 2.63%, which was much lower than those of pH 5.5 and pH 6.0. The above results fully demonstrated that cobalt-based MOF possessed low pH responsiveness, and the lower the pH, the faster the degradation rate of cobalt-based MOF. This section was added in lines 11-22, page 18.

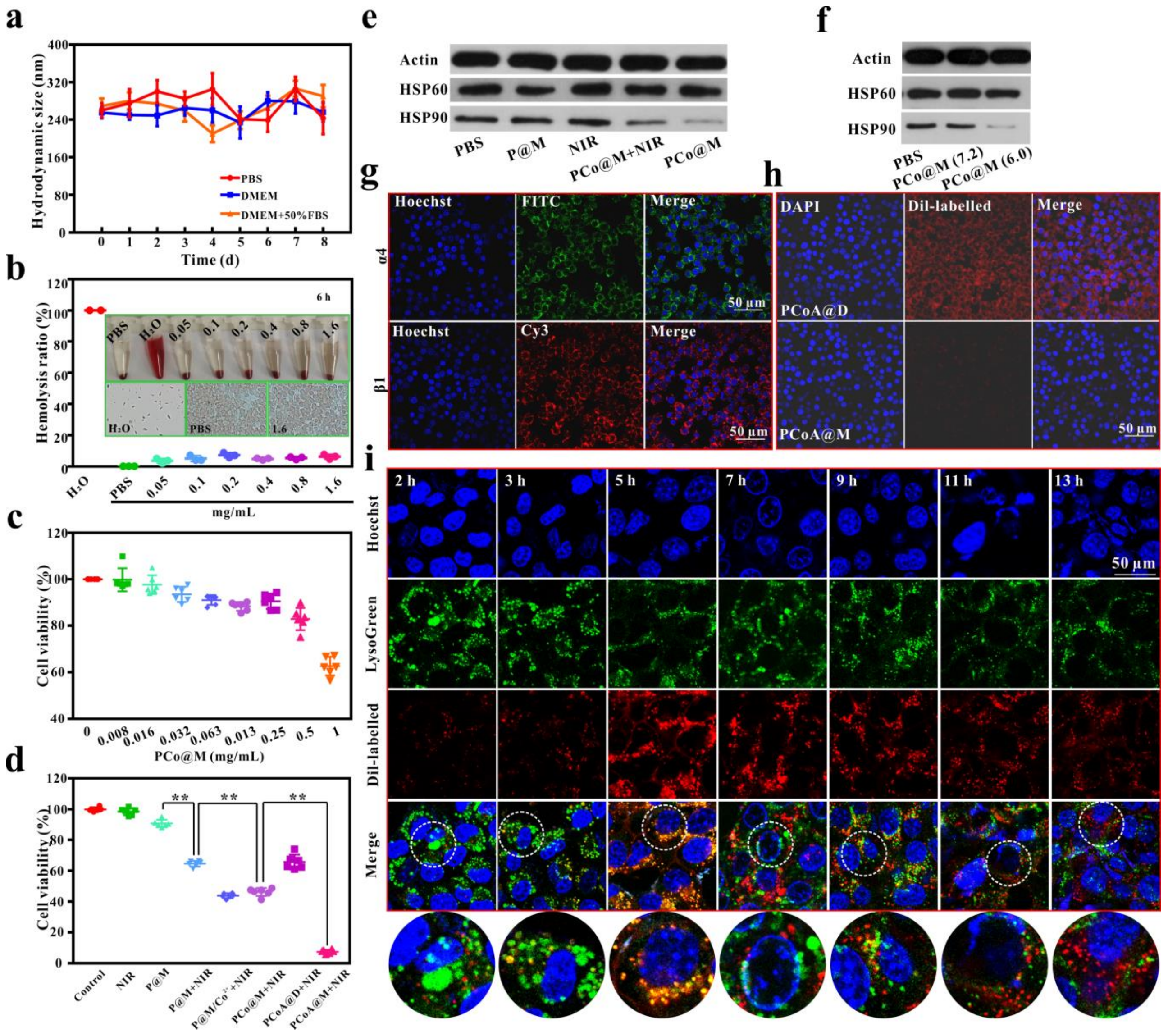


**Figure 3.** Hydrodynamic particle sizes of PCoA@M along with time in different media (a); the hemolysis rates of erythrocytes after incubation with different concentrations of probe, the inset was the bright-field image and the micrograph of the cell smear made by the precipitate after incubation (b); CCK-8 cytotoxicities of 4T1 cells incubated with different concentrations of PCo@M (c); survival rates of 4T1 cells incubated and treated with different probes, n=6, **: $p<0.01$ (d); west blot analysis of heat shock protein after different probe treatments of 4T1 cells (e) and treatments under different pH (f); immunofluorescence imaging for the detection of α4 and β1 antigens on the cell membrane of macrophages (g); confocal imaging of Dil-labeled PCoA@M and PCoA@D incubated with RAW264.7 for 5 h (h); Dil-labeled PCoA@M and 4T1 cells incubated for 2, 3, 5, 7, 9, 11, and 13 h and co-localized imaging (i).

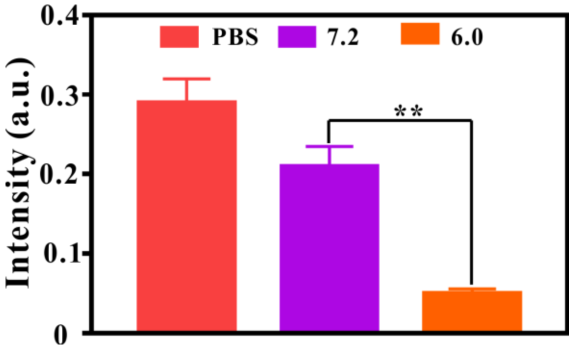


**Figure S21.** Gray-scale quantitative analysis of HSP90 in Figure 3f, n=3, **: p<0.01.

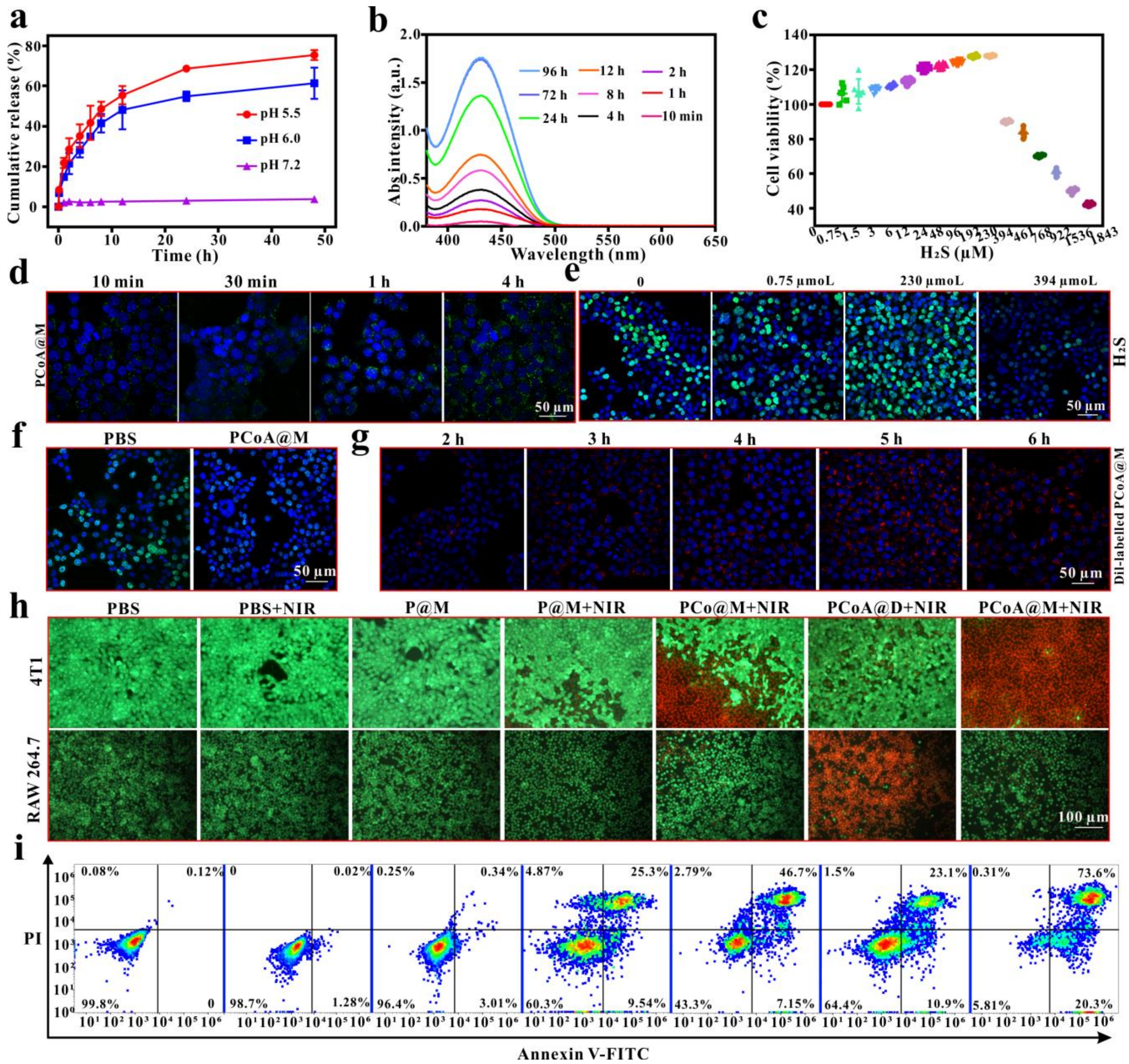


**Figure 4.** Cumulative release of cobalt ion at different times and pH (a); absorption spectra of ADT drugs released at different time points (b); CCK-8 cytotoxicities of 4T1 cells incubated with different concentrations of $H_2S$ (c); confocal imaging of 4T1 cells after incubation with PCoA@M+WSP-1 for different times (d); EDU proliferation of 4T1 cells co-incubated with different concentrations of $H_2S$ (e); EDU proliferation of 4T1 cells co-incubated with PBS and PCoA@M (f); confocal imaging of 4T1 cells after incubation with Dil-labeled PCoA@M for different times (g); fluorescence imaging of calcein/PI staining 4T1 and RAW 264.7 cells incubated with different probes under different conditions (h); flow cytometry analysis of 4T1 cells' apoptosis under above different conditions (i).

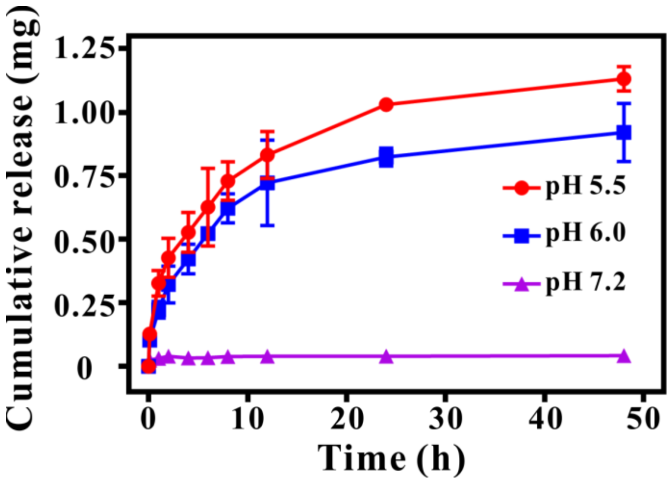


**Figure S25.** Release of cobalt ions at different times and pH.

4. The images of the tumors should be put in the figure to support figure7's study.

Reply: Thanks for your suggestion. The brightfield images of tumors treated with different probes were added to the insets of Figure 8f (Figure 7C in previous version).

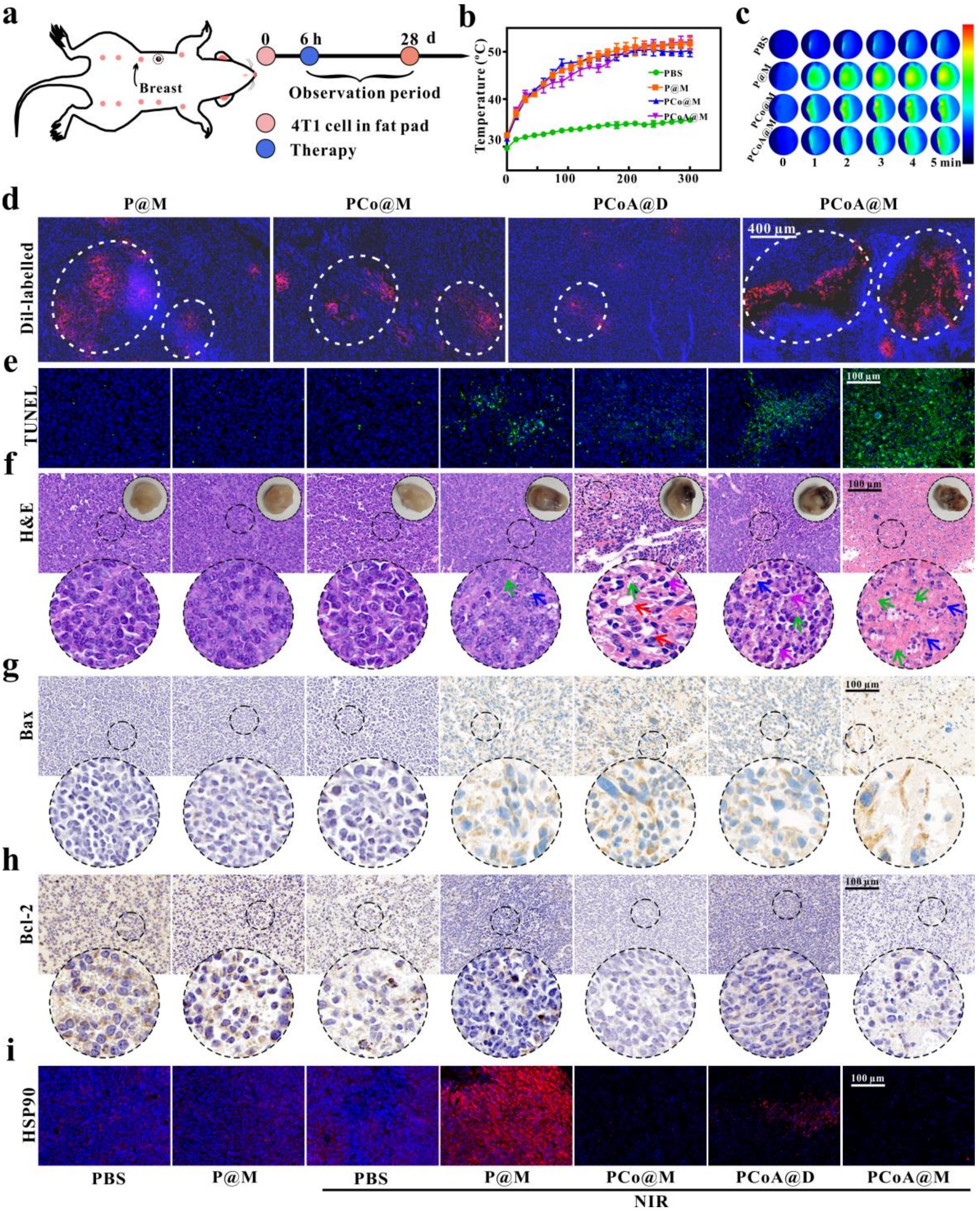


**Figure 8.** Treatment process of *in situ* tumor (a); temperature change curves with time (b) and its thermal image (c) of tumor after injection of *in situ* 4T1 tumor-bearing mice with PBS, P@M, PCo@M and PCoA@M and treated with NIR; fluorescence imaging of 4T1 tumor sections after injection of different Dil-labelled probes to mice (d); TUNEL (e), HE (f), Bax (g), Bcl-2 (h) and HSP90 (i) immunofluorescence staining imaging of tumor site; red arrow in f: vacuolization of cell, blue arrow: cell necrosis; green arrow: cell atrophy, purple arrow: separation of nuclear and cytoplasmic.

5. The images of fluorescence were dark, please make it brighter to ensure clear pictures.

Reply: Thanks for your suggestion. In order to verify the fluorescence images in the full paper, the fluorescence intensities in Figures 8, Figures 9, and Figures 10 were made corresponding overall adjustment, which made them brighter and clearer.

New Figure 8 was shown in Reply to Question 4 (last question).

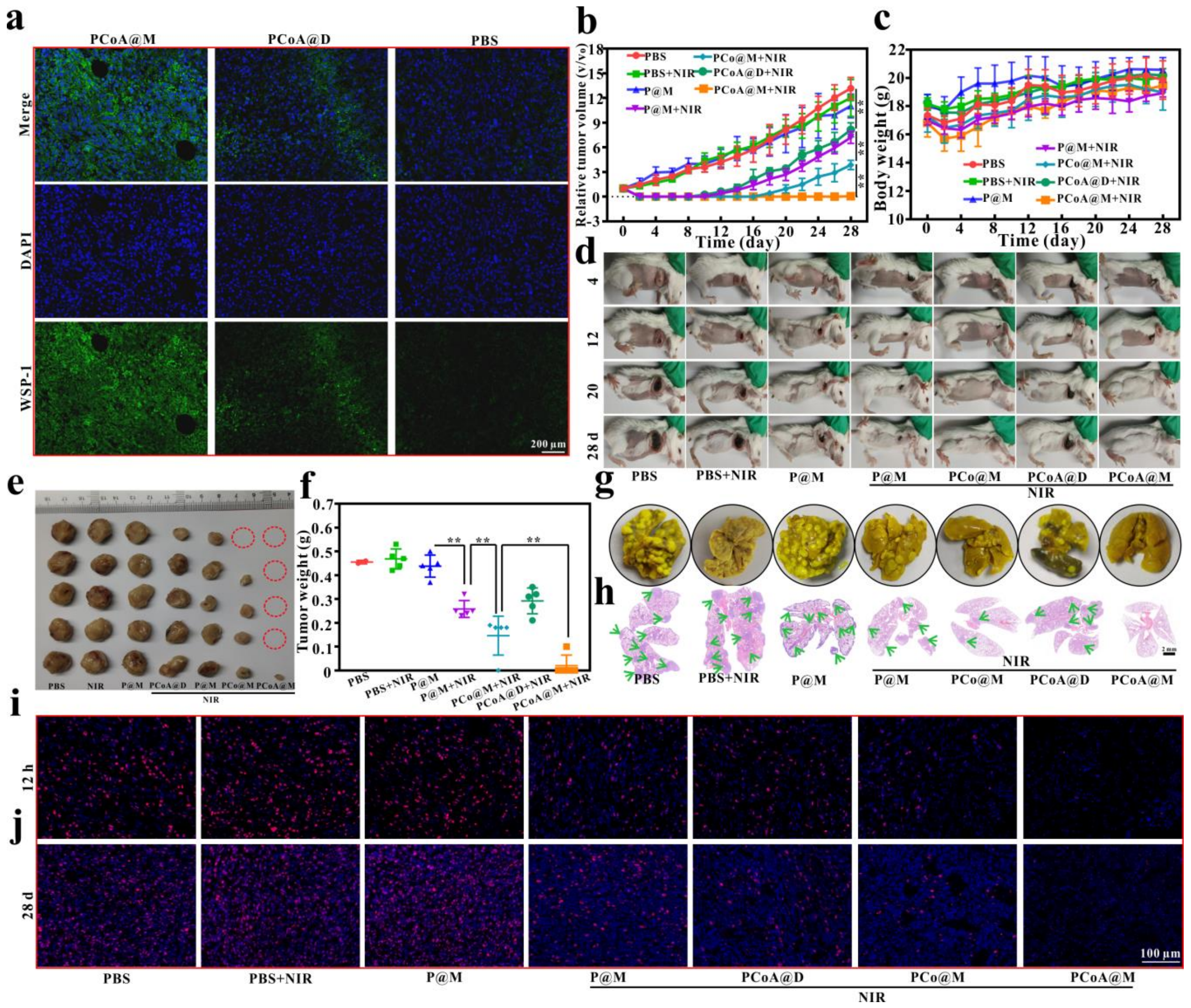

**Figure 9.** WSP-1 immunofluorescence staining imaging of tumor site after different treatments (a); relative tumor volume changes, n=5, **: p<0.01 (b), weight change (c), bright-field imaging of mice with *in situ* 4T1 tumor-bearing mice (d), the bright-field of tumors (e) and tumor quality analysis (f); the Bouins fixed lung tissue (g) of mice after sacrifice and the corresponding HE staining (h), Ki67 immunofluorescence staining imaging of tumor site under different time after different treatments (i and j).

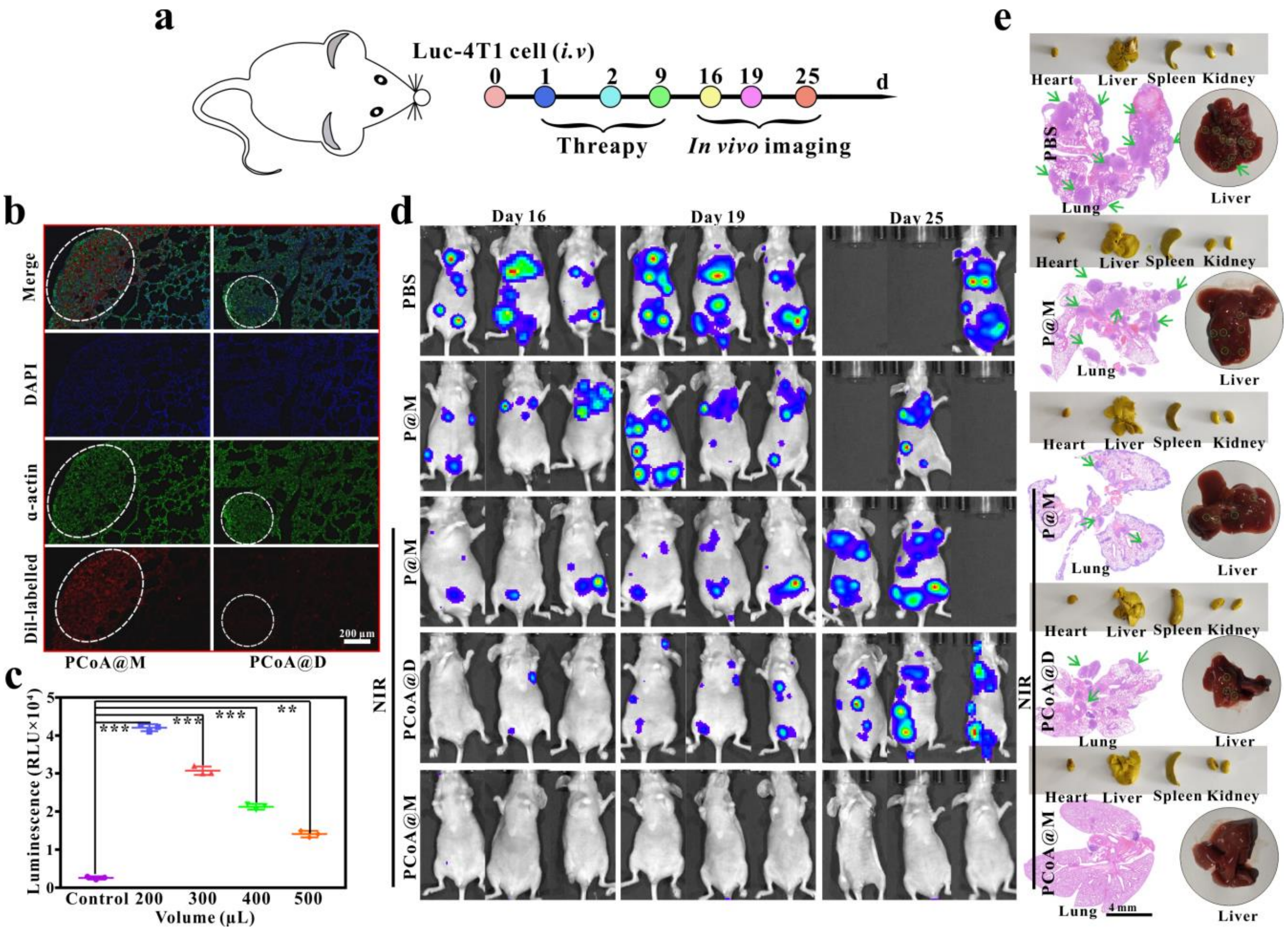


**Figure 10.** Treatment process of metastatic tumor model by injecting Luc-4T1 tumor cell into the tail vein (a); α-actin immunofluorescence imaging of lung tissues of metastatic tumor model mice after treatment with different Dil-labelled probes (b); luminescence intensity of luciferin reporter gene after Luc-4T1 cells incubation with different volumes of lysate, n=3, **: $p<0.01$ (c); bioluminescence imaging at different time points after injection of different probes into 4T1 tumor lung metastatic model mice (d); bright-field imaging of main organs fixed with Bouins (yellow), original liver and HE staining of corresponding lung tissues (e).

6. Line 509, the authors stated that "In comparison, P@M+NIR and PCo@M+NIR groups displayed different degrees of vacuolization, cell necrosis, atrophy, and separation of nuclear and cytoplasmic." However, there is no result to illustrate or support this finding.

Reply: Thanks for your suggestion. Vacuolization of cytoplasmic generally refers to the condensation of cytoplasm in cells due to the loss of water, resulting in vacuoles. Cell necrosis is a passive behavior, which is often found in large tissues or groups of cells, and the cell membrane will also be damaged. The necrotic cell may swell and disintegrate the endoplasmic reticulum, and the nuclear and cytoplasmic separation of cells often occurs during cell necrosis. Cell atrophy usually refers to a phenomenon, in which the nuclei are close to each other resulted from the decrease of the functional activity of the cells and the insufficient supply of blood and nutrients. Due to thermal ablation, the cells will lose lots of water, resulting in vacuolization of the cytoplasm. When the heat was accumulated to a certain level, the cells were prone to necrosis, and the inhibition of heat shock protein would further improve the probe's thermal ablation effect. And due to the pH-responsive release of the $H_2S$ prodrugs at the lesion site, the $H_2S$ would be further produced, and the accumulation of a large amount of $H_2S$ gas at the tumor site would result in a high concentration of $H_2S$, which could inhibit cell growth, that was, cell apoptosis and necrosis were produced; in addition, the release of the drug further inhibited the production of ATP, then resulting in gas-starvation treatment. In order to illustrate the phenomena clearly, we specially marked them on the figures, red represented vacuolization of cell; blue represented cell necrosis; green represented cell atrophy, and purple represented separation of nuclear and cytoplasmic (Figure 7d and 8f).

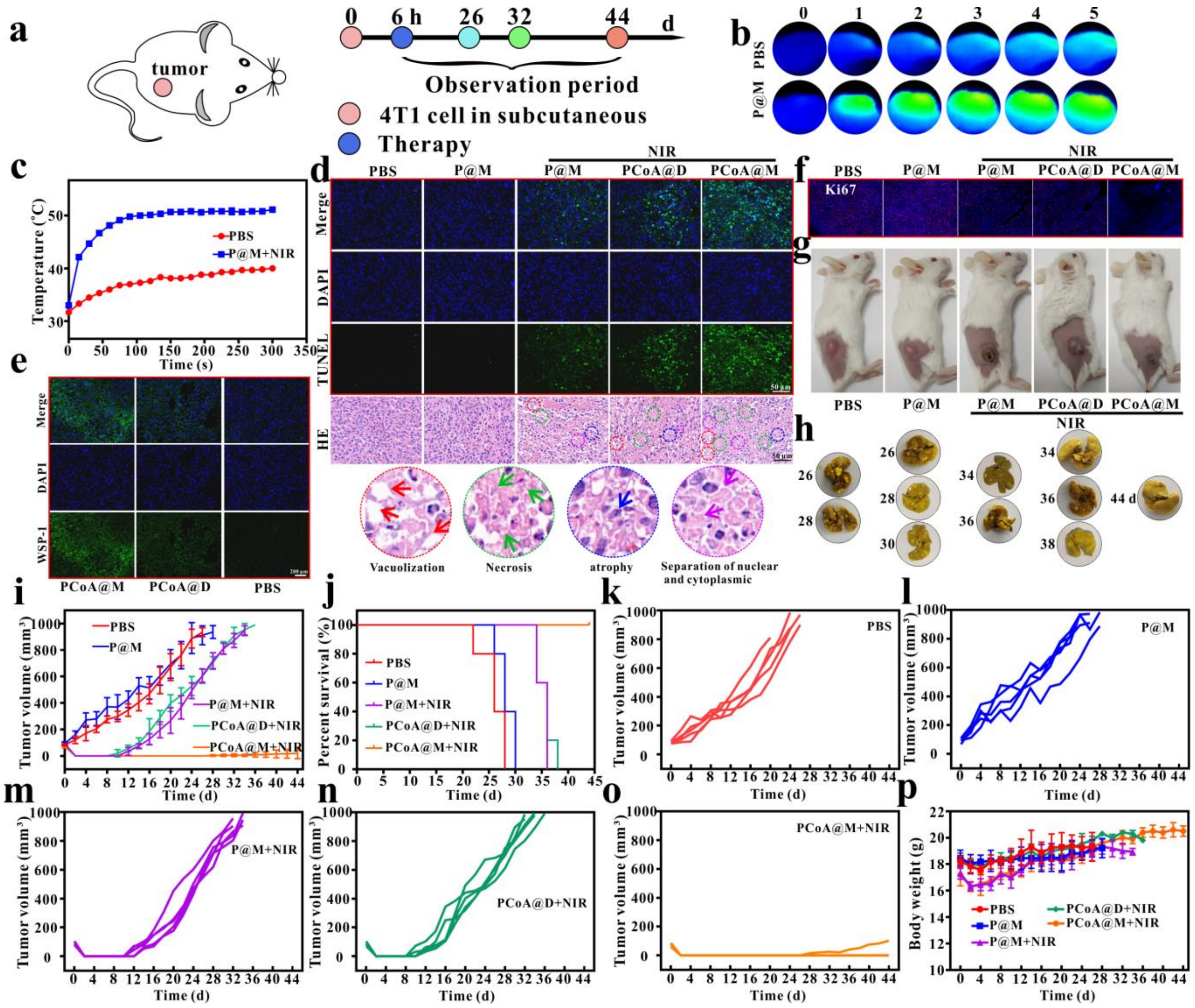

**Figure 7.** Treatment process of subcutaneous tumor (a); thermal image of tumor after injection of *in situ* 4T1 tumor-bearing mice with PBS, P@M and treated with NIR (b); temperature change curves with time (c); TUNEL and HE (d), WSP-1 (e), and Ki67 (f) immunofluorescence staining imaging of tumor site; bright-field imaging of mice after different treatments (g); the bright-field of the lung tissue of mice with subcutaneous 4T1 tumor-bearing mice after sacrifice, n=5 (h); mean tumor volume changes, n=5 (i), survival curve (j) after different treatments; changes of tumor volume in different parallel treatment groups (k-o); weight changes with time (p).

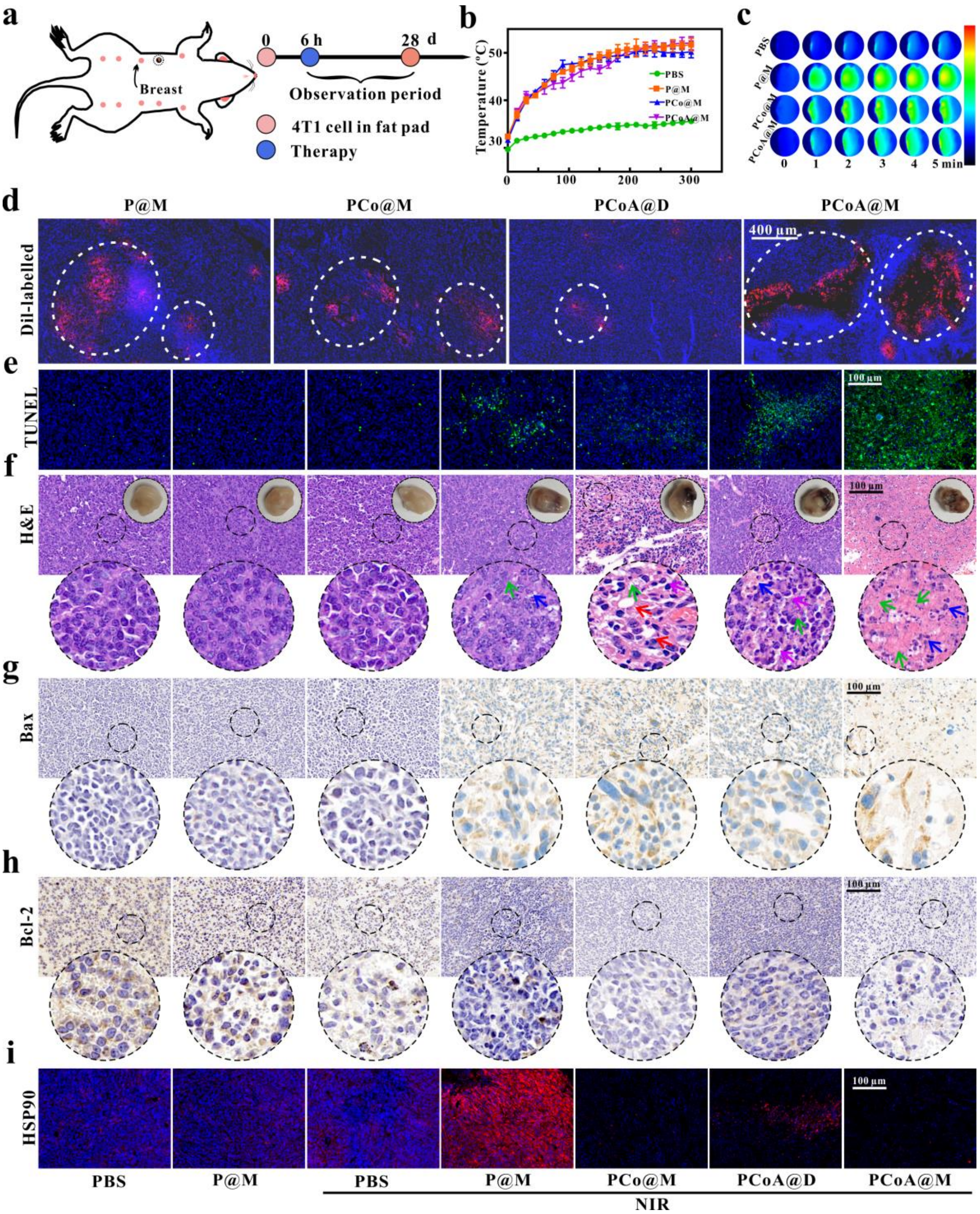


**Figure 8.** Treatment process of *in situ* tumor (a); temperature change curves with time (b) and its thermal image (c) of tumor after injection of *in situ* 4T1 tumor-bearing mice with PBS, P@M, PCo@M and PCoA@M and treated with NIR; fluorescence imaging of 4T1 tumor sections after injection of different Dil-labelled probes to mice (d); TUNEL (e), HE (f), Bax (g), Bcl-2 (h) and HSP90 (i) immunofluorescence staining imaging of tumor site; red arrow in F: vacuolization of cell, blue arrow: cell necrosis; green arrow: cell atrophy, purple arrow: separation of nuclear and cytoplasmic.

7. It's difficult to find clear pictures of the positive biomarkers in figure7D and figure7E. So it's necessary to get some more typical views under a high-scale microscope.

Reply: Thanks for your suggestion. In Figure 8g and Figure 8h (Figure 7D and Figure 7E in previous version), typical areas of positive signal were selected and accordingly zoomed.

New Figure 8 was shown in Reply to Question 6 (last question).

8. Line 610, the authors stated that "Because normal lung tissue contains a large amount of dense myofilament actin, α-SMA fluorescent staining can well reflect this situation." Several evidences suggested that tumor tissues can get a great α-SMA fluorescent staining. Please consider whether this indicator is a reasonable choice.

Reply: Thanks for your suggestion. After careful consideration, we thought you gave us a good advice to improve this work. Based on the fibrosis of lung tissue, α-actin immunofluorescence staining of lung tissue was carried out. Through the localization of its cytoskeleton, the metastatic tumor area in lung tissue could be more clearly displayed. The results showed that in the metastatic area, the cytoskeleton was denser (Figure 10b), and the normal tissue sites showed fibrosis. With the tail vein injection of Dil-labeled PCoA@M and PCoA@D probes, it was obvious that in the lungs, the metastatic area of the tumor appeared significant red fluorescence signal in the PCoA@M group, but hardly presented in the PCoA@D group, which further demonstrated the targeting effect of our probe in lung metastatic tumor.

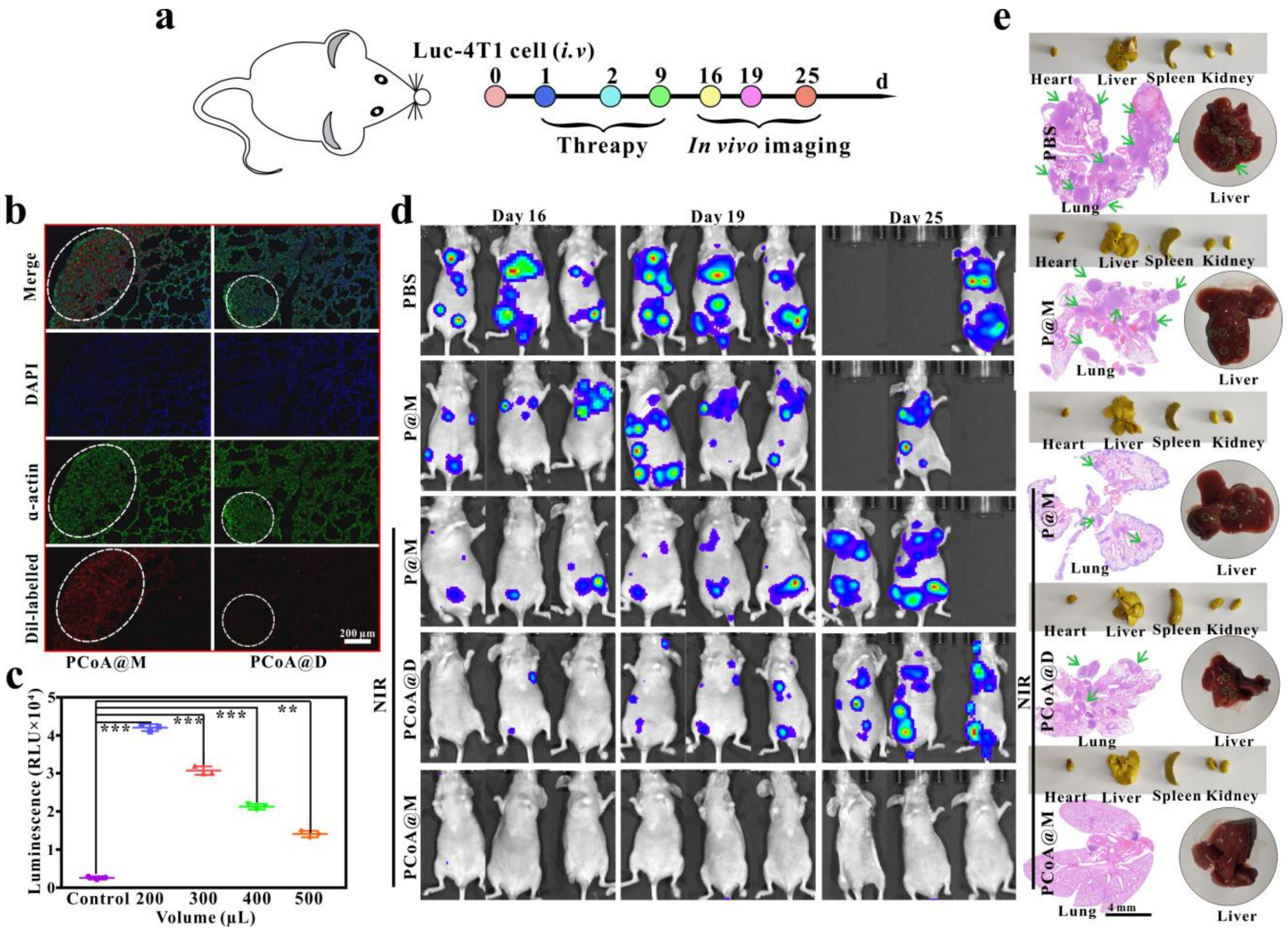


**Figure 10.** Treatment process of metastatic tumor model by injecting Luc-4T1 tumor cell into the tail vein (a); α-actin immunofluorescence imaging of lung tissues of metastatic tumor model mice after treatment with different Dil-labelled probes (b); luminescence intensity of luciferin reporter gene after Luc-4T1 cells incubation with different volumes of lysate, n=3, **: $p$<0.01 (c); bioluminescence imaging at different time points after injection of different probes into 4T1 tumor lung metastatic model mice (d); bright-field imaging of main organs fixed with Bouins (yellow), original liver and HE staining of corresponding lung tissues (e).

**Reviewer #3** (Remarks to the Author): Expert in nanoparticles

The authors of this paper designed a macrophage membrane nanoplatform grown on PDA as the core and co MOF as the shell layer to load H2S releasing precursor trithioanisine (ADT) into the pores of the MOF and the interstices of the heterostructure, and finally modified the macrophage membrane with a series of modifications. However, conceptual innovation is rather less. This is just good-considered combination of pre-existing systems and idea. It includes possible aspect into one system and nothing more. This work well perform on (i) materials characterization and (ii) biomedical effects. However, scientific resonation between these data, innovative concept, and surprising finding beyond expectable effects upon the combination are not well included. Therefore, insights in nanosciece and nanotechnology are not well included although it gives comprehensive results on materials for biomedical applications. There are no serious comparisons over huge numbers of related research works in the past literatures. I do not think that Nature Communications is suitable medium for this work.

Reply: Thanks for your suggestion. Firstly, the synthesized heterostructure was slightly different from the conventional core-shell structure. In 2020, a 20 nm shell of zinc-based MOF was wrapped on the surface of PDA by Feng et al (Chemical Science, 2020, 11 (6): 1649-1656). This core-shell structure consisted of a single zinc-based MOF and PDA (Figure R1), but more $Co^{2+}$ was needed according to our original design, whereas cobalt-based MOF bound to the surface of a single PDA particle in this core-shell structure were limited (Figure R1), so combining with more cobalt-based MOF on the PDA surface was expected. Subsequently for this purpose, we attempted to synthesize a heterostructure binding with more cobalt-based MOF on the PDA surface by changing the reaction conditions. Excitingly, this idea was feasible, and the results showed (Figure S1) that by changing the reaction ratio of PDA, cobalt nitrate, and dimethylimidazole, this goal of binding more MOF on the surface of PDA was indeed achieved. Here, the reason why PDA particle was selected was that the phenolic hydroxyl group had a strong chelating effect on metal ion, which was conducive to combining a large amount of cobalt ions on the surface of PDA. By adjusting the raw material ratio, as many intact cobalt-based MOF as possible were formed on the PDA surface.

Secondly, in the traditional process of photothermal treatment, the generation of heat resistance will greatly reduce the efficiency of heat treatment, and the fundamental reason for heat resistance is that the production of heat shock proteins promote the enhancement of body tolerance to heat. Now, most heat shock protein inhibitors in clinic are mainly concentrated on small molecules, and nanomaterial-based inhibitor has been rarely reported. Some metal ions were also found to possess the effect of binding heat shock proteins, and in this manuscript, cobalt ion was found to possess the ability of binding heat shock protein and inhibiting its expression by us for the first time, and

cobalt-based MOF became an ideal choice due to the acid degradation ability. According to literature research, cobalt-based MOF had not been used as inhibitor for heat shock proteins.

$H_2S$, as a signal molecule, is widely involved in the regulation process of the digestive, nervous, and respiratory system, and is also closely related to the occurrence and development of tumors. As a clinical drug, ADT, a prodrug of $H_2S$, has also been widely used in the treatment of liver and gallbladder diseases, which laid a good clinical foundation for the design of the nanocarriers. NADH and FAD are two coenzymes with autofluorescence properties in the mitochondrial respiratory chain. The content of NADH and FAD in tissues can represent the reduction and oxidation state of cells, respectively. In the process of glucose metabolism, the ATP directly generated by glucose metabolism is very small, while the metabolically generated NADH can react with oxidative phosphoric acid through electron transfer to generate a large amount of ATP, so NADH can be regarded as an endogenous fluorescent metabolic signal to characterize the redox energy state of mitochondrial metabolism. Now, the application of endogenous fluorescent metabolic signals to characterize the state of energy metabolism is mainly concentrated at the cellular level. The main challenge in extending it to the tissue level is that whether the optical characterization of energy metabolism can truly reflect the complex physiological and pathological responses of biological tissues. Fortunately, this problem was solved with a self-built NADH/FAD cryogenic fluorescence imaging system by us. ADT drug can react with NADH, which can further inhibit the synthesis pathway of ATP, resulting in insufficient energy supply, and then starvation therapy in the lesions. Therefore, the various components in the nano-platform were interdependent, that was, acid-responsive cobalt-based MOF could release cobalt ions to significantly enhance the photothermal therapeutic effect of PDA, an organic photothermal material. Furthermore, the ADT drugs loaded in the cobalt-based MOF were released to the greatest extent due to the degradation effect, resulting in high concentration enrichment of $H_2S$, inhibiting ATP synthesis pathway in the energy metabolism, and enhancing the amplification effect of thermal therapy. Next, for the evaluation of the therapeutic effect of the designed nanoplatform, it was compared with the commonly used clinical drugs. Now, the clinical drug treatment of tumor is based on NCCN (National Comprehensive Cancer Network) or CSCO (Chinese Society of Clinical Oncology) tumor treatment guidelines. First, the most suitable first-line drugs are selected, if poor tumor inhibition effect and drug resistance appear, second-line drugs and later-line drugs are used in combination. For

the treatment of breast cancer, the commonly used first-line treatment is the combination of CDK4/6 inhibitors such as palbociclib with endocrine drugs. Clinical studies showed that the progression-free survival time of patients in the palbociclib combination group was 27.6 months. For the second- and later-line treatment, patients who progressed after first-line endocrine therapy and have not received CDK4/6 inhibitor therapy can choose CDK4/6 inhibitors such as palbociclib combined with fulvestrant or aromatase inhibitor. Here, the progression-free survival time of patients treated with palbociclib and fulvestrant was 46.7 months. According to the age of human and rat (a 12-month-old rat is equal to a thirty-year-old human), the first-line combination therapy can prolong the survival time of rats by 27.6 d, and the second-line and later-line treatment can prolong the survival time of rats by 46.7 d.

In this paper, tumor model and lung metastasis model were established by subcutaneous (Figure 7), orthotopic (Figure 9), and tail vein (Figure 10) injection, respectively. The subcutaneous injection method was to form a solid tumor area by subcutaneously injecting a certain concentration of tumor cells into the back of the mouse. In order to strictly protect animal welfare and comply with animal ethics standards, when the tumor volume reached to 1000 $mm^3$ or the mice died naturally, this time was defined as the death time of the mouse. When the tumor volume reached to about 100 $mm^3$, different probes (PBS, P@M, P@M+NIR, PCoA@D+NIR, and PCoA@M+NIR) were injected. According to statistical data, the average death time of mice in the PBS group was 26 d (Figure 7k), while only one mouse in the PCoA@M+NIR group still developed tumor recurrence at 44 d (Figure 7o). Based on the growth rate of tumor recurrence, we calculated that it would take at least 26 d for the tumor recurrence mouse to reach a volume of 1000 $mm^3$, which would be considered to die, that was, the survival time of mice treated with PCoA@M+NIR was extended by at least 44 d (for the recurrence one), which was superior to the first-line combination drug of clinical breast cancer treatment, so we thought this probe was promise to be a supplement for the first-line drugs. By dissecting the organs of the mice at the sacrifice time, it was obvious that there was few metastatic nodule in the lungs of PBS and P@M groups (Figure 7h), which could not form effective lung metastasis of breast cancer, and no obvious metastatic nodule appeared in organs such as the heart, liver, spleen, and kidney (Figure S35-39). It also showed that although the subcutaneous injection method was convenient and simple to operate, the formation rate of tumor *in situ* was low, so there were many restrictions on modeling. Therefore, orthotopic injection of tumor cells into the second

pair of mammary glands in mice was performed. When the tumor volume reached to 100 $mm^3$, different probes (PBS, P@M, PBS+NIR, P@M+NIR, PCo@M+NIR, PCoA@D+NIR, and PCoA@M+NIR) were injected. After 28 d of treatment, lung tissue was dissected out. It was obvious that significant increase in number of metastatic lung nodule occurred in PBS, P@M, and PBS+NIR groups (Figure 9g and h), which also indicated that the orthotopic injection method was easier to establish the lung metastatic tumor model than the subcutaneous injection method, and comparing with the other treatment groups, the PCoA@M+NIR group had significantly fewer lung metastatic nodule, implying that the PCoA@M probe produced a strong effect of metastatic inhibition on the orthotopic tumor. As we know, the key of tumor metastasis is that tumor cells in tumor tissue migrate into the blood, reaching to all the body through blood circulation, and form new cell implantation sites. The reason why breast cancer cells form more lung metastasis is due to short-term proximal metastases, that is, the lung is relatively close to the breast. So, to establish a lung metastasis model more quickly, Luc-4T1 cells were injected into mice through the tail vein. After 1, 2, and 9 d of treatment (Figure 10a), bioluminescence images on 16, 19, and 25 d of mice were performed, and for PCoA@M+NIR group, it was clear that no obvious metastatic tumor cell signal appeared in the lungs and other organs (Figure 10d), while other groups had a certain degree of metastatic tumor cell signal, which fully exhibited that the PCoA@M probe possessed strong inhibitory and killing effects on metastatic cells. The mice were dissected after 25 d, and a large number of aggregated tumor cell populations were found in lungs of PBS group by HE staining (Figure 10e), which was significantly different from pulmonary fibrosis, and a large number of metastatic plaques were also present in the liver, while it was relatively few in the PCoA@M+NIR group. Furthermore, comparing with orthotopic injection, tail vein injection has a faster success rate of lung metastasis, and tumor metastasis in other organs can also be achieved. This may be because the direct injection of tumor cells into the tail vein will cause the tumor cells to fully form implantation in the organs after a long period of blood circulation. So this method is helpful for the exploration of metastatic tumor models such as lungs, liver, and other organs metastasis, and it is also a powerful mean to study the treatment of drugs on organ metastasis models. It is well known that when a large number of tumor cells aggregate to form tumor spheres, the cancer tends to reach an advanced stage, and most advanced cancers are incurable. Therefore, the purpose of tumor treatment is to control the tumor volume, relieve symptoms, and prolong survival on the basis of ensuring the life quality of patients.

The treatment of metastatic breast cancer is a complex process. Although there are many clinical research data on first- and second-line drugs and the regimens are relatively standardized, some limitations still exist in the recommended treatment plan for the later line due to the lack of high-level evidence-based medical evidence. Now, for seeking the optimal treatment strategy and optimal drug selection for advanced breast cancer (including dosing, dosing schedule, and markers for efficacy prediction), it is necessary to bring together all parties to jointly launch some well-designed and high-quality clinical trials. At the same time, the concept of multidisciplinary comprehensive treatment should be actively promoted, that is, a more precise and individualized comprehensive treatment plan is given to each patient, and the research results are widely extended to clinical practice, so as to optimize the treatment of patients with advanced breast cancer, and ultimately prolonging the survival time of patients and improving the life quality of patients.

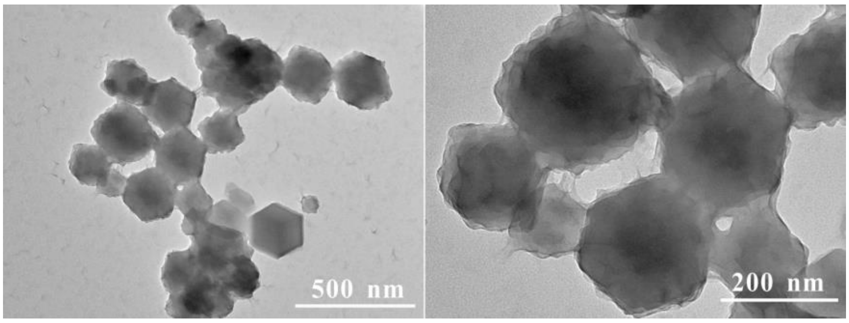


**Figure R1.** TEM of heterostructure consist of zinc-based MOF and PDA.

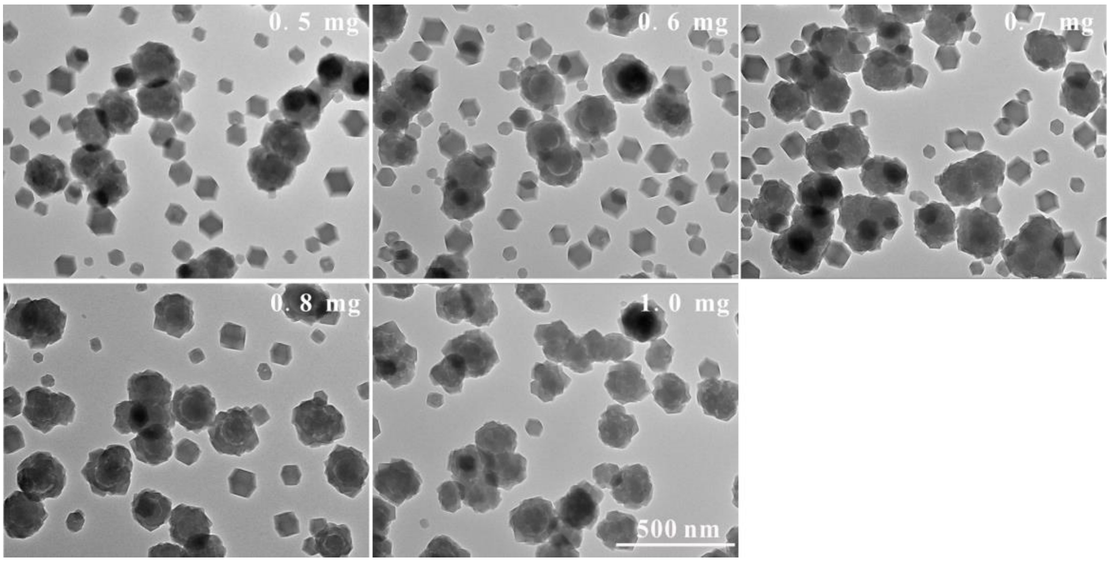


**Figure S1.** TEM of heterostructure formation under different PDA dosage.

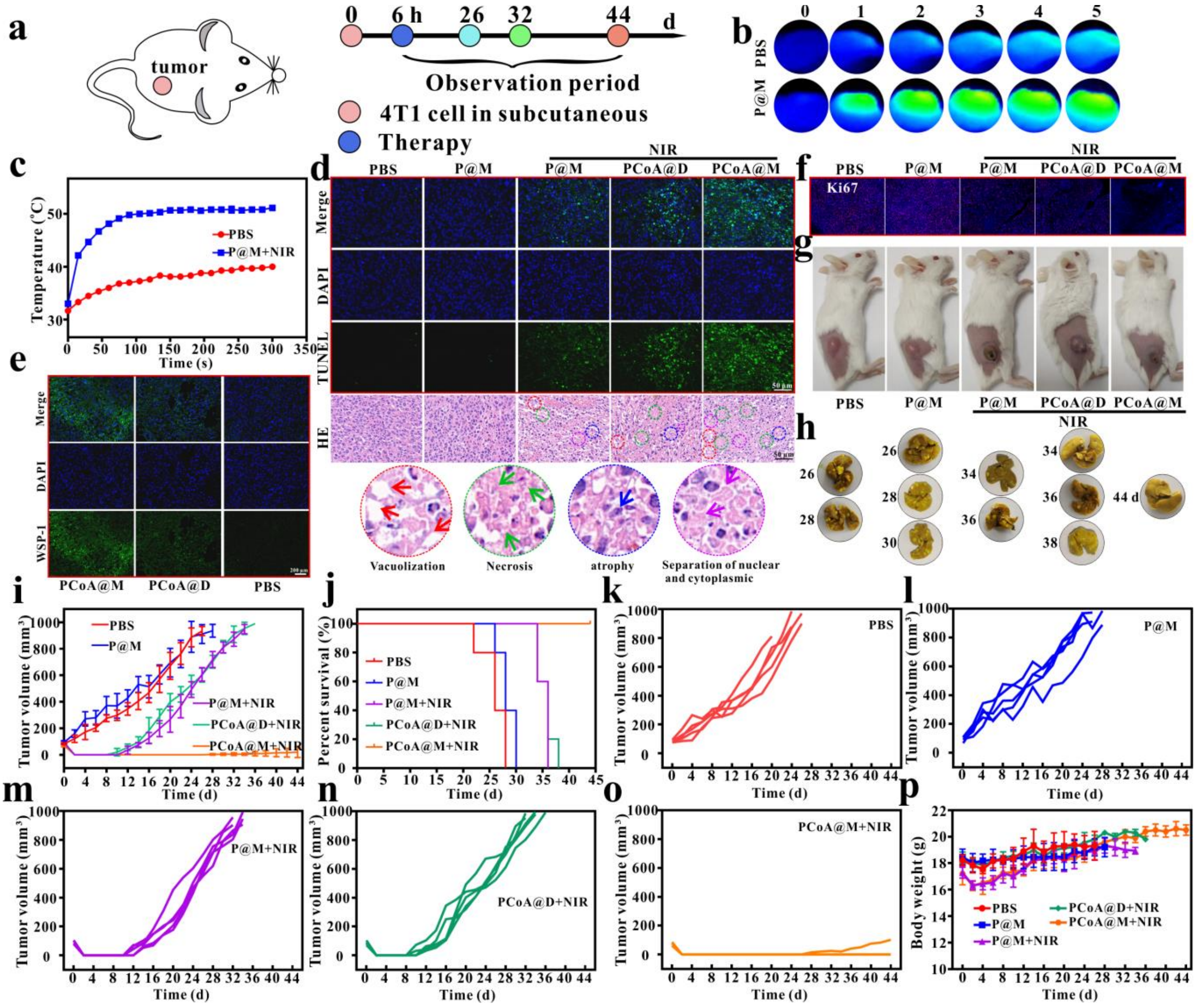

**Figure 7.** Treatment process of subcutaneous tumor (a); thermal image of tumor after injection of *in situ* 4T1 tumor-bearing mice with PBS, P@M and treated with NIR (b); temperature change curves with time (c); TUNEL and HE (d), WSP-1 (e), and Ki67 (f) immunofluorescence staining imaging of tumor site; bright-field imaging of mice after different treatments (g); the bright-field of the lung tissue of mice with subcutaneous 4T1 tumor-bearing mice after sacrifice, n=5 (h); mean tumor volume changes, n=5 (i), survival curve (j) after different treatments; changes of tumor volume in different parallel treatment groups (k-o); weight changes with time (p).

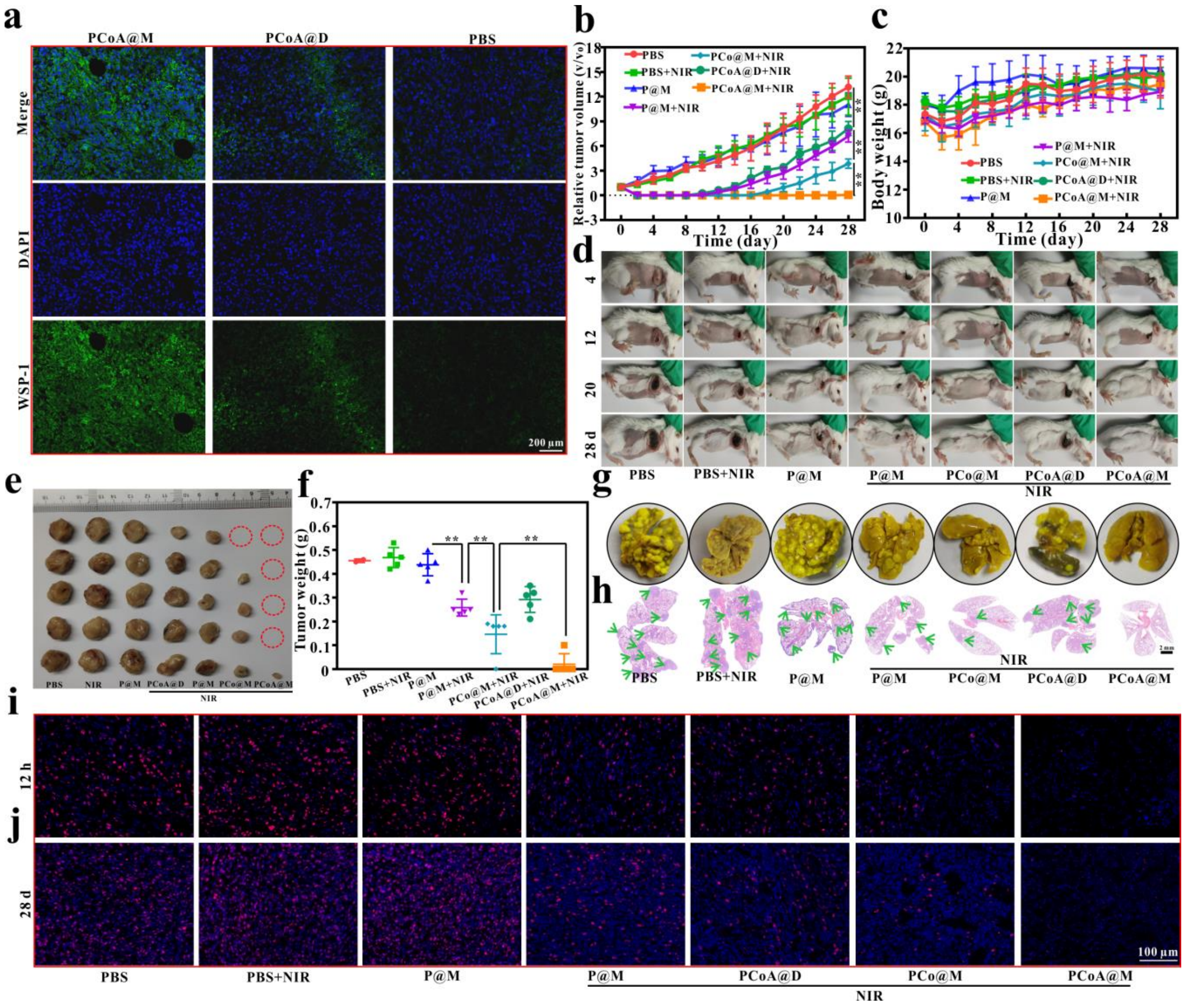


**Figure 9.** WSP-1 immunofluorescence staining imaging of tumor site after different treatments (a); relative tumor volume changes, n=5, **: p<0.01 (b), weight change (c), bright-field imaging of mice with *in situ* 4T1 tumor-bearing mice (d), the bright-field of tumors (e) and tumor quality analysis (f); the Bouins fixed lung tissue (g) of mice after sacrifice and the corresponding HE staining (h), Ki67 immunofluorescence staining imaging of tumor site under different time after different treatments (i and j).

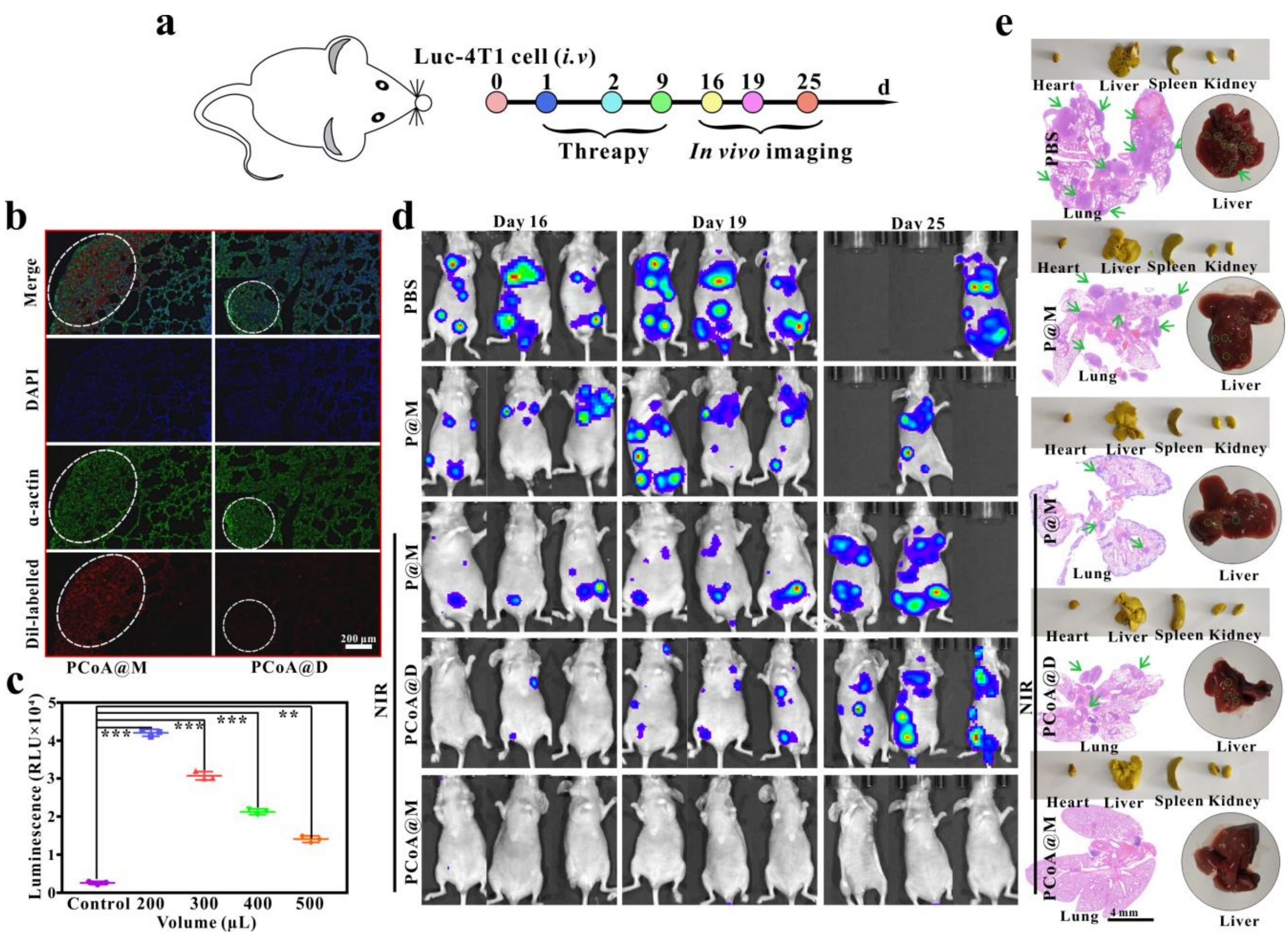


**Figure 10.** Treatment process of metastatic tumor model by injecting Luc-4T1 tumor cell into the tail vein (a); α-actin immunofluorescence imaging of lung tissues of metastatic tumor model mice after treatment with different Dil-labelled probes (b); luminescence intensity of luciferin reporter gene after Luc-4T1 cells incubation with different volumes of lysate, n=3, **: $p<0.01$ (c); bioluminescence imaging at different time points after injection of different probes into 4T1 tumor lung metastatic model mice (d); bright-field imaging of main organs fixed with Bouins (yellow), original liver and HE staining of corresponding lung tissues (e).

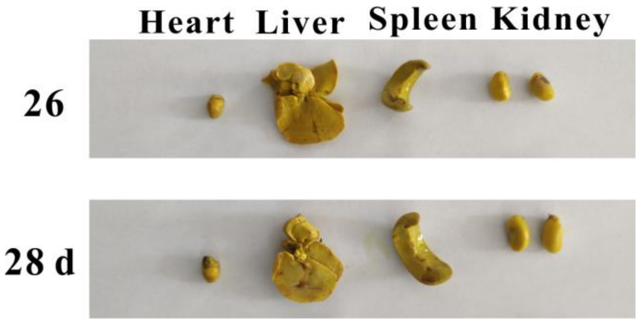


**Figure S35.** Bouins fixed organs map after PBS treatment of subcutaneous tumor.

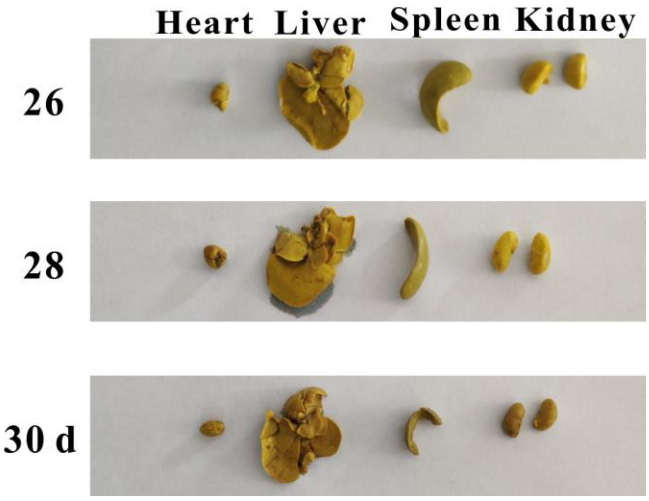


**Figure S36.** Bouins fixed organs map after P@M treatment of subcutaneous tumor.

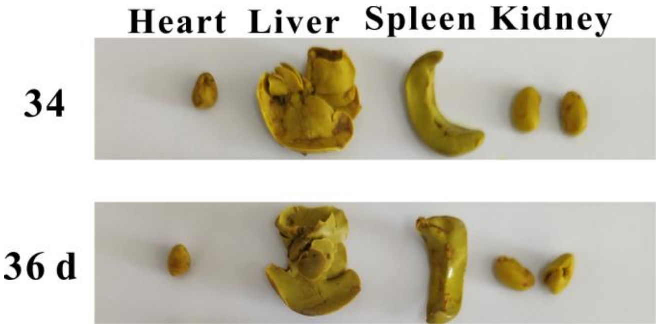


**Figure S37.** Bouins fixed organs map after P@M+NIR treatment of subcutaneous tumor.

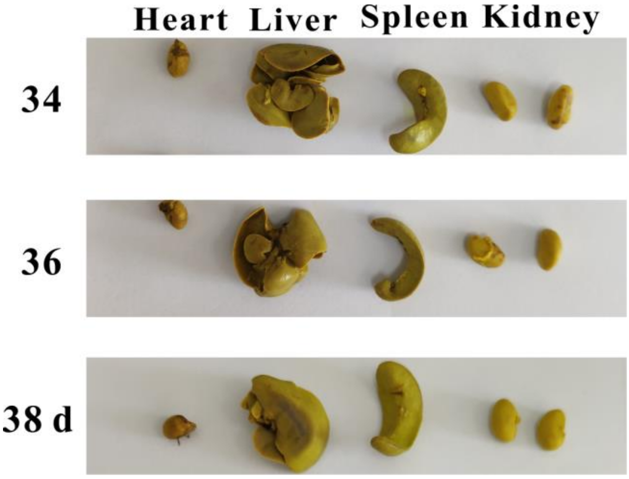


**Figure S38.** Bouins fixed organs map after PCoA@D+NIR treatment of subcutaneous tumor.

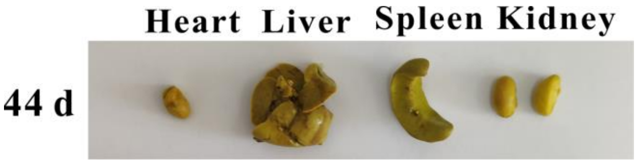


**Figure S39.** Bouins fixed organs map after PCoA@M+NIR treatment of subcutaneous tumor.

REVIEWERS' COMMENTS

Reviewer #1 (Remarks to the Author):

The questions have been answered in detail.

Reviewer #3 (Remarks to the Author):

The authors have fully addressed my concerns. I suggest the acceptance of this work.

Reviewer #1 (Remarks to the Author):
The questions have been answered in detail.

Reply: Thank you very much for the positive comments and constructive suggestions.

Reviewer #2

Reviewer #1 looked over your response to Reviewer #2 and was satisfied with your response but suggested that you add a ruler to indicate the size of the tumours in figure 8f.

Reply: Thanks for your suggestion. We have added bar to indicate the size of the tumors in fig. 7f (Fig. 8f of last version).

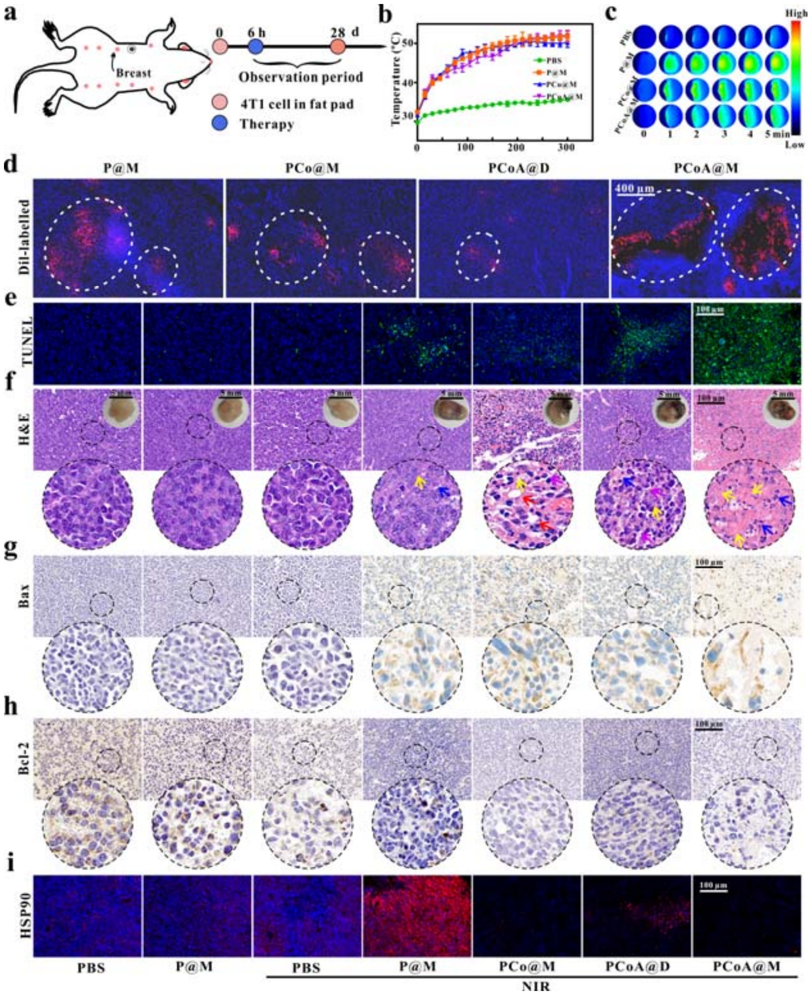


**Fig. 7** Therapy of in situ tumor in a short time. **a** Treatment process. **b, c** Temperature change curves with time and its thermal image of tumor after injection of in situ 4T1 tumor-bearing mice with PBS, P@M, PCo@M and PCoA@M and treated with NIR, colour scale: black to red represent temperature of tumor, which is from low to high. Data are presented as means ± s.d. (n=3 mice per group). **d** Fluorescence imaging of 4T1 tumor sections after injection of different Dil-labelled probes to mice. **e, f, g, h, i** TUNEL, HE, Bax, Bcl-2, and HSP90 immunofluorescence staining imaging of tumor site. Red arrow in **f**: vacuolization of cell; blue arrow: cell necrosis; yellow arrow: cell atrophy; purple arrow: separation of nuclear and cytoplasmic.

Reviewer #3 (Remarks to the Author):
The authors have fully addressed my concerns. I suggest the acceptance of this work.

Reply: Thank you very much for the positive comments and constructive suggestions.
